# Development of PCR-based markers for identification of wheat HMW glutenin *Glu*-*1Bx* and *Glu*-*1By* alleles

**DOI:** 10.1186/s12870-024-05100-w

**Published:** 2024-05-14

**Authors:** Myoung Hui Lee, Kyeong-Min Kim, Chon-Sik Kang, Mira Yoon, Ki-Chang Jang, Changhyun Choi

**Affiliations:** National Institute of Crop Science, Rural Development Administration, Wanju, 55365 Korea

**Keywords:** Hexaploid wheat, High-molecular-weight glutenin subunit, *Glu*-*1Bx*, *Glu*-*1By*, PCR-based marker

## Abstract

**Background:**

In common wheat (*Triticum aestivum* L.), allelic variations in the high-molecular-weight glutenin subunits *Glu*-*B1* locus have important effects on grain end-use quality. The *Glu*-*B1* locus consists of two tightly linked genes encoding x- and y-type subunits that exhibit highly variable frequencies. However, studies on the discriminating markers of the alleles that have been reported are limited. Here, we developed 11 agarose gel-based PCR markers for detecting *Glu*-*1Bx* and *Glu*-*1By* alleles.

**Results:**

By integrating the newly developed markers with previously published PCR markers, nine *Glu*-*1Bx* locus alleles (*Glu*-*1Bx6*, *Glu*-*1Bx7*, *Glu*-1Bx7*, *Glu*-*1Bx7* ^OE^, *Glu*-*1Bx13*, *Glu*-*1Bx14* ^(−)^ *, Glu*-*1Bx14* ^(+)^/*Bx20*, and *Glu*-*1Bx17*) and seven *Glu-1By* locus alleles (*Glu*-*1By8*, *Glu*-1By8*, *Glu*-*1By9*, *Glu*-*1By15/By20*, *Glu*-*1By16*, and *Glu*-*1By18*) were distinguished in 25 wheat cultivars. *Glu*-*1Bx6*, *Glu*-*1Bx13*, *Glu*-*1Bx14* ^(+)^/*Bx20*, *Glu*-*1By16*, and *Glu*-*1By1*8 were distinguished using the newly developed PCR markers. Additionally, the *Glu*-*1Bx13* and *Glu*-*1Bx14* ^(+)^/*Bx20* were distinguished by insertions and deletions in their promoter regions. The *Glu*-*1Bx6, Glu*-*1Bx7, Glu*-*1By9*, *Glu*-*1Bx14* ^(−)^, and *Glu*-*1By15*/*By20* alleles were distinguished by using insertions and deletions in the gene-coding region. *Glu*-*1By13*, *Glu*-*1By16*, and *Glu*-*1By18* were dominantly identified in the gene-coding region. We also developed a marker to distinguish between the two *Glu*-*1Bx14* alleles. However, the *Glu*-*1Bx14* ^(+)^ + *Glu*-*1By15* and *Glu*-*1Bx20* + *Glu*-*1By20* allele combinations could not be distinguished using PCR markers. The high-molecular-weight glutenin subunits of wheat varieties were analyzed by ultra-performance liquid chromatography and sodium dodecyl sulfate–polyacrylamide gel electrophoresis, and the findings were compared with the results of PCR analysis.

**Conclusions:**

Seven *Glu*-*1Bx* and four *Glu*-*1By* allele detection markers were developed to detect nine *Glu*-*1Bx* and seven *Glu*-*1By* locus alleles, respectively. Integrating previously reported markers and 11 newly developed PCR markers improves allelic identification of the *Glu*-*B1* locus and facilitates more effective analysis of *Glu*-*B1* alleles molecular variations, which may improve the end-use quality of wheat.

**Supplementary Information:**

The online version contains supplementary material available at 10.1186/s12870-024-05100-w.

## Background

Wheat quality is mainly based on wheat gluten protein, which has a wide range of effects on dough properties, and protein content and composition are key quality-determining parameters [1]. Gluten is a typical water-insoluble protein polymer composed of disulfide bonds and noncovalent hydrogen bonds between polymeric glutenin and monomeric gliadins [2]. Glutenin influences the strength and elasticity of wheat dough [3], whereas gliadins are responsible for its extensibility and viscosity [4]. Glutenin proteins include two major subunits, high-molecular-weight glutenin subunits (HMW-GSs) and low-molecular-weight glutenin subunits (LMW-GSs), which are the major proteins affecting the end-use quality of wheat [3, 5]. The genes encoding the HMW-GS, namely, *Glu*-*A1*, *Glu*-*B1*, and *Glu*-*D1*, are located on the long arms of chromosomes 1A, 1B, and 1D, respectively [4, 6]. Each *Glu-1* locus consists of two tightly linked genes, designated as the x-type and y-type subunits that are highly conserved, contain repeated domains, and exhibit multiple alleles [7–9]. HMW-GSs account for only about 5%–10% of grain protein, but allelic variations in HMW-GSs have been reported to account for up to 50–70% of the variation in bread-making quality [10–15]. Three, 11, and six alleles at the *Glu-A1*, *Glu-B1*, and *Glu-D1* loci, respectively, were systematically identified in 1983 by isolating HMW-GSs using sodium dodecyl sulfate–polyacrylamide gel electrophoresis (SDS-PAGE) [16]. Extensive polymorphisms were detected at all three *Glu-1* loci. The degree of polymorphism has continued to increase with analyses of different landraces, wild species, and wheat relatives [5, 17]. The Grain Genes 2.0 database identified 22 alleles for *Glu-A1*, 52 for *Glu-B1*, and 36 for *Glu-D1* [18].

Various mass spectrometry techniques and SDS-PAGE analyses have been developed and used to identify HMW-GSs [19–22]. Some HMW-GSs with similar mobilities are indistinguishable on SDS-PAGE [23, 24], but alleles such as Glu-1Bx7 and Glu-1Bx7*, Glu-1By8 and Glu-1By8*, and Glu-1Dx2 and Glu-1Ax2* show differences in surface hydrophobicity on ultra-performance liquid chromatography (UPLC) [25]. However, despite small differences in electrophoretic mobility between the Glu-1Bx7 and Glu-1Bx7* subunits, these subunits cannot be differentiated on the basis of the elution time in reversed-phase high-performance liquid chromatography (RP-HPLC) [26].

Molecular markers such as allele-specific polymerase chain reaction (AS-PCR) based on single-nucleotide polymorphisms (SNPs) and insertions and deletions (indels) have been used for fast and reliable screening of HMW-GSs in wheat breeding. Several AS-PCR markers have been developed for different HMW-GS genes, including *Glu-A1*, *Glu-B1*, and *Glu-D1* [23, 27–39]. Several indels and duplications have also been reported in the promoter regions of *Glu-B1* alleles. The 43 bp indel, 185 bp indel, and 54 bp duplication of the promoter can distinguish *Glu-1Bx6*, *Glu-1Bx7*, *Glu-1Bx13, Glu-1Bx14*, and *Glu-1Bx20* from other *Glu-1Bx* genes. *Glu-1Bx7* ^OE^ and some types of *Glu-1Bx6* and *Glu-1Bx14* with a 43 bp insertion in the promoter region [40] can be selected using a marker reported by Ragupathy et al. [33]. The difference between *Glu-1Bx7* and *Glu-1Bx7* ^OE^ is a 43 bp insertion in the promoter region and gene duplication [23, 33]. Glu-1Bx7* shows an 18 bp deletion in the coding region of the gene. Agarose gel-based DNA markers have been used to accurately discriminate the *Glu-B1* locus subunits *Glu-1Bx7* and Glu-1Bx7*, *Glu-1Bx7* and *Glu-1Bx17*, *Glu-1By9* and *Glu-1By8, Glu-1Bx6* and non-*Glu-1Bx6*, and *Glu*–*1Bx23* and Glu-1Bx23* by size polymorphisms of 18, 108, 45, 15, and 18 bp, respectively [24, 31, 32, 35, 37, 38]. In addition, AS-PCR markers have been used to discriminate between *Glu-1By8* and *Glu-1By18* using SNPs [24], and two *Glu-1By18*-specific SNP-based markers were developed by Liang et al. [34].

Identification of HMW glutenin in wheat remains a high priority in wheat-breeding programs to determine which genotypes should be used in breeding programs because of the large variations in allelic composition among varieties. Although new genotypes continue to be reported, PCR markers cannot distinguish among all genotypes. In addition, the discriminant information of previously reported markers was limited to only a few alleles. SDS-PAGE and HPLC/UPLC analyses are not only time-consuming, but in some cases size-indistinguishable, necessitating the development of PCR markers that can distinguish the genotype of a breed. In this study, we developed PCR markers to distinguish nine *Glu-1Bx* alleles and seven *Glu-1By* alleles located at the *Glu-B1* locus by using several specific SNPs and indels in 25 wheat varieties. These markers could be useful for marker-assisted selection of *Glu*-*B1* alleles in wheat quality improvement programs.

## Methods

### Plant materials

A total of 25 wheats were provided by the Korean Agricultural Culture Collection (KACC) (htpp://genebank. rda. go. kr). Formal identification of wheat varieties used in this study were performed by KACC. Three IT numbers (registration numbers for plants at the NARO Institute of Agrobiological Sciences National) were assigned to the Avalone cultivar. Information of HMW-GS allele compositions and IT numbers is listed in Table 1. The respective countries of origin and published and corrected *Glu-B1* alleles are also indicated (Table 1).

**Table 1:** List of wheat varieties used in this study. The respective countries of origin and published and corrected *Glu-B1* alleles are indicated

No	cultivar	Origin	IT no^a^	*Glu-B1* (reported)	*Glu-B1* (corrected)	InDel (Promoter)
1	Avalon	UK	IT176822	6 + 8	6 + 8*	–
2	Avalon	UK	IT115944	6 + 8	6 + 8*	–
3	Avalon	UK	IT014649	6 + 8	6 + 8*	–
4	Keumkang	Korea	IT213100	7 + 8	7 + 8	–
5	Hanback	Korea	IT311644	7 + 8	7 + 8	–
6	Alchan	Korea	IT175574	7 + 8	7 + 8	–
7	Gobun	Korea	IT214681	7 + 9	7* + 9	–
8	Eunpa	Korea	IT175521	7 + 9	7* + 9	–
9	Anbaek	Korea	IT213245	7 + 9	7* + 9	–
10	Cheyenne	USA	IT011154	7* + 9	7* + 9	–
11	Klein Cacique	Argentina	IT234987	7* + 8	7* + 8	–
12	Chisolm	USA	IT230956	7^OE^ + 8*	7^OE^ + 8*	+ 43 bp
13	MT8191	USA	IT230937	7^OE^ + 8*	7^OE^ + 8*	+ 43 bp
14	KS85WGRC01	USA	IT230965	7^OE^ + 8*	7^OE^ + 8*	+ 43 bp
15	Anbaek	Korea	IT246750	13 + 16	13 + 16	-54 bp
16	Baekjoong	Korea	IT227093	13 + 16	13 + 16	-54 bp
17	Joeun	Korea	IT213101	13 + 16	13 + 16	-54 bp
18	Troll	Germany	IT206475	14 + 15	14^(−)^ + 15	–
19	Hanno	Germany	IT206477	14 + 15	14^(−)^ + 15	–
20	Imbros	Germany	IT206478	14 + 15	14^(−)^ + 15	–
21	Joongmo2008	Korea	IT269492	17 + 18	17 + 18	–
22	Suwon92	Korea	IT246989	17 + 18	17 + 18	–
23	Suwon105	Korea	IT246973	17 + 18	17 + 18	–
24	Suwon15	Korea	IT246979	20 + 20	20 + 20	+ 185 bp
25	Suwon28	Korea	IT246969	20 + 20	20 + 20	+ 185 bp
26	Suwon42	Korea	IT246970	20 + 20	20 + 20	+ 185 bp
27	RANEE	UNK	IT176783	14^(+)^ + 15	14^(+)^ + 15	+ 185 bp

^a^IT no: registration number for plants at the NARO Institute of Agrobiological Sciences National

### Genomic DNA extraction

A total of 27 hexaploid wheat (*Triticum aestivum* L.) plants were grown in a Petri dish with moisture paper. Genomic DNA was extracted from the leaf tissue using the Higene™ Genomic DNA Prep kit (solution type; BioFACT, Korea). The quality of the genomic DNA was assessed using a NanoDrop 1000 spectrophotometer (Thermo Scientific, MA, USA), and the integrity of the DNA was checked using 1% agarose gel electrophoresis.

### Sequence alignment and phylogenetic tree analysis

Sequences of *Glu-1Bx6* (KX454509.1), *Glu-1Bx7* (BK006773.1), Glu-1Bx7* (X13927.3), *Glu-1Bx13* (EF540764.1), *Glu-1Bx14* (AY367771.1 and KF733216.1), Glu-1Bx17* (KF547469.1), *Glu-1Bx17* (KC254854.1), *Glu-1Bx20* (AJ437000.2), *Glu-1Bx23* (AY553933.1), and Glu-1Bx23* (KF995273.1) from *Glu-1Bx* and sequences of *Glu-1By8* (JN255519.1), *Glu-1By9* (X61026.1), *Glu- 1By15* (EU137874.1), Glu-1By15* (KJ579440.1), *Glu-1By16* (EF540765.1), *Glu-1By18* (KF430649.1), and *Glu-1By20* (KU886033.1) from *Glu-1By* were collected from National Center for Biotechnology information (NCBI). The gene sequence of Glu-1By8* was not available in the public databases. In this study, *Glu*-*1Bx14* alleles with accession numbers KF733216.1 and AY367771.1 were named *Glu*-*1Bx14* ^(−)^ and *Glu*-*1Bx14* ^(+)^, respectively. Multiple sequence alignments of the full-length nucleotide sequences of the *Glu-1Bx* and *Glu-1By* alleles were performed using Clustal Omega (www.ebi.ac.uk/Tools/msa/Clustalo). Phylogenetic tree was generated using Molecular Evolutionary Genetics Analysis version 11 (MEGA11) [41]. Bootstrap values were calculated on the basis of 1,000 replications.

### PCR conditions

PCR was performed under the following conditions: 5 min at 95 °C, 30 cycles of 30 s at 94 °C, 30 s at 55–68 °C, and 30 s at 72℃ on a thermal cycler (Applied Biosystem, MA, USA) with 20 μL reaction volumes for each sample containing 50 ng of template DNA, 10 pmol of each primer with 1 × master mix solution (i-MAX II DNA polymerase, Intronbio, Korea) or 0.1 μL (5 units/μL) of Takara Ex Taq DNA polymerase, 1.6 μL of dNTP mixture (2.5 mM each), and 1 × Ex Taq Buffer (Mg2^+^ free) (Takara, Japan). PCR products were electrophoresed on 1%–3% agarose gels, and visualized using a Davinch-K Gel imaging system (Davinch-K, Korea). The primers and PCR conditions are listed in Table 2.

**Table 2:** Primers used in this study for detection of *Glu-1Bx* alleles

PS	Gene	Detection region	Marker name	Type of Primer	Sequence (5’-3’)	AT		Agarose gel (%)	Reference
						i-MAX II	Ex Taq		
PS1	*Glu*-*1Bx7*/*17*	CDS	MHBx717-F	SNP	CACTGAGATGGCTAAGCGCC	66℃	66℃	1%	this study
			MHBx717-R		GATCTTGTTGCCCTTGTCC				
PS2	*Glu*-*1Bx7* ^OE^	Promoter	MAR-F	Indel (+ 43 bp)	CCTCAGCATGCAAACATGCAGC	66℃	68℃	1%	[23]
			MAR-R		CTGAAACCTTTGGCCAGTCATGTC				
PS3	*Glu*-*1Bx7* ^OE^	Junction	TaBAC1215C06-F517	Duplication	ACGTGTCCAAGCTTTGGTTC	60℃	68℃	1%	[33]
			TaBAC1215C06-R964		GATTGGTGGGTGGATACAGG				
PS4	*Glu*-*1Bx7* ^OE^	Junction	TaBAC1215C06-F24671	Duplication	CCACTTCCAAGGTGGGACTA	60℃	68℃	1%	[33]
			TaBAC1215C06-R2551		TGCCAACACAAAAGAAGCTG				
PS5	*Glu*-*1Bx20*	Promoter	MHBx-185-F	Indel (+ 185 bp)	GATAAGGCCAACGAGAGAAGAA	60/66℃	59℃	1%	this study
			MHBx-185-R		GATCTTAGTAAATCCGCGTCAAATAG				
PS6	*Glu*-*1Bx20*	CDS	cauBx752-F	Indel (+ 185 bp)	AGGGGCAGGGAAGAAACACT	66℃	66℃	1%	[31]
			cauBx752-R		CCAGGCAACACAAATCCATG				
PS7	*Glu*-1Bx7*	CDS	Bx7F	Indel (-18 bp)	CAACTTCTTCACAGCAGT	60℃	66℃	3%	[35] (modified)
			Bx7R		TGCGCCTTTGCCACCTTTAG				
PS8	*Glu*-*1Bx17*	CDS	cauBx642-F	Indel (-108 bp)	GGGCAATCGGGGTACTTCC	60℃	59℃	1%	[31]
			cauBx642-R		CCCTTGTCTTGGCTGTTGTC				
PS9	*Glu*-*1Bx6*	CDS	BX7-F	InDel (+ 15 bp)	CACTGAGATGGCTAAGCGCC	60℃	66℃	3%	[32]
			BX7-R		GCCTTGGACGGCACCACAGG				
PS10	*Glu*-*1Bx6*	CDS	MHBx6-F	SNP	CACTGAGATGGCTAAGCGCC	67℃	68℃	1%	this study
			MHBx6-R		GATCTTGTTGCCCTTGTTC				
PS11	*Glu*-*1Bx13*	Promoter	MHpro13-F	Indel (-54 bp)	GGGCTTTAGGAGAGATGGTTTAT	60℃	59℃	1%	this study
			MHpro13-R		GCCTATGAAGAAAGCGTGAGA				
PS12	*Glu*-*1Bx13*	CDS	MHBx13-F	SNP	GGCACGAGGCAATACGAGCG	66℃	66℃	1%	this study
			MHBx13-R		TGATCTTGTTGCTCTTGTCT				
PS13	*Glu*-*1Bx20*	CDS	MHBx20-F	SNP	GTGTACTACCCAACTTCTCT	66℃	68℃	1%	this study
			MHBx20-R		CCTGGCTGTTGCTCATGTCT				
PS14	*Glu*-*1Bx14* ^(−)^	CDS	MHBx14(-)-F	Indel (-18 bp)	TACTACCCAACCTCTCCACA	64℃	66℃	3%	this study
			MHBx14(-)-R		CTGTTGCTCTTGTGCTGATTG				

*PS* Primer set, *CDS* Coding sequence, *AT* Annealing temperature, *new* Newly developed marker

### Glutenin protein analysis using SDS-PAGE

Glutenin was extracted from single wheat grains using a previously reported HMW-GS extraction protocol [42, 43]. Protein (approximately 10 μg) extracted from grains of different varieties was separated using 10% SDS-PAGE and visualized using Coomassie Brilliant Blue R + 250 staining solution (Bio-Rad, CA, USA).

### Glutenin analysis using ultra-performance liquid chromatography

Glutenin analysis was performed by crushing single grains of wheat, and the glutenin extraction method was performed as described previously [42, 43]. The extracted glutenin was analyzed using a UPLC system (Alliance e2695, Waters Corp., MA, USA) with an ACQUITY UPLC Peptide BEH C18 column (300A, 1.7 μm, 2.1 mm × 50 mm) and a photodiode array detector. The mobile phases were H_2_O containing 0.1% trifluoroacetic acid (A) and acetonitrile containing 0.1% trifluoroacetic acid (B). The injection volume of the dissolved samples was 3 μL and the flow rate was 0.55 μL/min. The solvent gradient was changed from 21 to 47% (B) from 0 to 30 min, and the column and sample temperatures were set to 55 °C and 10 °C, respectively.

## Results

### Sequence alignment and phylogenetic analysis of Glu-1Bx subunits

**Fig. 1:**
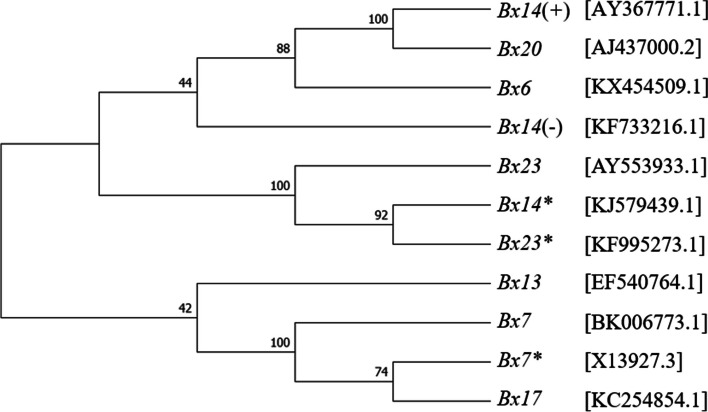
Phylogenetic tree of 11 HMW-GS *Glu-1Bx* alleles constructed using complete coding DNA sequences. HMW-GS *Glu-1Bx* alleles included *Glu-1Bx6* (KX454509.1), *Glu-1Bx7* (BK006773.1), Glu-1Bx7* (X13927.3), *Glu-1Bx13* (EF540764.1), *Glu-1Bx14* ^(−)^ (KF7333216.1), *Glu-1Bx14*.^(+)^ (AY367771.1), Glu-1Bx14* (KJ579439.1), *Glu-1Bx17* (KC254854.1), *Glu-1Bx20* (AJ437000.2), *Glu-1Bx23* (AY553933.1), and Glu-1Bx23* (KF995273.1)

The nucleotide sequences of 11 *Glu-1Bx* subunits (*Glu-1Bx6*, Glu-1Bx7*, *Glu-1Bx7*, *Glu-1Bx13*, Glu-1Bx14*, two *Glu-1Bx14*, *Glu-1Bx17*, *Glu-1Bx20*, Glu-1Bx23*, and *Glu-1Bx23*) were aligned using Clustal Omega (Fig. S1). Among these, the base sequences of *Glu*-*1Bx14* (AY367771.1) and Glu-1Bx20 showed a high degree of identity (99%). The base sequences of *Glu*-1Bx14* and *Glu*-1Bx23* also showed high identity (99%). In contrast, the base sequences of the two accessions of *Glu-1Bx14* showed a relatively low identity of 96% (Fig. S1). The complete coding sequences of the 11 HMW-GS genes were used to construct a neighbor-joining tree to investigate the phylogenetic relationships among the HMW-GS *Glu-1Bx* genes (Fig. 1). The genes encoding *Glu*-*1Bx14* ^(+)^ and *Glu*-*1Bx20*, *Glu*-1Bx14* and *Glu*-1Bx23*, and *Glu*-1Bx7* and *Glu*-*1Bx17* were closely related (Fig. 1), indicating similar structural features and close phylogenetic evolutionary relationships.

### Detection of Glu-1Bx subunits

**Fig. 2:**
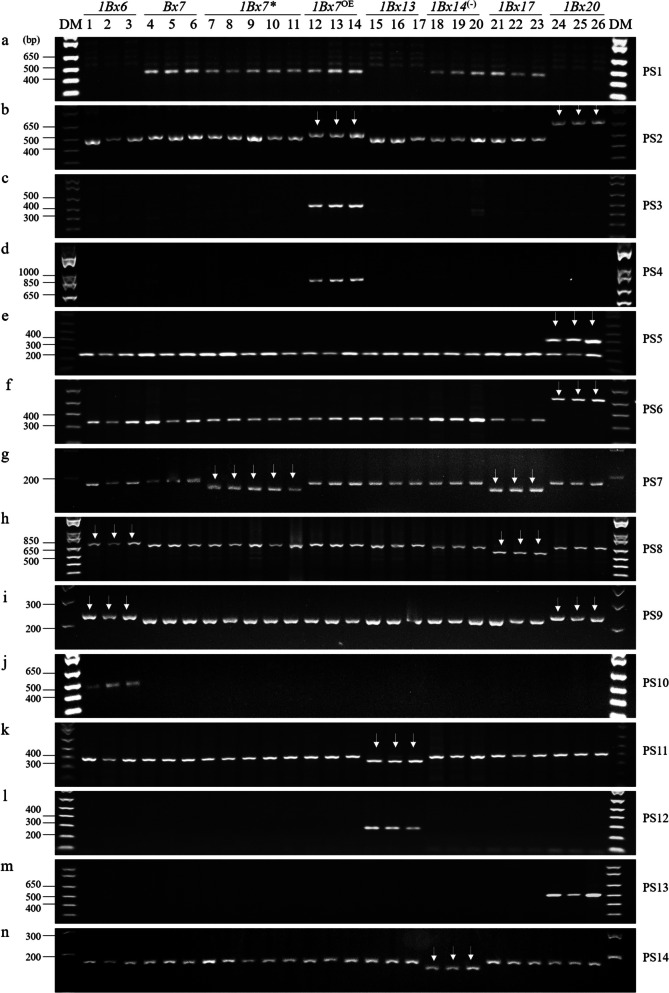
PCR analysis of *Glu-1Bx* alleles in wheat varieties. The numbers above the figure are the same as the variety numbers in Table 1. The primer set numbers in the figure are the same as in Table 2. DM, DNA size marker; PS, primer set. Arrows indicate indels

Eight *Glu-1Bx* subunits were primarily investigated using PCR-based markers in the 24 cultivars (Table 1). Previously reported molecular markers identifying the *Glu-1Bx7* allele were > 2,000 bp (2,373/2,500 bp) in size [36, 44]. Therefore, in this study, we designed a PCR primer set (PS1) to detect *Glu-1Bx7* homologs (*Glu-1Bx7*, Glu-1Bx7*, and *Glu-1Bx7* ^OE^). However, *Glu-1Bx17* (Joongmo2008, Suwon105, and Suwon92) and *Glu-1Bx14* ^(−)^ (Troll, Hanno, and Imbros) were also detected in PS1 (Fig. 2a). Next, to examine *Glu-1Bx7* ^OE^, we detected a 43 bp insertion in the promoter region by using the marker reported by Butow et al. [23] (PS2, Fig. 2b). In a previous report, a 43 bp insertion in the promoter was observed in some types of *Glu-1Bx6* and *Glu-1Bx14*. Additionally, some types of *Glu-1Bx14* and *Glu-1Bx20* contain a 185 bp insertion in the promoter region [40, 45]. Therefore, the PCR products were expected to be of four sizes: 520 bp (non-43 bp insertion), 563 bp (43 bp insertion), 705 bp (185 bp insertion), and 748 bp (43 bp and 185 bp insertions). However, a 43 bp insertion was detected in the promoter region of *Glu-1Bx7* ^OE^ (Chisolm, MT8191, and KS85WGRC01), but not in *Glu-1Bx6* (Avalon) or *Glu-1Bx14* ^(−)^ (Troll, Hanno, and Imbros). In addition, in *Glu-1Bx20* (Suwon15, Suwon28, and Suwon42), only 185 bp was detected without the 43 bp insertion (Fig. 2b). Next, two *Glu-1Bx7* gene duplication markers (left- and right-junction markers) were tested to confirm the cultivars with the 43 bp insertion contained *Glu-1Bx7* ^OE^ [33]. Gene duplication was detected in three cultivars (Chisolm, MT8191, and KS85WGRC01), suggesting that they contained *Glu-1Bx7* ^OE^ (Fig. 2c, d). Therefore, we developed a PCR-based marker that detects a 185 bp insertion without a 43 bp insertion in the promoter (Fig. 2e, Fig. S2). Similar to the marker reported by Xu et al. [31] (PS6, Fig. 2f), this marker showed good detection ability for the 185 bp insertion in *Glu-1Bx20* (Suwon15, Suwon28, and Suwon42).

One of the *Glu-1Bx7* variants, Glu-1Bx7*, is characterized by an 18 bp deletion in the repeat domain corresponding to an additional hexapeptide motif [46], and a Glu-1Bx7* detection marker using this 18 bp deletion has been reported by Espi et al. [35]. The reverse primer for Glu-1Bx7* reported by Espi et al. [35] was modified in this study. However, *Glu-1Bx17* also lacks an 18 bp repeat domain corresponding to an extra hexapeptide motif and was detected by PS7 (Fig. 2g). In addition to the 18 bp deletion, *Glu-1Bx17* is characterized by a 108 bp deletion in the coding sequence; therefore, a marker capable of distinguishing *Glu-1Bx17* with a 108 bp deletion from other *Glu-1Bx* genes has been reported [28, 31] (PS8, Fig. 2h). With this marker, *Glu-1Bx6* was detected to be 45 bp larger than other *Glu-1Bx* genes except for *Glu-1Bx17*. Additionally, we found that Gobun, Eunpa, and Anbaek contained Glu-1Bx7* instead of *Glu-1Bx7* [47, 48] (Fig. 2g).

### Detection of the Glu-1Bx6 subunit

*Glu-1Bx6* was detected as a larger PCR fragment than the other *Glu-1Bx* alleles in PS8. Additionally, *Glu-1Bx6* was distinguished from other *Glu-1Bx*, except for *Glu-1Bx20*, by a 15 bp insertion in the coding regions [32] (PS9, Fig. 2i). However, *Glu-1Bx20* also contained a 15 bp insertion in the coding region (Fig. 2i). Therefore, a dominant marker for *Glu-1Bx6* was developed to detect a size of 457 bp using SNP (PS10). As shown in Fig. 2j, the PCR products from three Avalon cultivars were detected using this marker.

### Detection of Glu-1Bx13 alleles

The tandem 54 bp duplication at position –400 of the promoter region contains a “cereal box”, which has been implicated in seed-specific expression [49]. However, the promoter region of *Glu-1Bx13* does not contain a 54 bp replication [40]. To discriminate of *Glu-1Bx13* from other *Glu-1Bx* alleles in the promoter, we developed a co-dominant primer set to detect *Glu-1Bx13* and non-*Glu-1Bx13* with sizes of 365 and 419 bp, respectively (PS11, Fig. 2k). Three cultivars (Jeokjoong, Baekjoong, and Joeun) showed a 54 bp deletion in comparison with the other cultivars. In addition, we developed a PCR primer set to detect the *Glu-1Bx13* coding region. This primer set dominantly detected *Glu-1Bx13* with a size of 254 bp (PS12, Fig. 2l).

### Detection of the Glu-1Bx20 subunit

*Glu-1Bx20* contains a 185 bp insertion in the promoter region and was detected by PS2, PS5, and PS6. However, some types of *Glu*-*1Bx14* have been reported to contain 185 bp insertion in the promoter region [40, 45]. Therefore, we developed a set of primers (PS13) for the detection of the *Glu-1Bx20* coding region with a size of 501 bp using an SNP (Fig. 2m). This primer set specifically detected *Glu*-*1Bx20* in three cultivars (Suwon15, Suwon28, and Suwon42).

### Detection of Glu-1Bx14 subunits

**Fig. 3:**
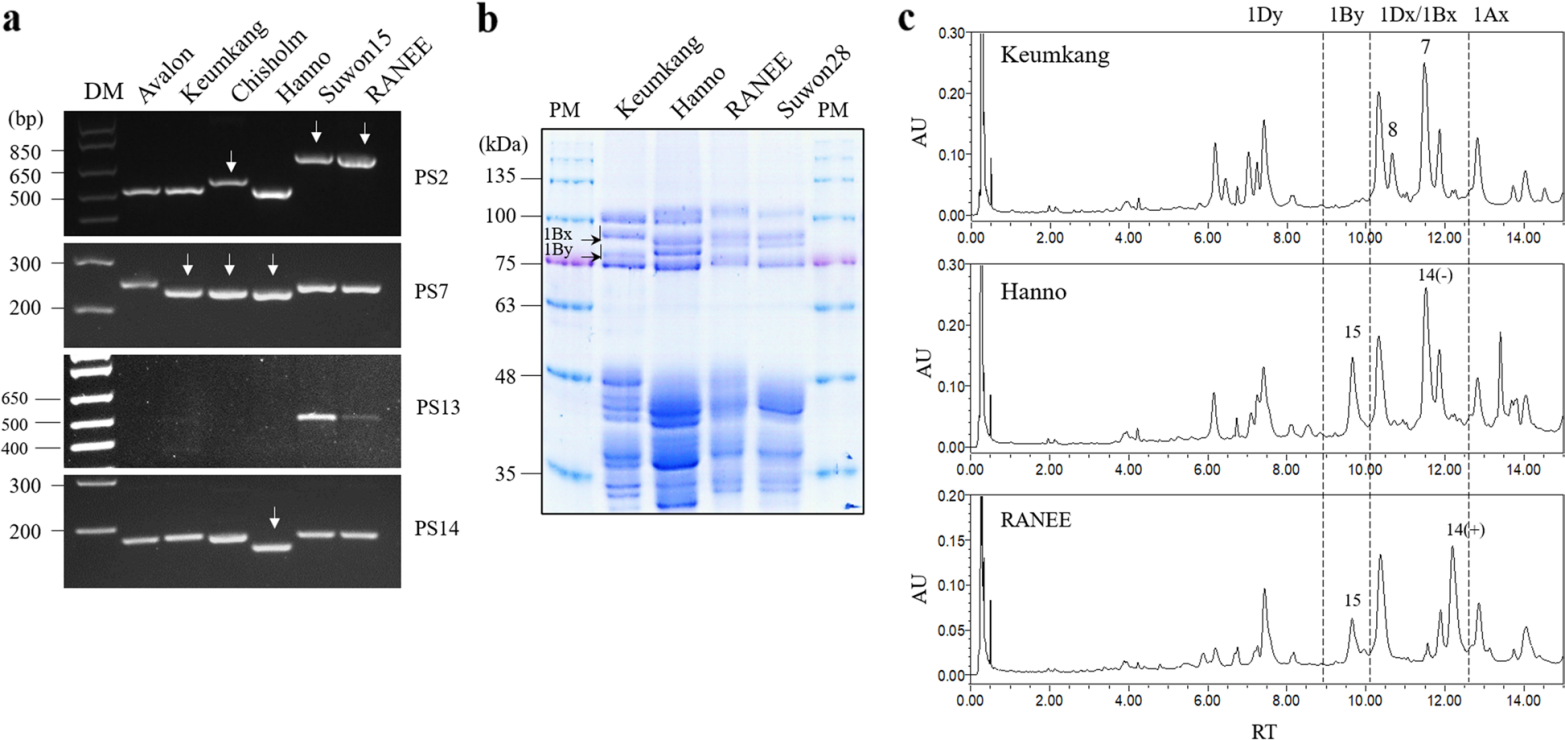
Analysis of two *Glu-1Bx14* alleles. (A), PCR amplification of six cultivars. DM, DNA size marker. Allows indicate indels. (B), HMW-GS from four cultivars identified by SDS-PAGE. PM, protein size marker; KK, Keumkang; Ha, Hanno; RA, RANEE. Arrows indicate Glu-1Bx and Glu-1By. (C), HMW-GS from three cultivars identified by UPLC. AU, arbitrary units; RT, retention time

Two German bread wheat cultivars, Hanno and Imbros, carry *Glu-1Bx14* ^(−)^ along with the *Glu-1By15* subunits [21]. The *Glu-1Bx14* ^(+)^ sequence was highly similar with to that of *Glu-1Bx20* [31, 39]. However, the three variants (Troll, Hanno, and Imbros) not only did not contain insertions of 43 bp and 185 bp in the promoter region but were also specifically detected by PS1 in the detection of *Glu-1Bx7* homologs (Fig. 2). Since two accession numbers were registered in NCBI, we compared the sequences corresponding to the accession numbers AY367771 and KF733216 (Fig. S3). These two accession numbers showed 94% similarity in the nucleotide sequence, and the KF733216 sequence showed an 18 bp deletion in the coding sequence in comparison with other *Glu*-*1Bx* alleles. Next, we developed a PCR marker for detection of *Glu*-*1Bx14* ^(−)^ using the 18 bp indel (PS14, Fig. 2n). This marker differentiated *Glu*-*1Bx14* ^(−)^ from *Glu*-*1Bx7* homologs and *Glu*-*1Bx17*. Three cultivars (Troll, Hanno, and Imbros) showed a 18 bp deletion. We also tested RANEE (*Glu-1Bx14* + *Glu-1By15*), a cultivar containing *Glu-1Bx14*. A 185 bp insertion was detected in the promoter region of RANEE, similar to Suwon15 (PS2), but an 18 bp deletion was not detected in the coding region (PS7, PS14), indicating *Glu-1Bx14* ^(+)^ (Fig. 3a). However, both *Glu-1Bx20* and *Glu*-*1Bx14* ^(+)^ were detected using the *Glu*-*1Bx20* detection marker PS13. *Glu*-*1Bx14* ^(+)^ and *Glu*-*1Bx20* contain several SNPs in the coding sequence but have 99% nucleotide sequence identity; therefore, in this study, several AS-PCR markers were tested, but *Glu*-*1Bx14* ^(+)^ and *Glu*-*1Bx20* could not be distinguished.

In SDS-PAGE, the size of Glu-Bx14^(−)^ was different from that of Glu-1Bx14^(+)^ (Fig. 3b), and the extraction time of Glu-Bx14^(−)^ was faster than of Glu-Bx14^(+)^ (Fig. 3c).

### Sequence alignment and phylogenetic analysis of Glu-1By subunits

**Fig. 4:**
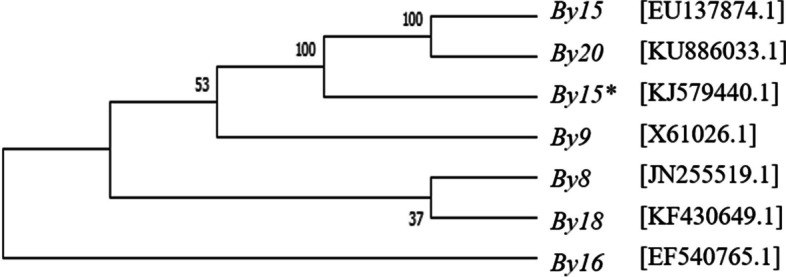
Phylogenetic tree of seven HMW-GS *Glu-1By* alleles constructed using complete coding DNA sequences. HMW-GS alleles included *Glu-1By8* (JN255519.1), *Glu-1By18* (KF430649.1), *Glu-1By9* (X61026.1), *Glu-1By15* (EU137874.1), Glu-1By15* (KJ579440.1), *Glu-1By16* (EF540765.1), and *Glu-1By20* (KU886033.1)

The nucleotide sequences of seven *Glu*-*1By* subunits (*Glu*-*1By8*, *Glu*-*1By9*, *Glu*-*1By15*, *Glu*-1By15*, *Glu*-*1By16*, *Glu*-*1By18*, and *Glu*-*1By20*) were aligned (Fig. S4). Of these, *Glu*-*1By8* and *Glu*-*1By18,* and *Glu*-*1By15* and *Glu*-*1By20* were highly conserved with 99% sequence identity. To investigate the phylogenetic relationships among the HMW-GS *Glu-1By* genes, the complete coding sequences of the seven HMW-GS genes were used to construct a neighbor-joining tree (Fig. 4). The genes encoding *Glu*-*1By15* and *Glu*-*1By20* were more closely related. Additionally, the genes encoding *Glu*-*1By8* and *Glu*-*1By18* were also more closely related, indicating similar structural features and close phylogenetic evolutionary relationships (Fig. 4).

### Detection of Glu-1By8 and Glu-1By18 subunits

**Fig. 5:**
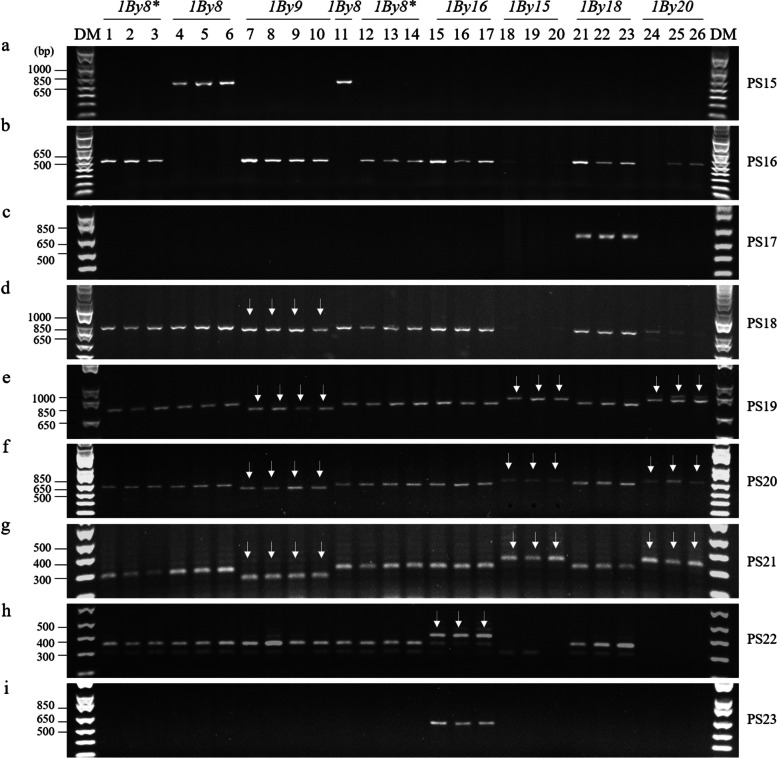
PCR analysis of *Glu-1By* alleles in wheat varieties. The numbers above the figure are the same as the variety numbers in Table 1. The primer set numbers in the figure are the same as in the Table 3. DM, DNA size marker; PS, primer set. Arrows indicate indels

Lei et al. [28] reported two markers (PS15 and PS16) that could differentiate *Glu*-*1By8* from *Glu*-1Bx8* and *Glu*-*1Bx18* alleles, which are generally difficult to distinguish using SDS-PAGE*.* A pair of AS-PCR primers (PS15) discriminated *Glu*-*1By8*, which produced a 527 bp fragment, while non-*Glu*-*1By8* alleles showed negative results for PS15 (Fig. 5a). Previously, a pair of AS-PCR primers (PS16) was used to discriminate between *Glu*-*1By18* and *Glu*-*1By8* genes. In this study, no PCR product was detected from *Glu*-*1By8* using this primer set; however, a PCR product of 527 bp was amplified from the other *Glu-1By* alleles, but the amplified *Glu-1By15* and *Glu-1By20* PCR products were weak (Fig. 5b). These markers could distinguish *Glu-1By8* from other *Glu-1By* alleles, but could not distinguish Glu-1By8* and *Glu-1By18* from other *Glu-1By* alleles (Fig. 5a, b). Therefore, in this study, we developed a set of specific primers to detect a 543 bp PCR product to distinguish *Glu-1By18* from other *Glu-1By* alleles (PS17, Fig. 5c). With PS17, a single band of 543 bp was specifically detected in three cultivars (Joongmo2008, Suwon92, and Suwon105) containing *Glu-1By18*.

**Table 3:** Primers used in this study for detection of *Glu-1By* alleles

PS	Gene	Detection region	Marker name	Type of primer	sequence (5’-3’)	AT		Agarose gel (%)	Reference
						i-MAX II	Ex Taq		
PS15	*Glu*-*1By8*	CDS	ZSBy8F5	SNP (*By8* dominant)	TTAGCGCTAAGTGCCGTCT	69℃	68℃	1%	[24]
			ZSBy8R5		TTGTCCTATTTGCTGCCCTT				
PS16	Multi-gene	CDS	By18-SNP-F	SNP (*By8* negative)	TTAGCGCTAAGTGCCGTCC	66℃	66℃	1%	[24]
			By18-SNP-R		TTGTCCTATTTGCTGCCCTT				
PS17	*Glu-1By18*	CDS	MHBy18-F	SNP	CAACTTCTCCACAACAGTCG	68℃	69℃	1%	this study
			MHBy18-R		GTCCTAGTTGGTGTCCTTGTT				
PS18	*Gu*-*1By9*/*15*/*20*	CDS	By01-F	Indel (-45 bp)	GGGGCCATATTATCCAGGCC	66℃	68℃	1%	[37]
			By02-R		TTTGTCCTAGCTGTCCTATTTGT				
PS19	*Gu*-*1By9*/*15*/*20*	CDS	ZSBy9aF1	Indel (-45 bp)	TTCTCTGCATCAGTCAGGA	60℃	66℃	1%	[24]
			ZSBy9aR3		AGAGAAGCTGTGTAATGCC				
PS20	*Gu*-*1By9*/*15*/*20*	CDS	MHBy9-F2	Indel	GGGCAGCAAATAGGACAAGA	ND	66℃	1%	this study
			MHBy9-R		TTTGTCCTAGCTGTCCTATTTGT				
PS21	*Gu*-*1By9*/*15*/*20*	CDS	MHBy1520-F	Indel	TCTCCACAGCAGCTAGGACG	66℃	66℃	1%	this study
			MHBy1520-R		GCTGTTGAGAAGCTGGGTAC				
PS22	*Glu*-*1By16*	CDS	ZSBy9F2	Indel	GCAGTACCCAGCTTCTCAA	68℃	66℃	1%	[24]
			ZSBy9R2		CCTTGTCTTGTTTGTTGCC				
PS23	*Glu*-*1By16*	CDS	MHBy16-F	SNP	GGGCAGCAAATAGGACAAGA	68℃	68℃	1%	this study
			MHBy16-R		TTGTCCTACCTGCTGCAGG				

*PS* Primer set, *CDS* Coding sequence, *ND* Not determined, *AT* Annealing temperature, *new* newly developed marker

### Detection of Glu-1By9 and Glu-1By15/20 subunits

Two specific primer sets (PS18 and PS19) for *Glu-1By9* allele detection have been reported to assay a 45 bp deletion [28, 37]. In this study, PS18 identified a PCR product that was 45 bp smaller in *Glu-1By9* than in non-*Glu-1By9,* and two weak bands were detected for the *Glu-1By15* and *Glu-1By*20 alleles (Fig. 5d). PS19 also identified a PCR product 45 bp smaller in *Glu-1By9* than in non-*Glu-1By9*. One and two larger bands were detected for *Glu-1By15* and *Glu-1By20* alleles, respectively (Fig. 5e). These two primer sets detected a size of approximately 900 bp, and prolonged electrophoresis was required to distinguish the indels. In addition, the nucleotide sequences of *Glu-1By15* and *Glu-1By20* were highly similar and could not be distinguished. Therefore, in this study, we designed two additional primer sets to distinguish between *Glu*-*1By15* and *Glu*-*1By20*, which produced a smaller PCR product with a 45 bp deletion (PS20–PS21, Fig. 5f-g). These markers also showed a 45 bp indel in *Glu-1By9*, but one large-sized band (PS20–PS21) were detected for *Glu-1Bx15* and *Glu-1Bx20*, respectively. Therefore, these primer sets (PS18–21) could distinguish *Glu-1By15* and *Glu-1By20* from other alleles; however, *Glu-1By15* and *Glu-1By20* were detected in the same pattern and could not be distinguished from each other.

### Detection of the Glu-1By16 subunit

Lei et al. [24] previously reported a PCR marker (PS22) for the detection of the *Glu-1By16* allele. However, this marker showed multiple bands (three bands for *Glu-1Bx16*, zero or one band for *Glu-1Bx15* and *Glu-1Bx20*, and two bands for the other *Glu-1By* alleles) (Fig. 5h). Therefore, we developed a *Glu-1By16*-specific PCR-based marker with a product size of 558 bp (PS23, Fig. 5i). This primer set specifically detected the *Glu-1By16* allele in three cultivars: Jeokjoong, Baekjoong, and Joeun.

### Analysis of HMW-GS protein using UPLC

**Fig. 6:**
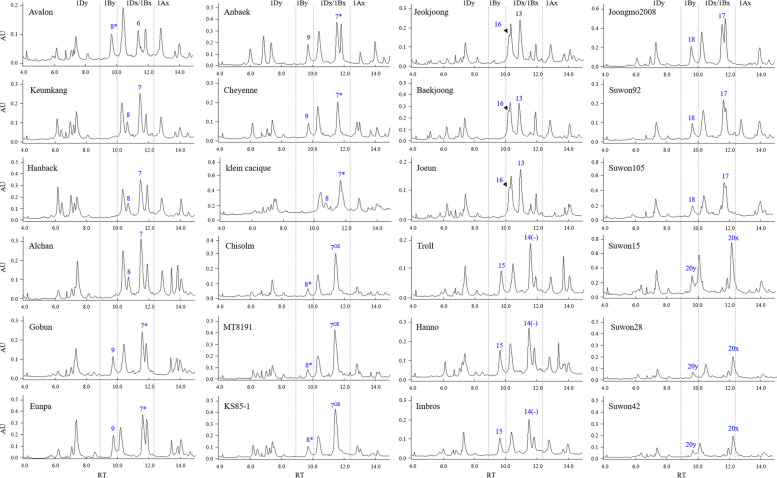
Identification of HMW-GS Glu-1Bx and Glu-1By in 24 wheat cultivars by ultra- performance liquid chromatography. Glu-1Bx and Glu-1By alleles are shown in blue. AU, arbitrary units; RT, retention time

HMW-GS glutenin subunits in wheat grains were confirmed by UPLC analysis (Fig. 6). The retention times of Glu-1Bx7, Glu-1Bx7*, and Glu-1Bx17 were 11.448 ± 0.027, 11.618 ± 0.067, and 11.670 ± 0.074 min, respectively, making it difficult to distinguish them. The retention times of Glu-1By8, Glu-1By8*, and Glu-1By18 were 10. 649 ± 0.022, 9.714 ± 0.048, and 9.676 ± 0.044 min, respectively. Glu-1By8* and Glu-1By18 were extracted earlier than Glu-1Dx, while Glu-1By8 was extracted later than Glu-1Dx. Thus, distinguishing Glu-1Bx8 from Glu-1Bx8* and Glu-1Bx18 may be possible using UPLC; however, it is difficult to distinguish Glu-1By8* and Glu-1By18 from each other. In addition, the extraction times of Glu-1Bx14^(−)^, Glu-1Bx14^(+)^, and Glu-1Bx20 were 11.549 ± 0.023, 12.319 ± 0.105, and 12.173 ± 0.029 min, respectively. The extraction time of Glu-1Bx14^(−)^ was shorter than that of Glu-1Bx14^(+)^ and Glu-1By20, but the extraction times of Glu-1Bx14^(+)^ and Glu-1By20 were very similar, making it difficult to distinguish them. Likewise, the extraction times of Glu-1By15 and Glu-1By20 were very similar (9.687 ± 0.027 min and 9.645 ± 0.019 min, respectively). Therefore, the distinction between the allelic combinations of Glu-1Bx14^(+)^ + Glu-1By15 and Glu-1Bx20 + Glu-1By20 is difficult, even using UPLC analysis.

### Analysis of HMW-GS proteins using SDS-PAGE

**Fig. 7:**
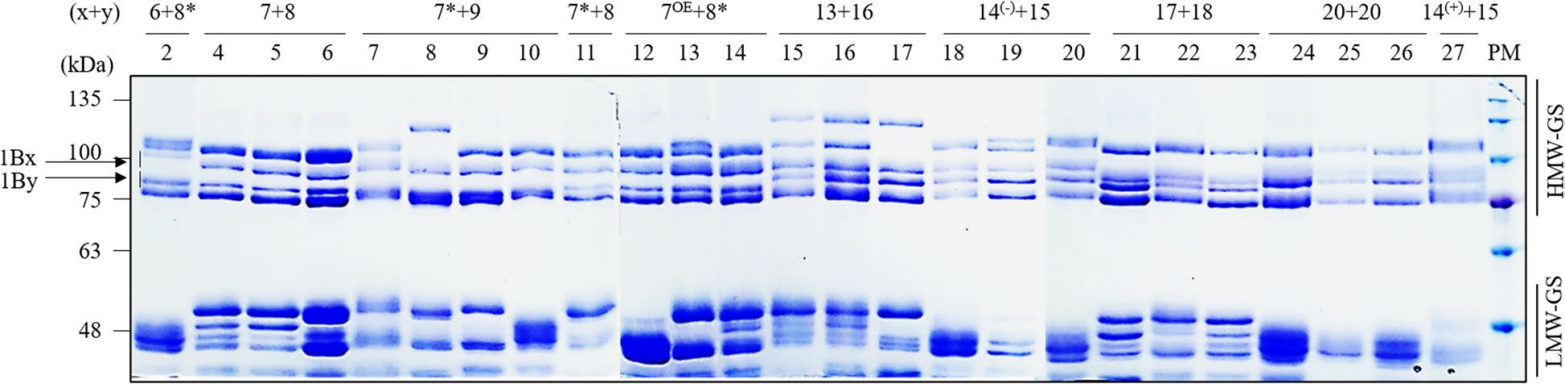
Identification of HMW-GS Glu-1Bx and Glu-1By by SDS-PAGE in 25 wheat cultivars. The numbers above the figure are the same as the variety numbers in Table 1. x + y, Glu-1Bx + Glu-1By; HMW-GS, high-molecular-weight glutenin subunits; LMW-GS, low-molecular-weight glutenin subunits; PM, protein size marker. Arrows indicate Glu-1Bx and Glu-1By alleles

HMW-GS glutenin subunits were compared using SDS-PAGE to confirm the presence of PCR markers in the wheat grains (Fig. 7). Glu-1Bx7 and Glu-1Bx7* differed by five amino acids and showed calculated molecular weights of 85.31 kDa and 84.71 kDa (www.bioinformatics.org), respectively, and the two proteins could not be distinguished by SDS-PAGE. Similarly, the molecular weights of Glu-1Bx14^(−)^, Glu-1Bx14^(+)^, and Glu-1Bx20 were calculated to be 84.53 kDa, 86.23 kDa, and 86.11 kDa, respectively, which were distinct from those of Glu-1Bx14^(−)^ and Glu-1Bx20. However, Glu-1Bx14^(+)^ and Glu-1Bx20 could not be distinguished by SDS-PAGE. Additionally, the molecular weights of Glu-1By8 and Glu-1By18 were calculated to be 77.38 kDa and 77.44 kDa, respectively. Glu-1By8 and Glu-1By8* proteins were highly identical in size and could not be distinguished by SDS-PAGE. The molecular weights of Glu-1By15 and Glu-1By20 were calculated to be 77.40 kDa and 77.43 kDa, respectively, and were also not distinguishable from each other.

## Discussion

Allele variations in HMW-GSs are highly related to wheat baking quality, and among the three *Glu-1* loci, *Glu-B1* shows the greatest allele variations in both tetraploid and hexaploid wheat [50]. Therefore, various mass spectrometry techniques, SDS-PAGE analyses, and molecular markers have been developed to identify HMW-GSs. However, new genotypes continue to be reported, and there are few genotypes that can be distinguished using the developed markers. In addition, many previously reported PCR-based markers often performed simple relative comparison analyses without comparing various alleles. Moreover, the primer sequences and accession numbers were incorrect in some reports [35, 39]. In addition, in some cases, in genotypes of alleles that could not be distinguished by SDS-PAGE and LC analyses were re-evaluated [43, 47].

In this study, 11 novel *Glu-B1* allele identification markers were developed, and together with previously reported markers, they could be used to distinguish nine *Glu-1Bx* alleles (*Glu-1Bx6*, *Glu-1Bx*7, Glu-1Bx7*, *Glu-1Bx7* ^OE^, *Glu-1Bx13*, *Glu-1Bx14* ^(−)^, *Glu-1Bx17*, and *Glu-1Bx14* ^(+)^/20) and seven *Glu-1By* alleles (Glu-1By8, Glu-1By8*, *Glu-1By9*, *Glu-1Bx16*, *Glu-1By18*, and *Glu-1By15/20*). These findings confirmed that the *Glu-1By* allele of Avalone was Glu-1By8*, not *Glu-1By8*, and the *Glu-1Bx* allele of the three cultivars, Gobun, Eunpa, and Anbaek, was confirmed to be Glu-1Bx7*, not *Glu-1By7*. Geng et al. (2014) reported three Chinese and 11 European cultivars among 505 Chinese and 160 European cultivars with a 43 bp insertion. Cultivars containing 43 bp insertion were rare and had a high proportion of the *Glu*-*1Bx7* gene. Among them, *Glu*-*1Bx6* containing 43 bp insertion was identified in European cultivars ‘GK Bence’ and ‘Komorowska-pol’, and *Glu*-*1Bx14* containing 43 bp insertion was found in European cultivars ‘Funo’ and ‘Amarelo de barba branca’ [40]. The present study showed that the *Glu*-*1Bx6* allele from Avalon and the *Glu*-*1Bx14* allele from Troll, Hanno, and Imbros are cultivars that do not contain 43 bp insertion in the promoter. Therefore, two types of promoters possibly exist in the *Glu*-*1Bx6* and *Glu*-*1Bx14* alleles. Cases with and without 43 bp insertions were identified; however, *Glu*-*1Bx6* and *Glu*-*1Bx14* with a 43 bp insertion are rare. Additionally, two *Glu-1Bx14* allele accession numbers have been registered with NCBI and were distinguishable by 18 bp indels. Three cultivars—Hanno, Imbros, and Troll, showed a 18 bp deletion in *Glu*-*1Bx14* in comparison with the other *Glu-1Bx* alleles, but RNAEE did not, allowing the distinction of these cultivars using the markers we developed.

In this study, two DNA polymerases were used for amplification. Unlike the conditions described in the previous studies, the annealing temperature for the optimal conditions differed depending on the DNA polymerase. Therefore, when analyzing markers, the annealing temperature must be set according to the DNA polymerase and equipment.

Glu-1Bx14^(+)^ and Glu-1By20, and Glu-1By15 and Glu-1By20 could not be distinguished using UPLC analysis. Additionally, it was not easy to distinguish the sizes of the two proteins in SDS-PAGE analysis. These findings highlight the need to develop PCR-based markers that can easily distinguish between these two allele combinations. However, we were could not distinguish *Glu-1Bx14* ^(+)^ from *Glu-1Bx20*, nor could it distinguish *Glu-1By15* from *Glu-1By20* with the PCR-based marker. In addition, since many alleles were not tested in this study and more alleles may occur, the primers developed here may not be fully applicable. However, the most commonly used allele combinations can be distinguished by PCR-based markers developed in this study. Additionally, these results suggesting that the *Glu*-*A1* and *Glu*-*D1* alleles also need to be reassessed through PCR-based markers.

## Conclusions

HMW-GS allele composition is a crucial factor in determining end-use quality, and allele identification is an essential task in wheat breeding programs. Seven *Glu*-*1Bx* and four *Glu*-*1By* allele detection markers were developed to detect nine *Glu*-*1Bx* and seven *Glu*-*1By* locus alleles, respectively. The discrimination of *Glu*-*B1* locus alleles can be improved and the most commonly used allele combinations can be identified by integrating previously reported markers and 11 newly developed PCR markers. However, these PCR markers cannot distinguish the *Glu*-*1Bx14* ^(+)^ + *Glu*-*1By15* and *Glu*-*1Bx20* + *Glu*-*1By20* combination; therefore, further research is needed. The developed markers can facilitate more effective analysis of molecular variations in the *Glu*-*B1* allele, thereby improving the end-use quality of common wheat.

### Supplementary Information

Supplementary file 1.

## Data Availability

Data is provided within the manuscript and supplementary file. All raw data are provided in the supplementary file (Fig. S5–S8).

## Abbreviations

HMW-GS: High-molecular-weight glutenin subunit
Indels: Insertions and deletions
LMW-GS: Low-molecular-weight glutenin subunit
SNP: Single-nucleotide polymorphism
PCR: Polymerase chain reaction
SDS-PAGE: Sodium dodecyl sulfate–polyacrylamide gel electrophoresis
UPLC: Ultra-performance liquid chromatography
PS: Primer set

## References

[1] Brankovic G, Dodig D, Pajic V, Kandic V, Knezevic D, Duric N, Zivanovic T (2018). Genetic parameters of Triticum aestivum and Triticum durum for technological quality properties in Serbia. Zemdirbyste-Agriculture.

[2] Gilbert SM, Wellner N, Belton PS, Greenfield JA, Siligardi G, Shewry PR, Tatham AS (2000). Expression and characterisation of a highly repetitive peptide derived from a wheat seed storage protein. Biochim Biophys Acta.

[3] Shewry PR, Halford NG, Tatham AS (1992). High molecular weight subunits of wheat Glutenin. J Cereal Sci.

[4] Payne PI, Law CN, Mudd EE (1988). Control by homoeologous group 1 chromosomes of the high-molecular-weight subunits of glutenin, a major protein of wheat endosperm. Theor Appl Genet.

[5] Gianibelli MC, Gupta RB, Lafiandra D, Margiotta B, MacRitchie F (2001). Polymorphism of high Mr glutenin subunits in Triticum tauschii: characterisation by chromatography and electrophoretic methods. J Cereal Sci.

[6] Gupta RB, Bekes F, Wrigley CK (1991). Prediction of physical dough properties from glutenin subunit composition in bread wheats: correlation studies. Cereal Chem.

[7] Tatham AS, Shewry PR, Miflin BJ (1984). Wheat gluten elasticity: a similar molecular basis to elastin?. FEBS Lett.

[8] Tatham A, Marsh M, Wieser H, Shewry P (1990). Conformational studies of peptides corresponding to the coeliac-activating regions of wheat α-gliadin. Biochem J.

[9] Wieser H (2007). Chemistry of gluten proteins. Food Microbiol.

[10] Branlard G, Dardevet M (1985). Diversity of grain protein and bread wheat quality. II. Correlation between high molecular weight subunits of glutenin and flour quality characteristics. J Cereal Sci..

[11] He ZH, Liu L, Xia XC, Liu JJ, Pena RJ (2005). Composition of HMW and LMW glutenin subunits and their effects on dough properties, pan bread, and noodle quality of Chinese bread wheats. Cereal Chem.

[12] Ng PKW, Bushuk W (1988). Statistical relationships between high molecular weight subunits of glutenin and breadmaking quality of Canadian-grown wheats. Cereal Chem.

[13] Payne PI, Holt LM, Krattiger AF, Carrillo JM (1988). Relationships between seed quality characteristics and HMW glutenin subunit composition determined using wheats grown in Spain. J Cereal Sci.

[14] Shewry PR, Halford NG, Tatham AS, Popineau Y, Lafiandra D, Belton PS (2003). The high molecular weight subunits of wheat glutenin and their role in determining wheat processing properties. Adv Food Nutr Res.

[15] Wang S, Yu Z, Cao M, Shen X, Li N, Li X, Ma W, Weißgerber H, Zeller F, Hsam S, Yan Y (2013). Molecular mechanisms of HMW glutenin subunits from 1s^l^ genome of *Aegilops longissima* positively affecting wheat breadmaking quality. PLoS ONE.

[16] Payne PI, Lawrence GJ (1983). Catalogue of alleles for the complex gene loci, Glu-A1, , and Glu-D1 which code for high-molecular-weight subunits of glutenin in hexaploid wheat. Cereal Res Commun.

[17] Wan Y, Liu K, Wang D, Shewry PR (2000). High-molecular-weight glutenin subunits in the Cylindropyrum and Vertebrata section of the Aegilops genus and identification of subunits related to those encoded by the Dx alleles of common wheat. Theor Appl Genet.

[18] Roy N, Islam S, Ma J, Lu M, Torok K, Tomoskozi S, Bekes F, Lafiandra D, Appels R, Ma W (2018). Expressed Ay HMW glutenin subunit in Australian wheat cultivars indicates a positive effect on wheat quality. J Cereal Sci.

[19] Bietz JA, Shepherd KW, Wall JS (1975). Single-kernel analysis of glutenin: use in wheat genetics and breeding. Cereal Chem.

[20] Yan Y, Hsam SLK, Yu J, Jiang Y, Zeller FJ (2003). Allelic variation of the HMW glutenin subunits in *Aegilops tauschii* accessions detected by sodium dodecyl sulphate (SDS-PAGE), acid polyacrylamide gel (A-PAGE) and capillary electrophoresis. Euphytica.

[21] Gao L, Ma W, Chen J, Wang K, Li J, Wang S, Yan Y (2010). Characterization and comparative analysis of wheat high molecular weight glutenin subunits by SDS-PAGE, RP-HPLC, HPCE, and MALDI-TOF-MS. J Agric Food Chem.

[22] Yu Z, Han C, Yan X, Li X, Jiang G, Yan Y (2013). Rapid characterization of wheat low molecular weight glutenin subunits by ultraperformance liquid chromatography (UPLC). J Agric Food chem.

[23] Butow BJ, Ma W, Gale KR, Cornish GB, Rampling L, Larroque O, Morell MK, Bekes F (2003). Molecular discrimination of Bx7 alleles demonstrates that a highly expressed high-molecular-weight glutenin allele has a major impact on wheat flour dough strength. Theor Appl Genet.

[24] Lei ZS, Gale KR, He ZH, Gianibelli C, Larroque O, Xia XC, Butow BJ, Ma W (2006). Y-type gene specific markers for enhanced discrimination of high molecular weight glutenin alleles at the *Glu-B1* locus in hexaploid wheat. J Cereal Sci.

[25] Dong K, Hao C, Wang A, Cai M, Yan Y (2009). Characterization of HMW glutenin subunits in bread and tetraploid wheats by reversed-phase high-performance liquid chromatography. Cereal Res Commun.

[26] Marchylo BA, Lukow OM, Kruger EJ (1992). Quantitative variation in high molecular weight glutenin subunit 7 in some Canadian wheats. J Cereal Sci.

[27] Smith RL, Schweder ME, Barnett RD (1994). Identification of glutenin alleles in wheat and triticale using PCR-generated DNA markers. Crop Sci.

[28] Ma W, Zhang W, Gale KR (2003). Multiplex-PCR typing of high molecular weight glutenin alleles in wheat. Euphytica.

[29] Liu S, Chao S, Anderson JA (2008). New DNA markers for high molecular weight glutenin subunits in wheat. Theor Appl Genet.

[30] Ahmad M (2000). Molecular marker-assisted selection of HMW glutenin alleles related to wheat bread quality by PCR-generated DNA markers. Theor Appl Genet.

[31] Xu Q, Xu J, Liu CL, Chang C, Wang CP, You MS, Li BY, Liu GT (2008). PCR based markers for identification of HMWGS at *Glu-B1x* loci in common wheat. J Cereal Sci.

[32] Schwarz G, Felsenstein FG, Wenzel G (2004). Development and validation of a PCR-based marker assay for negative selection of the HMW glutenin allele GluB1-1d (Bx-6) in wheat. Theor Appl Genet.

[33] Ragupathy R, Naeem HA, Reimer E, Lukow OM, Sapirstein HD, Cloutier S (2008). Evolutionary origin of the segmental duplication encompassing the wheat *Glu-B1* locus encoding the overexpressed Bx7 (Bx7^OE^) high molecular weight glutenin subunit. Theor Appl Genet.

[34] Liang X, Zhen S, Han C, Wang C, Li X, Ma W, Yan Y (2015). Molecular characterization and marker development for hexaploid wheat-specific HMW glutenin subunit 1By18 gene. Mol Breed.

[35] Espi A, Giraldo P, Rodriguez-Quijano M, Carrillo JM (2012). A PCR-based method for discriminating between high molecular weight glutenin subunits Bx7 and Bx7* in *Triticum aestivum* L. Plant Breeding.

[36] Rai R, Singh S, Das BK, Bhagwat SG (2018). Application of allele-specific (AS-PCR) marker for identification of high-molecular-weight glutenin subunits (HMW-GS) at the GluB-1 locus in bread wheat (Triticum aestivum L.). Adv Crop Sci Tech..

[37] Frank K, Miro K, Nagy T, Marincs F (2017). Development of a PCR-based DNA marker for *Glu-1By* alleles in the old Hungarian Bánkúti wheat. Mol Breed.

[38] Wei L, Bai SG, Hou XJ, Li JM, Zhang B, Chen WJ, Liu DC, Liu BL, Zhang HG (2014). A new HMW-GS *1Bx23** containing an amino acid segment similar to collagen. Cereal Res Commun.

[39] Li W, Wan Y, Liu Z, Liu K, Liu X, Li B, Li Z, Zhang X, Wang D (2004). Molecular characterization of HMW glutenin subunit allele 1Bx14: further insights into the evolution of Glu-B1-1 alleles in wheat and related species. Theor Appl Genet.

[40] Geng Y, Pang B, Hao C, Tang S, Zhang X, Li T (2014). Expression of wheat high molecular weight glutenin subunit 1Bx is affected by large insertions and deletions located in the upstream flanking sequences. PLoS ONE.

[41] Tamura K, Stecher G, Kumar S (2021). MEGA11: molecular evolutionary genetics analysis version 11. Mol Biol Evol.

[42] Zhang Q, Dong YM, An XL, Wang A, Zhang YZ, Li XH, Gao LY, Xia XC, He ZH, Yan YM (2008). Characterization of HMW glutenin subunits in common wheat and related species by matrix-assisted laser desorption/ionization time-of-flight mass spectrometry (MALDI-TOF-MS). J Cereal Sci.

[43] Lee MH, Choi C, Kim KH, Son JH, Lee GE, Choi JY, Kang CS, Sohn J, Ko JM, Kim KM (2023). Generation of wheat near-isogenic lines overexpressing 1Bx7 glutenin with increased protein contents and SDS-sedimentation values. Plants.

[44] D’Ovidio R, Porceddu E, Lafiandra D (1994). PCR analysis of genes encoding allelic variants of high-molecular-weight glutenin subunits at the Glu-D1 locus. Theor Appl Genet..

[45] Yang ZJ, Li GR, Liu C, Feng J, Zhou JP, Ren ZL (2006). Molecular characterization of a HMW glutenin subunit allele providing evidence for silencing of x-type gene on *Glu-B1*. Acta Genet Sin.

[46] Radovanovic N, Cloutier S (2003). Gene-assisted selection for high molecular weight glutenin subunits in wheat doubled haploid breeding programs. Mol Breed.

[47] Jang YR, Beom HR, Altenbach SB, Lee MK, Lim SH, Lee JY (2017). Improved method for reliable HMW-GS identification by RP-HPLC and SDS-PAGE in common wheat cultivars. Molecules.

[48] Park CS, Kang CS, Jeung JU, Woo SH (2011). Influence of allelic variations in glutenin on the quality of pan bread and white salted noodles made from Korean wheat cultivars. Euphytica.

[49] Anderson OD, Greene FC (1989). The characterization and comparative analysis of high-molecular-weight glutenin genes from genomes A and B of a hexaploid bread wheat. Theor Appl Genet.

[50] Gianibelli MC, Larroque OR, MacRitchie F, Wrigley CW (2001). Biochemical, genetic, and molecular characterization of wheat endosperm proteins. Cereal Chem.

