# Supplementary material for: Development of PCR-based markers for identification of wheat HMW glutenin Glu-1Bx and Glu-1By alleles
Source: BMC Plant Biol. 2024 May 14;24:395. doi: 10.1186/s12870-024-05100-w (PMC11092038; doi:10.1186/s12870-024-05100-w)
Supplement: Supplementary file 1 [file 12870_2024_5100_MOESM1_ESM.docx]

Supplementary information

**Development of PCR-based markers for identification of wheat HMW glutenin *Glu*-*1Bx* and *Glu*-*1By* alleles**

Myoung Hui Lee, Kyeong-Min Kim, Chon-Sik Kang, Mira Yoon, Ki-Chang Jang, and Changhyun Choi*

National Institute of Crop Science, Rural Development Administration, Wanju, 55365, Korea

*Corresponding author: [chchhy@korea.kr](mailto:chchhy@korea.kr); Tel.: +82-63-238-5454

**Fig. S1.** Alignment of the sequences of *Glu-1Bx* alleles and primer positions.

PS1-F/PS9-F/PS10-F

Bx6 **ATGGCTAAGCGCC**TGGTCCTCTTTGCGGCAGTAGTCGTCGCCCTCGTGGCTCTCACCGCC 60

Bx7 ATGGCTAAGCGCCTGGTCCTCTTTGCGGCAGTAGTCGTCGCCCTCGTGGCTCTCACCGCC 60

Bx7* ATGGCTAAGCGCCTGGTCCTCTTTGCGGCAGTAGTCGTCGCCCTCGTGGCTCTCACCGCC 60

Bx13 ATGGCTAAGCGCCTGGTCCTCTTTGCGGCAGTAGTCGTCGCCCTCGTGGCTCTCACCGCC 60

Bx14(+) ATGGCTAAGCGCCTGGTCCTCTTTGCGGCAGTAGTCGTCGCCCTCATGGCTCTCACCGCC 60

Bx14(-) ATGGCTAAGCGGTTGGTCCTCTTTGCGGCAGTAGTCGTCGCCCTCGTGGCTCTCACCGCC 60

Bx14* ATGGCTAAGCGCCTGGTCCTCTTTGCGGCAGTAGTCGTCGCCCTCGTGGCTCTCACCGCC 60

Bx17 ATGACTAAGCGCCTGGTCCTCTTTGCGGCAGTAGTCGTCGCCCTCGTGGCTCTCACCGCC 60

Bx20 ATGGCTAAGCGCCTGGTCCTCTTTGCGGCAGTAGTCGTCGCCCTCATGGCTCTCACCGCC 60

Bx23 ATGGCTAAGCGCCTGGTCCTCTTTGCGGCAGTAGTCGTCGCCCTCGTGGCTCTCACCGCC 60

Bx23* ATGGCTAAGCGCCTGGTCCTCTTTGCGGCAGTAGTCGTCGCCCTCGTGGCTCTCACCGCC 60

*** ******* ******************************** **************

Bx6 GCTGAAGGTGAGGCCTCTGGACAACTACAATGTGAGCGCGAGCTCCGGAAGCGCGAGCTC 120

Bx7 GCTGAAGGTGAGGCCTCTGGACAACTACAATGTGAGCACGAG---------------CTC 105

Bx7* GCTGAAGGTGAGGCCTCTGGACAACTACAATGTGAGCACGAG---------------CTC 105

Bx13 GCTGAAGGTGAGGCCTCTGGACAACTACAATGTGAGCGCGAG---------------CTC 105

Bx14(+) GCTGAAGGTGAGGCCTCTGGACAACTACAATGTGAGCGCGAGCTCCGGAAGCGCGAGCTC 120

Bx14(-) GCTGAAGGTGAGGCCTCTGGACAACTACAATGTGAGCGCGAG---------------CTC 105

Bx14* GCTGAAGGTGAGGCCTCCGGACAACTACAATGTGAGCGCGAG---------------CTC 105

Bx17 GCTGAAGGTGAGGCCTCTGGACAACTACAATGTGAGCACGAG---------------CTC 105

Bx20 GCTGAAGGTGAGGCCTCTGGACAACTACAATGTGAGCGCGAGCTCCGGAAGCGCGAGCTC 120

Bx23 GCTGAAGGTGAGGCCTCCGGACAACTACAATGTGAGCGCGAG---------------CTC 105

Bx23* GCTGAAGGTGAGGCCTCCGGACAACTACAATGTGAGCGCGAG---------------CTC 105

***************** ******************* **** ***

Bx6 GAGGCATGCCAACAGGTGGTGGACCAGCAACTCCGAGACGTTAGCCCCGGGTGCCGCCCC 180

Bx7 GAGGCATGCCAACAGGTGGTGGACCAGCAACTCCGAGACGTTAGCCCCGGGTGCCGCCCC 165

Bx7* GAGGCATGCCAACAGGTGGTGGACCAGCAACTCCGAGACGTTAGCCCCGGGTGCCGCCCC 165

Bx13 GAGGCATGCCAACAGGTGGTGGACCAGCAACTTCGAGACGTTAGCCCCGGGTGCCGCCCC 165

Bx14(+) GAGGCATACCAACAGGTGGTGGACCAGCAACTCCGAGACGTTAGCCCCGGGTACCGCCCC 180

Bx14(-) GAGGCATGCCAACAGGTGGTGGACCAGCAACTCCGAGACGTTAGCCCCGGGTGCCGCCCC 165

Bx14* GAGGCATGCCAACAGGTGGTGGACCAGCAACTCCGAGACGTTAGCCCCGGGTGCCGCCCC 165

Bx17 GAGGCATGCCAACAGGTGGTGGACCAGCAACTCCGAGACGTTAGCCCCGGGTGCCGCCCC 165

Bx20 GAGGCATACCAACAGGTGGTGGACCAGCAACTCCGAGACGTTAGCCCCGGGTACCGCCCC 180

Bx23 GAGGCATGCCAACAGGTGGTGGACCAGCAACTCCGAGACGTTAGCCCCGGGTGCCGCCCC 165

Bx23* GAGGCATGCCAACAGGTGGTGGACCAGCAACTCCGAGACGTTAGCCCCGGGTGCCGCCCC 165

******* ************************ ******************* *******

PS12-F PS9-R

Bx6 ATCACCGTCAGCCCGGGCACGAGGCAATACGAGCAGCAA**CCTGTGGTGCCGTCCAAGGC**C 240

Bx7 ATCACCGTCAGCCCGGGCACGAGACAATACGAGCAGCAACCTGTGGTGCCGTCCAAGGCC 225

Bx7* ATCACCGTCAGCCCGGGCACGAGACAATACGAGCAGCAACCTGTGGTGCCGTCCAAGGCC 225

Bx13 ATCACCGTCAGCCCG**GGCACGAGGCAATACGAGCG**GCAACCTGTGGTGCCGTCCAAGGCC 225

Bx14(+) ATCACCGTCAGCCCGGGCACGAGACAATACGAGCAGCAACCTGTGGTGCCGCCCAAGGCC 240

Bx14(-) ATCACCGTCAGCCCGGGCACGAGACAATACGAGCAGCAACCTGTGGTGCCGTCCAAGGCC 225

Bx14* ATCACCGTCAGCCCGGGCACGAGACAATACGAGCAGCAACCTGTGATGCCGTCCAAGGCC 225

Bx17 ATCACCGTCAGCCCGGGCACGAGACAATACGAGCAGCAACCTGTGGTTGCGTCCAAGGCC 225

Bx20 ATCACCGTCAGCCCGGGCACGAGACAATACGAGCAGCAACCTGTGGTGCCGTCCAAGGCC 240

Bx23 ATCACCGTCAGCCCGGGCACGAGACAATACGAGCAGCAACCTGTGATGCCGTCCAAGGCC 225

Bx23* ATCACCGTCAGCCCGGGCACGAGACAATACGAGCAGCAACCTGTGATGCCGTCCAAGGCC 225

*********************** ********** ********** * ** ********

Bx6 GGATCCTTCTACCCCAGCGAGACTACGCCTTCGCAGCAACTCCAACAAATGATATTTTGG 300

Bx7 GGATCCTTCTACCCCAGCGAGACTACGCCTTCGCAGCAACTCCAACAAATGATATTTTGG 285

Bx7* GGATCCTTCTACCCCAGCGAGACTACGCCTTCGCAGCAACTCCAACAAATGATATTTTGG 285

Bx13 GGATCCTTCTACCCCAGCAAGACTACGCCTTCGCAGCAACTCCAACAAATGATATTTTGG 285

Bx14(+) GGATCCTTCTACCCCAGCGAGACTACGCCTTCGCAGCAACTCCAACAAATGATATTTTGG 300

Bx14(-) GGATCCTTCTACCCCAGCGAGACTACGCCTTCGCAGCAACTCCAACAAATGATATTTTGG 285

Bx14* GGATCCTTCTACCCCAGCGAGACTACGCCTTCGCAGCAACTCCAACAAATGATATTTTGG 285

Bx17 GGATCCTTCTACCCCAGCGAGACTACGCCTTCGCAGCAACTCCAACAAATGATATTTTGG 285

Bx20 GGATCCTTCTACCCCAGCGAGACTACGCCTTCGCAGCAACTCCAACAAATGATATTCTGG 300

Bx23 GGATCCTTCTACCCCAGCGAGACTACGCCTTCGCAGCAACTCCAACAAATGATATTTTGG 285

Bx23* GGATCCTTCTACCCCAGCGAGACTACGCCTTCGCAGCAACTCCAACAAATGATATTTTGG 285

****************** ************************************* ***

Bx6 GGAATACCTGCACTACTAAGAAGGTATTACCCAAGTGTAACTTCTTCGCAGCAGGGGTCA 360

Bx7 GGAATACCTGCACTACTAAGAAGGTATTACCCAAGTGTAACTTCTTCGCAGCAGGGGTCA 345

Bx7* GGAATACCTGCACTACTAAGAAGGTATTACCCAAGTGTAACTTCTTCGCAGCAGGGGTCA 345

Bx13 GGAATACCTGCACTACTAAGAAGGTATTACCCAAGTGTAACTTCTTCGCAGCAGGGGTCA 345

Bx14(+) GGAATACCTGCACTACTAAGAAGGTATTACCCAAGTGTAACTTCTTCGCAGCAGGGGTCA 360

Bx14(-) GGAATACCTGCACTACTAAGAAGGTATTACCCAAGTGTAACTTCTTCGCAGCAGGGGTCA 345

Bx14* GGAATACCTGCACTACTAAGAAGGTATTACCCAAGTGTAACTTCTTCGCAGCAGGGGTCA 345

Bx17 GGAATACCTGCACTACTAAGAAGGTATTACCCAAGTGTAACTTCTTCGCAGCAGGGGTCA 345

Bx20 GGAATACCTGCACTACTAAGAAGGTATTACCCAAGTGTAACTTCTTCGCAGCAGGGGTCA 360

Bx23 GGAATACCTGCACTACTAAGAAGGTATTACCCAAGTGTAACTTCTTCGCAGCAGGGGTCA 345

Bx23* GGAATACCTGCACTACTAAGAAGGTATTACCCAAGTGTAACTTCTTCGCAGCAGGGGTCA 345

************************************************************

Bx6 TACTATCCAGGCCAAGCTTCTCAGCAACAATCAGGACAAGGACAGCAGCCAGGACAAGGA 420

Bx7 TACTATCCAGGCCAAGCTTCTCCCCAACAGTCAGGACAAGGACAGCAGCCAGGACAAGAA 405

Bx7* TACTATCCAGGCCAAGCTTCTCCCCAACAGTCAGGACAAGGACAGCAGCCAGGACAAGAA 405

Bx13 TACTATCCAGGCCAAGCTTCTCCGCAACAGTTAGGACAAGGACAGCAGCCAGGACAAGGA 405

Bx14(+) TACTATCCAGGCCAAGCTTTTCCGCAACAATCAGGACAAGGACAGCAGCCAGGACAAGGA 420

Bx14(-) TACTATCCAGGCCAAGCTTCTCCGCAACAGTCAGGACAAGGACAGCAGCCAGGACAAGGA 405

Bx14* TACTATCCAGGCCAAGCTTCTCCGCAACAGTCAGGACAAGGACAGCAGCCAGGACAAGGA 405

Bx17 TACTATCCAGGCCAAGCTTCTCCCCAACAGTCAGGACAAGGACAGCAGCCAGGACAAGAA 405

Bx20 TACTATCCAGGCCAAGCTTTTCCGCAACAATCAGGACAAGGACAGCAGCCAGGACAAGGA 420

Bx23 TACTATCCAGGCCAAGCTTCTCCGCAACAGTCAGGACAAGGACAGCAGCCAGGACAAGGA 405

Bx23* TACTATCCAGGCCAAGCTTCTCCGCAACAGTCAGGACAAGGACAGCAGCCAGGACAAGGA 405

******************* ** ***** * ************************** *

PS1-R/PS10-R/PS12-R

Bx6 CAGCAACCA**GAACAAGGGCAACAAGATC**AGCAGCCAGGACAAGGACAACAAGGGTACTAC 480

Bx7 CAGCAACCA**GGACAAGGGCAACAAGATC**AGCAGCCAGGACAAAGACAACAAGGATACTAC 465

Bx7* CAGCAACCAGGACAAGGGCAACAAGATCAGCAGCCAGGACAAAGACAACAAGGATACTAC 465

Bx13 CAGCAACCA**AGACAAGAGCAACAAGATC**AGCAGCCAGGACAAAGACAACAAGGATACTAC 465

Bx14(+) CAGCAACCAGGACAAAGGCAACAAGATCAGCAGCCAGGACAAGGACAACAAGGGTACTAC 480

Bx14(-) CAGCAACCAGGACAAGGGCAACAAGATCAGCAACCAGGACAAAGACAACAAGGATACTAT 465

Bx14* CAGCAACCAGAACAAAGGCAACAAGATCAGCAGCCAGGACAAAGACAACAAGGATACTAC 465

Bx17 CAGCAACCAGGACAAGGGCAACAAGATCAGCAGCCAGGACAAAGACAACAAGGATACTAC 465

Bx20 CAGCAACCAGGACAAAGGCAACAAGATCAGCAGCCAGGACAAGGACAACAAGGGTACTAC 480

Bx23 CAGCAACCAGAACAAAGGCAACAAGATCAGCAGCCAGGACAAAGACAACAAGGATACTAC 465

Bx23* CAGCAACCAGAACAAAGGCAACAAGATCAGCAGCCAGGACAAAGACAACAAGGATACTAC 465

********* **** *************** ********* ********** *****

Bx6 CCAACTTCTCCGCAACAGCCAGGACAAGGGCAACAACTGGGACAAGGGCAACCAGGGTAC 540

Bx7 CCAACTTCTCCGCAACAGCCAGGACAAGGGCAACAACTGGGACAAGGGCAACCAGGGTAC 525

Bx7* CCAACTTCTCCGCAACAGCCAGGACAAGGGCAACAACTGGGACAAGGGCAACCAGGGTAC 525

Bx13 CCAACTTCTCCGCAACAGCCAGGACAAGGGCAACAACTGGGGCAAGGGCAACCAGGGTAC 525

Bx14(+) CCAACTTCTCCGCAACAGCCAGGACAAGGGCAACAACTGGGACAAGGGCAACCAGGGTAC 540

Bx14(-) CCAACTTCTCCGCAACAGCCAGGACAAGGGCAACAATTGGGACAAGGGCAACCAGGGTAC 525

Bx14* CCAACTTCTCCGCAACAGCCAGGACAAGGGCAACAACTGGGACAAGGGCAACCAAGGTAC 525

Bx17 CCAACTTCTCCGCAACAGCCAGGACAAGGGCAACAACTGGGACAAGGGCAACCAGGGTAC 525

Bx20 CCAACTTCTCCGCAACAGCCAGGACAAGGGCAACAACTGGGACAAGGGCAACCAGGGTAC 540

Bx23 CCAACTTCTCCGCAACAGCCAGGACAAGGGCAACAACTGGGACAAGGGCAACCAAGGTAC 525

Bx23* CCAACTTCTCCGCAACAGCCAGGACAAGGGCAACAACTGGGACAAGGGCAACCAAGGTAC 525

************************************ **** ************ *****

Bx6 TACCCAACTTCACAGCAGCCAGGACAAAAGCAGCAGGCAGGACAAGGGCAACAATCAGGA 600

Bx7 TACCCAACTTCACAGCAGCCAGGACAAAAGCAGCAGGCAGGACAAGGGCAACAATCAGGA 585

Bx7* TACCCAACTTCACAGCAGCCAGGACAAAAGCAGCAGGCAGGACAAGGGCAACAATCAGGA 585

Bx13 TACCCAACTTCACAGCAGCCAGGACAAAAGCAGCAGGCAGGACAAGGGCAACAATCAGGA 585

Bx14(+) TACCCAACTTCACAGCAGCCAGGACAAAAGCAGCAGGCAGGACAAGGGCAACAATCAGGA 600

Bx14(-) TACCCAACTTCACAGCAGCCAGGACAAAAGCAGCAGCCAGGACAAGGGCAACAATCAGGA 585

Bx14* TACCCAACTTCACAGCAGCCAGGACAAAAGCAGCAGGCAGGACAAGGGCAACAATCAGGA 585

Bx17 TACCCAACTTCACAGCAGCCAGGACAAAAGCAGCAGGCAGGACAAGGGCAACAATCAGGA 585

Bx20 TACCCAACTTCACAGCAGCCAGGACAAAAGCAGCAGGCAGGACAAGGGCAACAATCAGGA 600

Bx23 TACCCAACTTCACAGCAGCCAGGACAAAAGCAGCAGGCAGGACAAGGGCAACAATCAGGA 585

Bx23* TACCCAACTTCACAGCAGCCAGGACAAAAGCAGCAGGCAGGACAAGGGCAACAATCAGGA 585

************************************ ***********************

Bx6 CAAGGACAACAAGGGTACTACCCAACTTCCCTGCAACAGTCAGGACAAGGGCAACAACCG 660

Bx7 CAAGGACAACAAGGGTACTACCCAACTTCCCCGCAACAGTCAGGACAAGGGCAACAACCG 645

Bx7* CAAGGACAACAAGGGTACTACCCAACTTCCCCGCAACAGTCAGGACAAGGGCAACAACCG 645

Bx13 CAAGGACAACAAGGGTACTACCCAACTTCCCCGCAACAGTCAGGACAAGGGCAACAACCG 645

Bx14(+) CAAGGACAACAAAGGTACTACCCAACTTCCCCGCAACAGTCAGGACAAGGGCAACAACCG 660

Bx14(-) CAAGGACAACAAGGGTACTACCCAACTTCCCCGCAACAGTCAGGACAAGGGCAACAACCG 645

Bx14* CAAGGACAACAAGGGTACTACCCAACTTCCCCGCAACAGTCAGGACAAGGGCAACAACCG 645

Bx17 CAAGGACAACAAGGGTACTACCCAACTTCCCCGCAACAGTCAGGACAAGGGCAACAACCG 645

Bx20 CAAGGACAACAAAGGTACTACCCAACTTCCCCGCAACAGTCAGGACAAGGGCAACAACCG 660

Bx23 CAAGGACAACAAGGGTACTACCCAACTTCCCCGCAACAGTCAGGACAAGGGCAACAACCG 645

Bx23* CAAGGACAACAAGGGTACTACCCAACTTCCCCGCAACAGTCAGGACAAGGGCAACAACCG 645

************ ****************** ****************************

Bx6 GGACAAGGGCAACCAGGGTACTACCCAACTTCTCCGCAGCAGTCAGGACAATGGCATCAA 720

Bx7 GGACAAGGGCAACCAGGGTACTACCCAACTTCTCCGCAGCAGTCAGGACAATGGCAGCAA 705

Bx7* GGACAAGGGCAACCAGGGTACTACCCAACTTCTCCGCAGCAGTCAGGACAATGGCAGCAA 705

Bx13 GGACAAGGGCAAGCAGGGTACTACCCAACTTCTCCGCAGCAGTCAGGACAATGGCAGCAA 705

Bx14(+) GGACAAGGGCAACCAGGGTACTACCCAATTTCTCCGCAGCAGTCAGAACAATGGCAGCAA 720

Bx14(-) GGACAAGGGCAACCAGGGTACTACCCAAGTTCTCCGCAGCAGTCAGGACAATGGCAGCAA 705

Bx14* GGACAAGGGCAATCAAGGTACTACCCAACTTCTCCGCAGCAGTCAGGACAATGGCAGCAA 705

Bx17 GGACAAGGGCAACCAGGGTACTACCCAACTTCTCCGCAGCAGTCAGGACAATGGCAGCAA 705

Bx20 GGACAAGGGCAACCAGGGTACTACCCAATTTCCCCGCAGCAGTCAGAACAATGGCAGCAA 720

Bx23 GGACAAGGGCAATCAAGGTACTACCCAACTTCTCCGCAGCAGTCAGGACAATGGCAGCAA 705

Bx23* GGACAAGGGCAATCAAGGTACTACCCAACTTCTCCGCAGCAGTCAGGACAATGGCAGCAA 705

************ ** ************ *** ************* ********* ***

Bx6 CCAGGACAAGGGCAACAGCCAGGACAAGGGCAGCAATCAGGACAAGGGCAACAAGGGCAG 780

Bx7 CCAGGACAAGGGCAACAACCAGGACAAGGGCAGCAATCAGGACAA--------------- 750

Bx7* CCAGGACAAGGGCAACAACCAGGACAAGGGCAGCAATCAGGACAA--------------- 750

Bx13 CCAGGACAAGGGCAACAGCCAGGACAAGGGCAGCAATCAGGACAA--------------- 750

Bx14(+) CCAGGACAAGGGCAACAGCCAGGACAAGGGCAGCAATCGGGACAA--------------- 765

Bx14(-) CCAGGACAAGGGCAACAGCCAGGACAAGGGCAGCAATCAGGACAA--------------- 750

Bx14* CCAGGACAAGGGCAACAGCCAGGACAAGGGCAGCAATTAGGACAA--------------- 750

Bx17 CCAGGACAAGGGCAACAACCAGGACAAGGGCAGCAATCAGGACAA--------------- 750

Bx20 CCAGGACAAGGGCAACAGCCAGGACAAGGGCAGCAATCAGGACAA--------------- 765

Bx23 CCAGGACAAGGGCAACAGCCAGGACAAGGGCAGCAATTAGGACAA--------------- 750

Bx23* CCAGGACAAGGGCAACAGCCAGGACAAGGGCAGCAATTAGGACAA--------------- 750

***************** ******************* ******

Bx6 CAATCAGGACAAGGGCAACAAGGTCAGCAGCCAGAACAAGGGCAACGACCAGGACAAGGA 840

Bx7 ------------GGGCAACAAGGTCAGCAGCCAGGACAAGGGCAACGACCAGGACAAGGA 798

Bx7* ------------GGGCAACAAGGTCAGCAGCCAGGACAAGGGCAACGACCAGGACAAGGA 798

Bx13 ------------GGGCAACAAGGTCAGCAGCCAGGACAAGGGCAACGACCAGGACAAGGA 798

Bx14(+) ------------GGGCAACAAGGTCAGCAGCCAGGACAAGGGCAACGACCAGGACAAGGA 813

Bx14(-) ------------GGGCAACAAGGTCAGCAGCCAGGACAAGGGCAACGACCGGGACAAGGA 798

Bx14* ------------GGGCAACAAGGTCAGCAACCAGGACAAGGGCAACGACCAGGACAAGGA 798

Bx17 ------------GGGCAACAAGGTCAGCAGCCAGGACAAGGGCAACGACCAGGACAAGGA 798

Bx20 ------------GGGCAACAAGGTCAGCAGCCAGGACAAGGGCAACGACCAGGACAAGGA 813

Bx23 ------------GGGCAACAAGGTCAGCAGCCAGGACAAGGGCAACGACCAGGACAAGGA 798

Bx23* ------------GGGCAACAAGGTCAGCAACCAGGACAAGGGCAACGACCAGGACAAGGA 798

***************** **** *************** *********

Bx6 CAACAAGGGTACTACCCAACTTCTCCGCAACAGCCGGGACAAGGGCAACAATCAGGACAA 900

Bx7 CAACAAGGGTACTACCCAATTTCTCCGCAACAGCCGGGACAAGGGCAACAATCAGGACAA 858

Bx7* CAACAAGGGTACTACCCAATTTCTCCGCAACAGCCGGGACAAGGGCAACAATCAGGACAA 858

Bx13 CAACAAGGGTACTACCCAACTTCTCCGCAACAGCCGGGACAAGGGCAACAATCAGGACAA 858

Bx14(+) CAACAAGGGTACTACCCAACTTCTCTGCAACAGCCGAGACAAGGGCAACAATCAGGACAA 873

Bx14(-) CAACAAGGGTACTACCCAACTTCTTCGCAACAGCCGGGACAAGGGCAACAATCAGGACAA 858

Bx14* CAACAAGGGTACTACCCAACTTCTCCGCAACAGCCGGGACAAGGGCAACAATCAGGACAA 858

Bx17 CAACAAGGGTACTACCCAATTTCTCCACAACAGCCGGGACAAGGGCAACAATCAGGACAA 858

Bx20 CAACAAGGGTACTACCCAACTTCTCTGCAACAGCCGGGACAAGGGCAACAATCAGGACAA 873

Bx23 CAACAAGGGTACTACCCAACTTCTCCGCAACAGCCGGGACAAGGGCAACAATCAGGACAA 858

Bx23* CAACAAGGGTACTACCCAACTTCTCCGCAACAGCCGGGACAAGGGCAACAATCAGGACAA 858

******************* **** ********* ***********************

Bx6 GGGCAACCAGGGTACTACCCAACTTCTTCGCGGCAGCCAGGACAA--TGGCAGCAACCAG 958

Bx7 GGGCAACCAGGGTACTACCCAACTTCTTTGCGGCAGCCAGGACAA--TGGCAGCAACCAG 916

Bx7* GGGCAACCAGGGTACTACCCAACTTCTTTGCGGCAGCCAGGACAA--TGGCAGCAACCAG 916

Bx13 GGGCAACCAGGGTACTACCCAACTTCTTTGCGGCAGCCAGGACAA--TGGCAGCAACCAG 916

Bx14(+) GGGCAACCAGGGTACTACCCAACTTCTTCGCGGCAACCAGGACAA--TGGCAGCAACCAG 931

Bx14(-) GGGCAACCAGGGTACTACCCAACTTCTTTGCGGCAGCCAGGACAA--TGGCAGCAACCAG 916

Bx14* GGGCAACCAGGGTACTACCCAACTTCTTTGCGGCAGCCAGGACAA--TGGCAGCAACCAG 916

Bx17 GGGCAACCAGGTTACTACCCAACTTCTTTTGCGCAGCCCAGGACAATGGCAGCAACCCAG 918

Bx20 GGGCAACCAGGGTACTACCCAACTTCTTCGCGGCAACCAGGACAA--TGGCAGCAACCAG 931

Bx23 GGGCAACCAGGGTACTACCCAACTTCTTTGCGGCAGCCAGGACAA--TGGCAGCAACCAG 916

Bx23* GGGCAACCAGGGTACTACCCAACTTCTTTGCGGCAGCCAGGACAA--TGGCAGCAACCAG 916

*********** **************** *** ** * * * * ****

Bx6 GACAAGGACAGCAACCAGGACAAGGGCAACAAGGTCAGCAGCCAGGACAAGGACAACAAC 1018

Bx7 GACAAGGGCAGCAACCAGGACAAGGGCAACAAGGTCAGCAGCCAGGACAAGGACAACAAT 976

Bx7* GACAAGGGCAGCAACCAGGACAAGGGCAACAAGGTCAGCAGCCAGGACAAGGACAACAAT 976

Bx13 GACAAGGGCAGCAACCAGGACAAGGGCAACAAGGTCAGCAGCCAGGACAAGGACAACAAC 976

Bx14(+) GACAAGGGCAGCAACCAGGACAAGGGCAACAAGGTCAGCAGCCAGGACAAGGACAACAAC 991

Bx14(-) GACAAGGGCAGCAATCAGGACAAGGGCAACAAGGTCAGCAGCCAGGACAAGGACAACAAC 976

Bx14* GACAAGGGCAGCAACCAGGACAAGGGCAACAAGGTCAGCAGCCAGGACAAGGACAACAAC 976

Bx17 GACAAGGGCAGCAACCAGGACAAGGGCAACAAGGTCAGCAGCCAGGACAAGGACAACAAT 978

Bx20 GACAAGGGCAGCAACCAGGACAAGGGCAACAAGGTCAGCAGCCAGGACAAGGACAACAAC 991

Bx23 GACAAGGGCAGCAACCAGGACAAGGGCAACAAGGTCAGCAGCCAGGACAAGGACAACAAC 976

Bx23* GACAAGGGCAGCAACCAGGACAAGGGCAACAAGGTCAGCAGCCAGGACAAGGACAACAAC 976

******* ****** ********************************************

Bx6 CAGGACAAGGACAACAAGGATACTACCCAACTTCTCT-GCAACAGCTAGGACAAGGGCAA 1077

Bx7 CAGGACAAGGACAACAAGGATACTACCCAACTTCTCT-GCAACAGCCAGGACAAGGGCAA 1035

Bx7* CAGGACAAGGACAACAAGGATACTACCCAACTTCTCT-GCAACAGCCAGGACAAGGGCAA 1035

Bx13 CAGGACAAGGACAACAAGGATACTACCCAACTTCTCT-GCAACAGCCAGGACAAGGGCAA 1035

Bx14(+) CAGGACAAGGACAACAAGGATACTACCCAACTTCTCT-GCAACAGCCAGGACAAGGGCAA 1050

Bx14(-) CAGGACAAGGACAACAAGGATACTACCCAACTTCTCT-GCAACAGCCAGGACAAGGGCAA 1035

Bx14* CAGGACAAGGACAACAAGGATACTACCCAACTTCTCT-GCAACAGCCAGGACAAGGGCAA 1035

Bx17 CAGGACAAGGACAACAAGGATACTACCCAACTTCTCTTGCAACAGCCAGGACAAGGGCAA 1038

Bx20 CAGGACAAGGACAACAAGGATACTACCCAACTTCTCT-GCAACAGCCAGGACAAGGGCAA 1050

Bx23 CAGGACAAGGACAAGGAGGATACTACCCAACTTCTCT-GCAACAGCCAGGACAAGGGCAA 1035

Bx23* CAGGACAAGGACAAGGAGGATACTACCCAACTTCTCT-GCAACAGCCAGGACAAGGGCAA 1035

************** ********************* ******** *************

Bx6 CAACCGGGACAAGGGCAACCAGGGTACTACCCAACTTCGCAGCAGTCGGAACAAGGGCAA 1137

Bx7 CAACTGGGACAAGGGCAACCAGGGTACTACCCAACTTCGCAGCAGTCGGAACAAGGGCAG 1095

Bx7* CAACTGGGACAAGGGCAACCAGGGTACTACCCAACTTCGCAGCAGTCGGAACAAGGGCAG 1095

Bx13 CAACCGGGACAAGGGCAACCAGGGTACTACCCAACTTCGCAGCAGTCGGAACAAGGGCAG 1095

Bx14(+) CAACCGGGACAAGGGCAACCAGGGTACTACCCAACTTCTCCACAG--------------- 1095

Bx14(-) CAACCGGGACAAGGGCAACCAGGGTACTACCCAACTTCGCAGCAGTCGGAACAAGGGCAG 1095

Bx14* CAACCGGGACAAGGGCAATCAGGGTACTACCCAACTTCGCAGCAGTCGGAACAAGGGCAG 1095

Bx17 CAACTGGGACAAGGGCAACCAGGGTACTACCCAACTTCGCAGCAGTCGGAACAAGGGCAG 1098

Bx20 CAACCGGGACAAGGGCAACCAGGGTACTACCCAACTTCTCCACAG--------------- 1095

Bx23 CAACCGGGACAAGGGCAATCAGGGTACTACCCAACTTCGCAGCAGTCGGAACAAGGGCAG 1095

Bx23* CAACCGGGACAAGGGCAATCAGGGTACTACCCAACTTCGCAGCAGTCGGAACAAGGGCAG 1095

**** ************* ******************* * ***

Bx6 CAGCCAGGACAAGGAAAACAACCAGGACAAGGACAACAAGGGTACTACCCAACTTCTTCG 1197

Bx7 CAGCCAGGACAAGGAAAACAACCAGGACAAGGACAACAAGGGTACTACCCAACTTCTCCG 1155

Bx7* CAGCCAGGACAAGGAAAACAACCAGGACAAGGACAACAAGGGTACTACCCAACTTCTCCG 1155

Bx13 CAGCCAGGACAAGGAAAACAACCAGGACAAGGACAACAAGGGTACTACCCAACTTCTTCA 1155

Bx14(+) CAGCCAGGACAAGGAAAACAACCAGGACAAGGACAACAAAGGTACTACCCAACTTCTTCA 1155

Bx14(-) CAGCCAGGACAAGGAAAACAACCAGGACAAGGACAACAAGGGTACTACCCAACTTATTCA 1155

Bx14* CAGCCAGGACAAGGAAAACAACCAGGACAAGGACAACAAGGGTACTACCCAACTTCTTCA 1155

Bx17 CAGCCAGGACAAGGAAAACAACCAGGACAAGGACAACAAGGGTACTACCCAACTTCTCCG 1158

Bx20 CAGCCAGGACAAGGAAAACAACCAGGACAAGGACAACAAAGGTACTACCCAACTTCTTCA 1155

Bx23 CAGCCAGGACAAGGAAAACAACCAGGACAAGGACAACAAGGGTACTACCCAACTTCTTCA 1155

Bx23* CAGCCAGGACAAGGAAAACAACCAGGACAAGGACAACAAGGGTACTACCCAACTTCTTCA 1155

*************************************** *************** * *

Bx6 CAACAGTCAGGACAAGGGCAACAACCGGGACAAGGGCAACCAGGGTACTACCCAACTTCT 1257

Bx7 CAACAGTCAGGACAAGGGCAACAACTGGGACAAGGGCAACCAGGGTACTACCCAACTTCT 1215

Bx7* CAACAGTCAGGACAAGGGCAACAACTGGGACAAGGGCAACCAGGGTACTACCCAACTTCT 1215

Bx13 CAACAGTCAGGACAAGGGCAACAACTGGGACAAGGGCAACCAGGGTACTACCCAACTTCT 1215

Bx14(+) CAACAGTCAGGACAAGGGCAACAACCGGGACAAGGGCAACCAGGGTACTACCCAACTTCT 1215

Bx14(-) CAACAGTCAGGACAAGGGCAACAACTGGGACAAGGGCAACCAGGGTACTACCCAACTTCT 1215

Bx14* CAACAGTCAGGACAAGGGCAACAACTGGGACAAGGGCAACCAGGGTACTACCCAACTTCT 1215

Bx17 CAACAGTCAGGACAAGGGCAACAACTGGGACAAGGGCAACCAGGTTACTACCCAACTTCT 1218

Bx20 CAACAGTCAGGACAAGGGCAACAACCGGGACAAGGGCAACCAGGGTACTACCCAACTTCT 1215

Bx23 CAACAGTCAGGACAAGGGCAACAACTGGGACAAGGGCAACCAGGGTACTACCCAACTTCT 1215

Bx23* CAACAGTCAGGACAAGGGCAACAACTGGGACAAGGGCAACCAGGGTACTACCCAACTTCT 1215

************************* ****************** ***************

Bx6 CCACAGCAGTCAGGACAAGGACAACAATCAGGACAAGGACAACAAGGGTACTACCCAACT 1317

Bx7 CCACAGCAGTCAGGACAAGGACAACAATCAGGACAAGGACAACAAGGGTACTACCCAACT 1275

Bx7* CCACAGCAGTCAGGACAAGGACAACAATCAGGACAAGGACAACAAGGGTACTACCCAACT 1275

Bx13 CCACAGCAGTCAGGACAAGGACAACAATCAGGACAAGGACAACAAGGGTACTACCCAACT 1275

Bx14(+) CCACAGCAGTCAGGACAAGGACAACAATCAGGACAAGCACAACAAGGGTACTACCCAACT 1275

Bx14(-) CCACAGCAGTCAGGACAAGGACAACAATCAAGACAAGGACAACAAGGGTATTACCCAACT 1275

Bx14* CCACAGCAGTCAAGACAAGGACAACAATCAGGACAAGGACAACAAGGGTACTACCCAACT 1275

Bx17 CCACAGCAGTCAGGACAAGGACAACAATCAGGACAAGGACAACAAGGGTACTACCCAACT 1278

Bx20 CCACAGCAGTCAGGACAAGGACAACAATCAGGACAAGCACAACAAGGGTACTACCCAACT 1275

Bx23 CCACAGCAGTCAAGACAAGGACAACAATCAGGACAAGGACAACAAGGGTACTACCCAACT 1275

Bx23* CCACAGCAGTCAAGACAAGGACAACAATCAGGACAAGGACAACAAGGGTACTACCCAACT 1275

************ ***************** ****** ************ *********

PS8-F

Bx6 TCTCCGCAACAGTCAGGACAATGGCAACAACCGGGACAA**GGGCAATCGGGGTACTTCC**CA 1377

Bx7 TCTCCGCAACAGTCAGGACAAGGGCAACAACCGGGACAAGGGCAATCGGGGTACTTCCCA 1335

Bx7* TCTCCGCAACAGTCAGGACAAGGGCAACAACCGGGACAAGGGCAATCGGGGTACTTCCCA 1335

Bx13 TCTCCGCAACAGTCAGGACAAGGGCAACAACCGGGACAAGGGCAATCGGGGTACTTCCCA 1335

Bx14(+) TCTCCGCAACAGTCAGGACAAGGGCAACAACCGGGACAAAGGCAATCGGGGTACTTCCCA 1335

Bx14(-) TCTCCGCAACAGTCAGGACAAGGGCAACAACCGGGACAAGGGCAATCGGGGTACTTCCCA 1335

Bx14* TCTCCGCAACAGTCAGGACAAGGGCAACAACCGGGACAAGGGCAATCGGGGTACTTCCCA 1335

Bx17 TCTCCGCAACAGTCAGGACAAGGGCAACAACCGGGACAAGGGCAATCGGGGTACTTCCCA 1338

Bx20 TCTCCGCAACAGTCAGGACAAGGGCAACAACCGGGACAAAGGCAATCGGGGTACTTCCCA 1335

Bx23 TCTCCGCAACAGTCAGGACAAGGGCAACAACCGGGACAAGGGCAATCGGGGTACTTCCCA 1335

Bx23* TCTCCGCAACAGTCAGGACAAGGGCAACAACCGGGACAAGGGCAATCGGGGTACTTCCCA 1335

********************* ***************** ********************

Bx6 ACTTCTCGGCAGCAGTCAGGACAAGGGCAGCAGCTAGGACAAGGACAACAGTCGGGACAA 1395

Bx7 ACTTCTCGGCAGCAGTCAGGACAAGGGCAGCAGCCAGGACAAGGACAACAGTCGGGACAA 1395

Bx7* ACTTCTCGGCAGCAGTCAGGACAAGGGCAGCAGCCAGGACAAGGACAACAGTCGGGACAA 1395

Bx13 ACTTCTCGGCAGCAGTCAGGACAAGGGCAGCAGCCAGGACAAGGACAACAGTCGGGACAA 1395

Bx14(+) ACTTCTCGGCAGCAGTCAGGACAAGGGCAGCAGCCAGGACAAGGACAACAGTCAGGACAA 1395

Bx14(-) ACTTCTCGGCAGCAGTCAGGACAAGGGCAGCAGCCAGGACAAGGACAACAGTCGGGACAA 1395

Bx14* ACTTCTCGGCAGCAGTCAGGACAAGGGCAGCAGCCAGGACAAGGACAACAGTCGGGACAA 1395

Bx17 ACTTCTCGGCAGCAGTCAGGACAAGGGCAGCAGCCAGGACAAGGACAACAGTCGGGACAA 1398

Bx20 ACTTCTCGGCAGCAGTCAGGACAAGGGCAGCAGCCAGGACAAGGACAACAGTCAGGACAA 1395

Bx23 ACTTCTCGGCAGCAGTCAGGACAAGGGCAGCAGCCAGGACAAGGACAACAGTCGGGACAA 1395

Bx23* ACTTCTCGGCAGCAGTCAGGACAAGGGCAGCAGCCAGGACAAGGACAACAGTCGGGACAA 1395

********************************** ****************** ******

Bx6 GGGCAAGAAGGTCAGCAACCAGGACAAGGACAACAAGCGTACTACCCAGCTTCTTCGCAA 1455

Bx7 GGGCAACAAGGTCAGCAACCAGGACAAGGACAACAAGCGTACTACCCAACTTCTTCGCAA 1455

Bx7* GGGCAACAAGGTCAGCAACCAGGACAAGGACAACAAGCGTACTACCCAACTTCTTCGCAA 1455

Bx13 GGGCAACAAGGTCAGCAACCAGGACAGGGACAACAAGCGTACTACCCAACTTCTTCGCAA 1455

Bx14(+) GGGCAACAAGATCAGCAACCAGGACAAGGACAACAAGCGTACTACCCAACTTCTTCGCAA 1455

Bx14(-) GGGCAACAAGGTCAGCAACCAGGACAAGGACAACAAGCGTACTACCCAACTTCTTCGCAA 1455

Bx14* GGGCAACAAGGTCAGCAACCAGGACAAGGACAACAAGCGTACTACCCAACTTCTTCGCAA 1455

Bx17 GGGCAACAAGGTCAGCAACCAGGACAAGGACAACAAGCGTACTACCCAACTTCTTCGCAA 1458

Bx20 GGGCAACAAGATCAGCAACCAGGACAAGGACAACAAGCGTACTACCCAACTTCTTCGCAA 1455

Bx23 GGGCAACAAGGTCAGCAACCAGGACAAGGACAACAAGCGTACTACCCAACTTCTTCGCAA 1455

Bx23* GGGCAACAAGGTCAGCAACCAGGACAAGGACAACAAGCGTACTACCCAACTTCTTCGCAA 1455

****** *** *************** ********************* ***********

Bx6 CAGTCAGGACAAAGGCAACAGGCAGGACAATGGCAACGACCGGGACAAGGG-CAACCAAG 1556

Bx7 CAGTCAAGACAAAGGCAACAGGCAGGACAATGGCAACGACCGGGACAAGGG-CAACCAGG 1514

Bx7* CAGTCAAGACAAAGGCAACAGGCAGGACAATGGCAACGACCGGGACAAGGG-CAACCAGG 1514

Bx13 CAGTCAGGACAAAGGCAACAGGCAGGACAATGGCAACGACCGGGACAAGGG-CAATCAGG 1514

Bx14(+) CAGTCAGGACAAAGGCAACAGGCAGGACAATGGCAACGACCGGGACAAGGG-CAACCAGG 1514

Bx14(-) CAGTCAGGACAAAGGCAACAGGCAGGACAATGGCAACGACCGGGACAAGGG-CAACCAGG 1514

Bx14* CAGTCAGGACAAAGGCAACAGGCAGGACAATGGCAACGACCGGGACAAGGGC-AACCAGG 1514

Bx17 CAGTCAAGACAAAGGCAACAGGCAGGACAATGGCAACGACCGGGACAAGGG-CAACCAGG 1517

Bx20 CAGTCAGGACAAAGGCAACAGGCAGGACAATGGCAACGACCGGGACAAGGG-CAACCAGG 1514

Bx23 CAGTCAGGACAAAGGCAACAGGCAGGACAATGGCAACGACCGGGACAAGGG-CAACCAGG 1514

Bx23* CAGTCA-GACAAAGGCAACAGGCAGGACAATGGCAACGACCGGGACAAGGGCAAACCAGG 1514

****** ******************************************** ** ** *

PS14-F

Bx6 G**TACTACCCAACCTCTCCACA**GCAGCCAGGACAAGAGCAACAGTCAGGACAAACGCAACA 1616

Bx7 GTACTACCCAACCTCTCCACAGCAGCCAGGACAAGAGCAACAATCAGGACAAGCGCAACA 1574

Bx7* GTACTACCCAACCTCTCCACAGCAGCCAGGACAAGAGCAACAATCAGGACAAGCGCAACA 1574

Bx13 GTACTACCCAACCTCTCCACAGCAGCCAGGACAAGAGCAACAGTCAGGACAAGCGCAACA 1574

Bx14(+) GTACTACCCAACCTCTCCACAGCAGCCGGGACAAGAGCAACAGCCAGGACAAGCGCAACA 1574

Bx14(-) GTACTACCCAACCTCTCCACAGCAGCC------------------AGGACAAGCGCAACA 1556

Bx14* GTACTACCCAACCTCTCCACAGCAGCCAGGACAAGAGCAACAGTCAGGACAAGCGCAACA 1574

Bx17 GTACTACCCAACCTCTCCACAGCAGCCAGGACAAGAGCAACAATCAGGACAAGCGCAACA 1577

Bx20 GTACTACCCAACCTCTCCACAGCAGCCAGGACAAGAGCAACAGTCAGGACAAGCGCAACA 1574

Bx23 GTACTACCCAACCTCTCCACAGCAGCCAGGACAAGAGCAACAGTCAGGACAAGCGCAACA 1574

Bx23* GTACTACCCAACCTCTCCACAGCAGCCAGGACAAGAGCAACAGTCAGGACAAGCGCAACA 1574

*************************** ******* *******

PS13-F

Bx6 ATCAGGACAATGGCAACTAGTGTACTACCCAACTTCTCCGCAACAGCCAGGCCAATTGCA 1676

Bx7 ATCAGGACAATGGCAACTAGTGTACTACCCAACTTCTCCGCAACAGCCAGGCCAATTGCA 1634

Bx7* ATCAGGACAATGGCAACTAGTGTACTACCCAACTTCTCCGCAACAGCCAGGCCAATTGCA 1634

Bx13 ATCAGGACAATGGCAACTAGTGTACTACCCAACCTCTCCGCAACAGCCAGGCCAATTGCA 1634

Bx14(+) ATCAGGACAATGGCAACTAGTGTACTACCCAACTTCTCTGCAACAGCCAGGCCAATTGCA 1634

Bx14(-) ATCAGGACAATGGCAACTAGTGTACTACCCAACTTCTCCGCAACAGCCAGGCCAATTGCA 1616

Bx14* ATCAGGACAATGGCAACTAGTGTACTATCCAACTTCTCCGCAACAGCCAGGCCAATTGCA 1634

Bx17 ATCAGGACAATGGCAACTAGTGTACTACCCAACTTCTCCGCAACAGCCAGGCCAATTGCA 1637

Bx20 ATCAGGACAATGGCAACTA**GTGTACTACCCAACTTCTCT**GCAACAGCCAGGCCAATTGCA 1634

Bx23 ATCAGGACAATGGCAACTAGTGTACTACCCAACTTCTCCGCAACAGCCAGGCCAATTGCA 1634

Bx23* ATCAGGACAATGGCAACTAGTGTACTATCCAACTTCTCCGCAACAGCCAGGCCAATTGCA 1634

*************************** ***** **** *********************

PS14-R

Bx6 ACAACCAGCACAAGGGCAACAACCAGCACAAGGGCAACAATCAGCACAAGAGCAACAGCC 1736

Bx7 ACAACCAGCACAAGGGCAACAACCAGCACAAGGGCAACAATCAGCACAAGAGCAACAGCC 1694

Bx7* ACAACCAGCACAAGGGCAACAACCAGCACAAGGGCAACAATCAGCACAAGAGCAACAGCC 1694

Bx13 ACAACCAGCACAAGGGCAACAACCAGCACAAGGGCAACAATCAGCACAAGAGCAACAGCC 1694

Bx14(+) ACAACCAGCACAAGGGCAACAACCAGCACAAGGGCAACAATCAGCACAAGAGCAACAACC 1694

Bx14(-) ACAACCAGCACAAGGGCAACAACCAGCACAAGGGCAA**CAATCAGCACAAGAGCAACAG**CC 1676

Bx14* ACAACCAGCACAAGGGC------------------AACAATCAGCACAAGAGCAACAGCC 1676

Bx17 ACAACCAGCACAAGGGCAACAACCAGCACAAGGGCAACAATCAGCACAAGAGCAACAGCC 1697

Bx20 ACAACCAGCACAAGGGCAACAACCAGCACAAGGGCAACAATCAGCACAAGAGCAACAACC 1694

Bx23 ACAACCAGCACAAGGGCAACAACCAGCACAAGGGCAACAATCAGCACAAGAGCAACAGCC 1694

Bx23* ACAACCAGCACAAGGGC------------------AACAATCAGCACAAGAGCAACAGCC 1676

***************** ********************** **

Bx6 AGGACAAGCGCAACAATCAGGACAATGGCAACTAGTGTACTACCCAACTTCTCCGCAACA 1796

Bx7 AGGACAAGCGCAACAATCAGGACAATGGCAACTAGTGTACTACCCAACTTCTCCGCAACA 1754

Bx7* AGGACAAGCGCAACAATCAGGACAATGGCAACTAGTGTACTACCCAACTTCTCCGCAACA 1754

Bx13 AGGACAAGCGCAACAATCAGGACAATGGCAACTAGTGTACTACCCAACTTCTCCGCAACA 1754

Bx14(+) AGGACAAGCGCAACAATCAGGACAATGGCAACTAGTGTACTACCCAACTTCTCCGCAACA 1754

Bx14(-) AGGACAAGCGCAACAATCAGGACAATGGCAACTAGTGTACTACCCAACTTCTCCGCAACA 1736

Bx14* AGGACAAGCGCAACAATCAGGACAATGGCAACTAGTGTACTACCCAACTTCTCCACAACG 1736

Bx17 AGGACAAGCGCAACAATCAGGACAATGGCAACTAGTGTACTACCCAACTTCTC------- 1750

Bx20 AGGACAAGCGCAACAATCAGGACAATGGCAACTAGTGTACTACCCAACTTCTCCGCAACA 1754

Bx23 AGGACAAGCGCAACAATCAGGACAATGGCAACTAGTGTACTACCCAACTTCTCCGCAACA 1754

Bx23* AGGACAAGCGCAGCAATCAGGACAATGGCAACTAGTGTACTACCCAACTTCTCCACAACG 1736

************ ****************************************

Bx6 GCCAGGACAATTGCAACAACCAGCACAAGGGCAACAAGGGTACTACCCAACTTCTCCACA 1856

Bx7 GCCAGGACAATTGCAACAACCAGCACAAGGGCAACAAGGGTACTACCCAACTTCTCCACA 1814

Bx7* GCCAGGACAATTGCAACAACCAGCACAAGGGCAACAAGGGTACTACCCAACTTCTCCACA 1814

Bx13 GCCAGGACAATTGCAACAACCAACACAAGGGCAACAAGGGTACTACCCAACTTCTCCACA 1814

Bx14(+) GCCAGGACAATTGCAACAACCAGCACAAGGGCAACAAGGGTACTACCCAACTTCTCCACA 1814

Bx14(-) GCCAGGACAATTGCAACAACCAGCACAAGGGCAACAAGGGTACTACCCAACTTCTCCACA 1796

Bx14* GCCAGGACAATTGCAACAACCAGCACAAGGGCAACAAGGGTACTACCCAACTTCTCCACA 1796

Bx17 ------------------------------------------------------------ 1750

Bx20 GCCAGGACAATTGCAACAACCAGCACAAGGGCAACAAGGGTACTACCCAACTTCTCCACA 1814

Bx23 GCCAGGACAATTGCAACAACCAGCACAAGGGCAACAAGGGTACTACCCAACTTCTCCACA 1814

Bx23* GCCAGGACAATTGCAACAACCAGCACAAGGGCAACAAGGGTACTACCCAACTTCTCCACA 1796

Bx6 ACAGTCAGGACAAGGGCAACAAGGGTACTACACAACTTCTCTGCAACAGTCAGGACAAGG 1916

Bx7 ACAGTCAGGACAAGGGCAACAAGGGTACTACCCAACTTCTC------------------- 1855

Bx7* ACAGTCAGGACAAGGGCAACAAGGGTACTACCCAACTTCTC------------------- 1855

Bx13 ACAGTCAGGACAAGGGCAACAAGGGTACTACCCAACTTCTC------------------- 1855

Bx14(+) ACAGTCAGGACAAGGGCAACAAGGGTACTACCCAACTTCTC------------------- 1855

Bx14(-) ACAGTCGGGACAAGGGCAACAAGGGTACTACCCAACTTCTC------------------- 1837

Bx14* ACAGTCAGGACAAGGGCAACAAGGATACTACCCAACTTCTC------------------- 1837

Bx17 ------------------------------------------------------------ 1750

Bx20 ACAGTCAGGACAAGGGCAACAAGGGTACTACCCAACTTCTC------------------- 1855

Bx23 ACAGTCAGGACAAGGGCAACAAGGATACTACCCAACTTCTC------------------- 1855

Bx23* ACAGTCAGGACAAGGGCAACAAGGATACTACCCAACTTCTC------------------- 1837

Bx6 GCAACAAGGGTACTACCTAACTTCTCCGCAACAGTCAGGACAAGGGCAACAAGGGTACTA 1976

Bx7 --------------------------CGCAACAGTCAGGACAAGGGCAACAAGGGTACTA 1889

Bx7* --------------------------CGCAACAGTCAGGACAAGGGCAACAAGGGTACTA 1889

Bx13 --------------------------CGCAACAGTCAGGACAAGGGCAACAAGGGTACTA 1889

Bx14(+) --------------------------CGCAACAGTCAGGACAAGGGCAACAAGGGTACTA 1889

Bx14(-) --------------------------CGCAACAGTCAGGACAAGGGCAACAAGGGTACTA 1871

Bx14* --------------------------CGCAACAGTCAGGACAAGGGCAACAAGGGTACTA 1871

Bx17 --------------------------CGCAACAGTCAGGACAAGGGCAACAAGGGTACTA 1784

Bx20 --------------------------CGCAACAGTCAGGACAAGGGCAACAAGGGTACTA 1889

Bx23 --------------------------CGCAACAGTCAGGACAAGGGCAACAAGGGTACTA 1889

Bx23* --------------------------CGCAACAGTCAGGACAAGGGCAACAAGGGTACTA 1871

**********************************

PS8-R

Bx6 CCCAACTTCTCCACAACAGTCAGGACAAGGGCAGCAGCCAGGACAAGGACAACAGCCAAG 2036

Bx7 CCCAACTTCTCCGCAACAGTCAGGACAAGGGCAGCAGCCAGGACAAGGACAACAGCCAAG 1949

Bx7* CCCAACTTCTCCGCAACAGTCAGGACAAGGGCAGCAGCCAGGACAAGGACAACAGCCAAG 1949

Bx13 CCCAACTTCTCCGCAACAGTCAGGACAAGGGCAGCAGCCAGGACAAGGACGACAGCCAAG 1949

Bx14(+) CCCAACTTCTCCGCAACAGTCAGGACAAGGGCAGCAGCCAGGACAAGGACAACAGCCAAG 1949

Bx14(-) CCCAACTTCTCCGCAACAGTCAGGACAAGGGCAGCAGCCAGGACAAGGACAACAGCCAAG 1931

Bx14* CCCAACTTCTCCGCAACAGTCAGGACAAAGGCAGCAGCCAGGACAAGGACAACAGCCAAG 1931

Bx17 CCCAACTTCTCCGCAACAGTCAGGACAAGGGCAGCAGCCAGGACAAG**GACAACAGCCAAG** 1844

Bx20 CCCAACTTCTCCGCAACAGTCAGGACAAGGGCAGCAGCCAGGACAAGGACAACAGCCAAG 1949

Bx23 CCCAACTTCTCCGCAACAGTCAGGACAGGGGCAGCAGCCAGGACAAGGACAACAGCCAAG 1949

Bx23* CCCAACTTCTCCGCAACAGTCAGGACAAAGGCAGCAGCCAGGACAAGGACAACAGCCAAG 1931

************ ************** ********************* *********

Bx6 ACAAGGGCAACAAGGGTACTACCCAATTTCTCCGCAGCAGTCAGGACAAGGGCAACAACC 2096

Bx7 ACAAGGGCAACAAGGGTACTACCCAATTTCTCCGCAGCAGTCAGGACAAGGGCAACAACC 2009

Bx7* ACAAGGGCAACAAGGGTACTACCCAATTTCTCCGCAGCAGTCAGGACAAGGGCAACAACC 2009

Bx13 ACAAGGGCAACAAGGGTACTACCCAATTTCTCCGCAGCAGTCAGGACAAGGGCAACAACC 2009

Bx14(+) ACAAGGGCAACAAGGGTACTACCCAATTTCTCCGCAGCAGTCAGGACAAGGGCAACAAAC 2009

Bx14(-) ACAAGGGCAACAAGGGTACTACCCAATTTCTCCGCAGCAGTCAGGACAAGGGCTGCAACC 1991

Bx14* ACAAGGGCAACAAGGGTACTACCCAATTTCTCCGCAGCAGTCAGGACAAGGGCAACAACC 1991

Bx17 **ACAAGGG**CAACAAGGGTACTACCCAATTTCTCCGCAGCAGTCAGGACAAGGGCAACAACC 1904

Bx20 ACAAGGGCAACAAGGGTACTACCCAATTTCTCCGCAGCAGTCAGGACAAGGGCAACAAAC 2009

Bx23 ACAAGGGCAACAAGGGTACTACCCAATTTCTCCGCAGCAGTCAGGACAAGGGCAACAACC 2009

Bx23* ACAAGGGCAACAAGGGTACTACCCAATTTCTCCGCAGCAGTCAGGACAAGGGCAACAACC 1991

***************************************************** *** *

Bx6 AGGACAAGGGCAACAAGGATACTACCCAACTTCTCCGCAGCAGTCAGGACAAGGGCAACA 2156

Bx7 AGGACAAGGGCAACAAGGATACTACCCAACTTCTCCGCAGCAGTCAGGACAAGGGCAACA 2069

Bx7* AGGACAAGGGCAACAAGGATACTACCCAACTTCTCCGCAGCAGTCAGGACAAGGGCAACA 2069

Bx13 AGGACAAGGGCAACAAGGATACTACCCAACTTCTCCACAGCAGTCAGGACAAGGGCAACA 2069

Bx14(+) AGGACAAGGGCAACAAGGATACTACCCAACTTCTCCGCAGCAGTCAGGACAAGGGCAACA 2069

Bx14(-) AGGACAAGGGCAACAAGGATACTACCCAACTTCTCCGCAGCAGTCAGGACAAGGGCAACA 2051

Bx14* AGGACAATGGCAACAAGGATACTACCCAACTTCTCCGCAGCAGTCAGGACAAGGGCAACA 2051

Bx17 AGGACAAGGGCAACAAGGATACTACCCAACTTCTCCGCAGCAGTCAGGACAAGGGCAACA 1964

Bx20 AGGACAAGGGCAACAAGGATACTACCCAACTTCTCCGCAGCAGTCAGGACAAGGGCAACA 2069

Bx23 AGGACAATGGCAACAAGGATACTACCCAACTTCTCCGCAGCAGTCAGGACAAGGGCAACA 2069

Bx23* AGGACAATGGCAACAAGGATACTACCCAACTTCTCCGCAGCAGTCAGGACAAGGGCAACA 2051

******* **************************** ***********************

PS13-R

Bx6 ACCAGGACATGAGCAACAGCCAGGACAATGGTTGCAACCAGGACAAGGGCAACAAGGGTA 2216

Bx7 ACCAGGACATGAGCAACAGCCAGGACAATGGCTGCAACCAGGACAAGGGCAACAAGGGTA 2129

Bx7* ACCAGGACATGAGCAACAGCCAGGACAATGGCTGCAACCAGGACAAGGGCAACAAGGGTA 2129

Bx13 ACCAGGACATGAGCAACAGCCAGGACAATGGCTGCAACTAGGACAAGGGCAACAAGGGTA 2129

Bx14(+) ACCAAGGCATGAGCAACAGCCAGGACAATGGCTGCAACCAGGACAAGGGCAACAAGGGTA 2129

Bx14(-) ACCAGGACATGAGCAACAGCCAGGACAATGGCTGCAACCAGGACAAGGGCAACAAGGGTA 2111

Bx14* ACCAGGACATGAGCAACAGCCAGGACAATGGCTGCAACCAGGACAAGGGCAACAAGGGTA 2111

Bx17 ACCAGGACATGAGCAACAGCCAGGACAATGGCTGCAACCAGGACAAGGGCAACAAGGGTA 2024

Bx20 ACCA**AGACATGAGCAACAGCCAGG**ACAATGGCTGCAACCAGGACAAGGGCAACAAGGGTA 2129

Bx23 ACCAGGACATGAGCAACAGCCAGGACAATGGCTGCAACCAGGACAAGGGCAACAAGGGTA 2129

Bx23* ACCAGGACATGAGCAACAGCCAGGACAATGGCTGCAACCAGGACAAGGGCAACAAGGGTA 2111

**** * ************************ ****** *********************

PS7-F

Bx6 CTATCCAACTTCTTCACAGCAGTCAGGACAAGGGCAGCAATCAGGACAAGGGCAACAAGG 2276

Bx7 CTATCCAACTTCTTCACAGCAGTCAGGACAAGGGCATCAATCAGGACAAGGGCAACAAGG 2189

Bx7* CTAT**CCAACTTCTTCACAGCAGT**CAGGACAAGGGCATCAATCAGGACAAGGGCAACAAGG 2189

Bx13 CTATCCAACTTCTCCACAGCAGTCAGGACAAGGGCAGCAATCAGGACAAGGGCAACAAGG 2189

Bx14(+) CTATCCAACCTCTTCACAGCAGTCAGGACAAGGGCAGCAATCAGGACAAGGGCAACAAGG 2189

Bx14(-) CTATCCAACTTCTTCACAGCAGTCAGGACAAGGGCAGCAATCAGGACAAGGGCAACAAGG 2171

Bx14* CTATCCAACTTCTTCACAGCAGTCAGGACAAGGGCAGCAATCAGGACAAGGGCAACAAGG 2171

Bx17 CTATCCAACTTCTTCACAGCAGTCAGGACAAGGGCATCAATCAGGACAAGGGCAACAAGG 2084

Bx20 CTATCCAACCTCTTCACAGCAGTCAGGACAAGGGCAGCAATCAGGACAAGGGCAACAAGG 2189

Bx23 CTATCCAACTTCTTCACAGCAGTCAGGACAAGGGCAGCAATCAGGACAAGGGCAACAAGG 2189

Bx23* CTATCCAACTTCTTCACAGCAGTCAGGACAAGGGCAGCAATCAGGACAAGGGCAACAAGG 2171

********* *** ********************** ***********************

Bx6 GTACTACCCAACTTCTCTGTGGCAACCAGGACAAGGGCAACAACCAGGACAAGGGCAACA 2336

Bx7 GTACTACCCAACTTCTCTGTGGCAACCAGGACAAGGGCAACAACCAGGACAAGGGCAACA 2249

Bx7* GTACTACCCAACTTCTCTGTGGCAACCAGGACAAG------------------GGCAACA 2231

Bx13 GTACTACCCAACTTCTCTGTGGCAACCAGGACAAGGGCAACAACCAGGACAAGGGCAACA 2249

Bx14(+) GTACTACCCAACTTCTCTGTGGCAACCAGGACAAGGGCAACAACCAGGACAAAGGCAACA 2249

Bx14(-) GTACTACCCAACTTCTCTGTGGCAACCAGGACAAGGGCAACAACCAGGACAAGGGCAACA 2231

Bx14* GTACTACCCAACTTCTCTGTGGCAACCAGGACAAGGGCAACAACCAGGACAAGGGCAACA 2231

Bx17 GTACTACCCAACTTCTCTGTGGCAACCAGGACAAG------------------GGCAACA 2126

Bx20 GTACTACCCAACTTCTCTGTGGCAACCAGGACAAGGGCAACAACCAGGACAAAGGCAACA 2249

Bx23 GTACTACCCAACTTCTCTGTGGCAACCAGGACAAGGGCAACAACCAGGACAAGGGCAACA 2249

Bx23* GTACTACCCAACTTCTCTGTGGCAACCAGGACAAGGGCAACAACCAGGACAAGGGCAACA 2231

*********************************** *******

PS7-R

Bx6 AGGCTACGACAGTCCATACCATGTTAGCGCGGAGTACCAGGCGGCCCGCCTAAAGGTGGC 2396

Bx7 AGGCTACGCCAGCCCATACCATGTTAGCGCGGAGTACCAGGCGGCCCGCCTAAAGGTGGC 2309

Bx7* AGGCTACGCCAGCCCATACCATGTTAGCGCGGAGTACCAGGCGGCCCGC**CTAAAGGTGGC** 2291

Bx13 AGGCTACGACAGCCCATACCATGTTAGCGCGGAGTACCAGGCGGCCCGCCTAAAGGTGGC 2309

Bx14(+) AGGCTACGACAGCCCATACCATGTTAGCGCGGAGTACCAGGCGGCCCGCCTAAAGGTGGC 2309

Bx14(-) AGGTTACGACAGTCCATACCATGTTAGCGCGGAGTACCAGGCGGCCCGCCTAAAGGTGGC 2291

Bx14* AGGCTACGACAGCCCATACCATGTTAGCGCGGAGTACCAGGCGGCCCGCCTAAAGGTGGC 2291

Bx17 AGGCTACGCCAGCCCATACCATGTTAGCGCGGAGTACCAGGCGGCCCGCCTAAAGGTGGC 2186

Bx20 AGGCTACGACAGCCCATACCATGTTAGCGCGGAGTACCAGGCGGCCCGCCTAAAGGTGGC 2309

Bx23 AGGCTACGACAGCCCATACCATGTTAGCGCGGAGTACCAGGCGGCCCGCCTAAAGGTGGC 2309

Bx23* AGGCTACGACAGCCCATACCATGTTAGCGCGGAGTACCAGGCGGCCCGCCTAAAGGTGGC 2291

*** **** *** ***********************************************

Bx6 AAAGGCGCAACAGCTCGCGGCACAGCTGCCGGCAATGTGCCGGCTGGAGGGCAGCGACAC 2456

Bx7 AAAGGCGCAGCAGCTCGCGGCACAGCTGCCGGCAATGTGCCGGCTGGAGGGCAGCGACGC 2369

Bx7* **AAAGGCGCA**GCAGCTCGCGGCACAGCTGCCGGCAATGTGCCGGCTGGAGGGCAGCGACGC 2351

Bx13 AAAGGCGCAGCAGCTCGCGGCATCGCTGCCGGCAATGTGCCGGCTGGAGGGCAGCGACGC 2369

Bx14(+) AAAGGCGCAGCAGCTCGCGGCACAGCTGCCGGCAATGTGCCGGCTGGAGGGCAGCGACGC 2369

Bx14(-) AAAGGCGCAGCAGCTCGCGGCACAGCTGCCGGCAATGTGCCGGCTGGTGGGCAGCGACGC 2351

Bx14* AAAGGCGCAGCAGCTCGCGGCACAGCTGCCGGCAATGTGCCGGCTGGAGGGCAGCGACGC 2351

Bx17 AAAGGCGCAGCAGCTCGCGGCACAGCTGCCGGCAATGTGCCGGCTGGAGGGCAGCGACGC 2246

Bx20 AAAGGCGCAGCAGCTCGCGGCACAGCTGCCGGCAATGTGCCGGCTGGAGGGCAGCGACGC 2369

Bx23 AAAGGCGCAGCAGCTCGCGGCACAGCTGCCGGCAATGTGCCGGCTGGAGGGCAGCGACGC 2369

Bx23* AAAGGCGCAGCAGCTCGCGGCACAGCTGCCGGCAATGTGCCGGCTGGAGGGCAGCGACGC 2351

********* ************ *********************** ********** *

Bx6 ATTGTCGGCCAGGCAGTGA 2475

Bx7 ATTGTCGACCAGGCAGTGA 2388

Bx7* ATTGTCGACCAGGCAGTGA 2370

Bx13 ATTGTCGACCAGGCAGTGA 2388

Bx14(+) ATTGTCGGCCAGGCAGTGA 2388

Bx14(-) ATTGTCGGCCAGCCAGTGA 2370

Bx14* ATTGTCGGCCAGGCAGTGA 2370

Bx17 ATTGTCGACCAGGCAGTGA 2265

Bx20 ATTGTCGGCCAGGCAATGA 2388

Bx23 ATTGTCGACCAGGCAATGA 2388

Bx23* ATTGTCGGCCAGGCAGTGA 2370

******* **** ** ***

**Fig. S2.** The sequences and position of primers in the promoter region of *Glu*-*1Bx*.

PS2-F

**CCTCAGCATGCAAACATGCAGC**ATAATTTCCATTTTACTTGGCTATTTATGTTTGATAAATATTTCACAAATATACAATAATCAAAAACAATAAATTATA

TGTGTTTTTAGTTTTAGTTCTCATATCCAAATATACATGTTTCATACAACCAAATCTCATTTAAATATATTGTAAAATATTCCGGCAACAACTTGTGGGG

(43 bp insertion) PS5-F

GCCTTAAATATATTGTAAAATATTCCGGCAACAACTTGTGGGGTACATCTAGTTACAGTGGAATATTAGTGATGGCGTGACCAAGC**GATAAGGCCAACGA**

PS6-F

**GAGAAGAAG**TGCGTCGTCTATGGAGGCCAGGGAAAGACAATGGACATGCAAAGAGGT**AGGGGCAGGGAAGAAACACT**TGGAGATCATAGAAGAACATAAG

**↓**(185 bp insertion site)

AGGTTAAACATAGGA**GG**GCATAATGGACAATTAAATCTACATTAATTGAACTCATTTGGGAAGTAAACAAAATCCATATTCTGGTGTAAATCAAA**CTATT**

PS5-R PS2-R

**TGACGCGGATTTACTAAGATC**CTATGTTAATTTTA**GACATGACTGGCCAAAGGTTTCAG**TTAGTTCATTTGTCACGGAAAGGTGTTTTCATAAGTCCAAA

PS6-R

ACTCTACCAACTTTTTTGCACGTCATAGCATAGATAGATGTTGTGAGTCATTGGATAGATATTGTGAGTCAG**CATGGATTTGTGTTGCCTGG**AAATCCAA

PS11-F

CTAAATGACAAGCAACAAAACCTGAAAT**GGGCTTTAGGAGAGATGGTTTAT**CAATTTACATGTTCCATGCAGGCTACCTTCCACTACTCGACATGGTTAG

(54 bp insertion)

AAGTTTTGAGTGCCGCATATTTGCGGAAGCAATGGCACTACTCGACATGGTTAGAAGTTTTGAGTGCCGCATATTTGCGGAAGCAATGGCTAACAGATAC

ATATTCTGCCAAACCCCAAGAAGGATAATCACTCCTCTTAGATAAAAAGAACAGACCAATGTACAAACATCCACACTTCTGCAAACAATACACCAGAACT

AGGATTAAGCCCATTACGTGGCTTTAGCAGACCGTCCAAAAATCTGTTTTGCAAGCACCAATTGCTCCTTACTTATCCAGCTTCTTTTGTGTTGGCAAAC

PS11-R

TGCCCTTTTCCAACCGATTTTGTTCT**TCTCACGCTTTCTTCATAGGC**TAAACTAACCTCGGCGTGCACACAACCATGTCCTGAACCTTCACCTCGTCCCT

ATAAAAGCCCATCCAACCTTCACAATCTCATCATCACCCACAACACCGAGCACCCCAATCTACAGATCAATTCACTGACAGTTCACTGAG

**Figure S3.** The sequences primer positions of *Glu-1Bx14*^(-)^ and *Glu-1Bx14*^(+)^.

Bx14(+) ATGGCTAAGCGCCTGGTCCTCTTTGCGGCAGTAGTCGTCGCCCTCATGGCTCTCACCGCC 60

Bx14(-) ATGGCTAAGCGGTTGGTCCTCTTTGCGGCAGTAGTCGTCGCCCTCGTGGCTCTCACCGCC 60

*********** ******************************** **************

Bx14(+) GCTGAAGGTGAGGCCTCTGGACAACTACAATGTGAGCGCGAGCTCCGGAAGCGCGAGCTC 120

Bx14(-) GCTGAAGGTGAGGCCTCTGGACAACTACAATGTGAGCGCGA---------------GCTC 105

***************************************** ****

Bx14(+) GAGGCATACCAACAGGTGGTGGACCAGCAACTCCGAGACGTTAGCCCCGGGTACCGCCCC 180

Bx14(-) GAGGCATGCCAACAGGTGGTGGACCAGCAACTCCGAGACGTTAGCCCCGGGTGCCGCCCC 165

******* ******************************************** *******

Bx14(+) ATCACCGTCAGCCCGGGCACGAGACAATACGAGCAGCAACCTGTGGTGCCGCCCAAGGCC 240

Bx14(-) ATCACCGTCAGCCCGGGCACGAGACAATACGAGCAGCAACCTGTGGTGCCGTCCAAGGCC 225

*************************************************** ********

Bx14(+) GGATCCTTCTACCCCAGCGAGACTACGCCTTCGCAGCAACTCCAACAAATGATATTTTGG 300

Bx14(-) GGATCCTTCTACCCCAGCGAGACTACGCCTTCGCAGCAACTCCAACAAATGATATTTTGG 285

************************************************************

Bx14(+) GGAATACCTGCACTACTAAGAAGGTATTACCCAAGTGTAACTTCTTCGCAGCAGGGGTCA 360

Bx14(-) GGAATACCTGCACTACTAAGAAGGTATTACCCAAGTGTAACTTCTTCGCAGCAGGGGTCA 345

************************************************************

Bx14(+) TACTATCCAGGCCAAGCTTTTCCGCAACAATCAGGACAAGGACAGCAGCCAGGACAAGGA 420

Bx14(-) TACTATCCAGGCCAAGCTTCTCCGCAACAGTCAGGACAAGGACAGCAGCCAGGACAAGGA 405

******************* ********* ******************************

Bx14(+) CAGCAACCAGGACAAAGGCAACAAGATCAGCAGCCAGGACAAGGACAACAAGGGTACTAC 480

Bx14(-) CAGCAACCAGGACAAGGGCAACAAGATCAGCAACCAGGACAAAGACAACAAGGATACTAT 465

*************** **************** ********* ********** *****

Bx14(+) CCAACTTCTCCGCAACAGCCAGGACAAGGGCAACAACTGGGACAAGGGCAACCAGGGTAC 540

Bx14(-) CCAACTTCTCCGCAACAGCCAGGACAAGGGCAACAATTGGGACAAGGGCAACCAGGGTAC 525

************************************ ***********************

Bx14(+) TACCCAACTTCACAGCAGCCAGGACAAAAGCAGCAGGCAGGACAAGGGCAACAATCAGGA 600

Bx14(-) TACCCAACTTCACAGCAGCCAGGACAAAAGCAGCAGCCAGGACAAGGGCAACAATCAGGA 585

************************************ ***********************

Bx14(+) CAAGGACAACAAAGGTACTACCCAACTTCCCCGCAACAGTCAGGACAAGGGCAACAACCG 660

Bx14(-) CAAGGACAACAAGGGTACTACCCAACTTCCCCGCAACAGTCAGGACAAGGGCAACAACCG 645

************ ***********************************************

Bx14(+) GGACAAGGGCAACCAGGGTACTACCCAATTTCTCCGCAGCAGTCAGAACAATGGCAGCAA 720

Bx14(-) GGACAAGGGCAACCAGGGTACTACCCAAGTTCTCCGCAGCAGTCAGGACAATGGCAGCAA 705

**************************** ***************** *************

Bx14(+) CCAGGACAAGGGCAACAGCCAGGACAAGGGCAGCAATCGGGACAAGGGCAACAAGGTCAG 780

Bx14(-) CCAGGACAAGGGCAACAGCCAGGACAAGGGCAGCAATCAGGACAAGGGCAACAAGGTCAG 765

************************************** *********************

Bx14(+) CAGCCAGGACAAGGGCAACGACCAGGACAAGGACAACAAGGGTACTACCCAACTTCTCTG 840

Bx14(-) CAGCCAGGACAAGGGCAACGACCGGGACAAGGACAACAAGGGTACTACCCAACTTCTTCG 825

*********************** ********************************* *

Bx14(+) CAACAGCCGAGACAAGGGCAACAATCAGGACAAGGGCAACCAGGGTACTACCCAACTTCT 900

Bx14(-) CAACAGCCGGGACAAGGGCAACAATCAGGACAAGGGCAACCAGGGTACTACCCAACTTCT 885

********* **************************************************

Bx14(+) TCGCGGCAACCAGGACAATGGCAGCAACCAGGACAAGGGCAGCAACCAGGACAAGGGCAA 960

Bx14(-) TTGCGGCAGCCAGGACAATGGCAGCAACCAGGACAAGGGCAGCAATCAGGACAAGGGCAA 945

* ****** ************************************ **************

Bx14(+) CAAGGTCAGCAGCCAGGACAAGGACAACAACCAGGACAAGGACAACAAGGATACTACCCA 1020

Bx14(-) CAAGGTCAGCAGCCAGGACAAGGACAACAACCAGGACAAGGACAACAAGGATACTACCCA 1005

************************************************************

Bx14(+) ACTTCTCTGCAACAGCCAGGACAAGGGCAACAACCGGGACAAGGGCAACCAGGGTACTAC 1080

Bx14(-) ACTTCTCTGCAACAGCCAGGACAAGGGCAACAACCGGGACAAGGGCAACCAGGGTACTAC 1065

************************************************************

Bx14(+) CCAACTTCTCCACA---------------GCAGCCAGGACAAGGAAAACAACCAGGACAA 1125

Bx14(-) CCAACTTCGCAGCAGTCGGAACAAGGGCAGCAGCCAGGACAAGGAAAACAACCAGGACAA 1125

******** * ** *******************************

Bx14(+) GGACAACAAAGGTACTACCCAACTTCTTCACAACAGTCAGGACAAGGGCAACAACCGGGA 1185

Bx14(-) GGACAACAAGGGTACTACCCAACTTATTCACAACAGTCAGGACAAGGGCAACAACTGGGA 1185

********* *************** ***************************** ****

Bx14(+) CAAGGGCAACCAGGGTACTACCCAACTTCTCCACAGCAGTCAGGACAAGGACAACAATCA 1245

Bx14(-) CAAGGGCAACCAGGGTACTACCCAACTTCTCCACAGCAGTCAGGACAAGGACAACAATCA 1245

************************************************************

Bx14(+) GGACAAGCACAACAAGGGTACTACCCAACTTCTCCGCAACAGTCAGGACAAGGGCAACAA 1305

Bx14(-) AGACAAGGACAACAAGGGTATTACCCAACTTCTCCGCAACAGTCAGGACAAGGGCAACAA 1305

****** ************ ***************************************

Bx14(+) CCGGGACAAAGGCAATCGGGGTACTTCCCAACTTCTCGGCAGCAGTCAGGACAAGGGCAG 1365

Bx14(-) CCGGGACAAGGGCAATCGGGGTACTTCCCAACTTCTCGGCAGCAGTCAGGACAAGGGCAG 1365

********* **************************************************

Bx14(+) CAGCCAGGACAAGGACAACAGTCAGGACAAGGGCAACAAGATCAGCAACCAGGACAAGGA 1425

Bx14(-) CAGCCAGGACAAGGACAACAGTCGGGACAAGGGCAACAAGGTCAGCAACCAGGACAAGGA 1425

*********************** **************** *******************

Bx14(+) CAACAAGCGTACTACCCAACTTCTTCGCAACAGTCAGGACAAAGGCAACAGGCAGGACAA 1485

Bx14(-) CAACAAGCGTACTACCCAACTTCTTCGCAACAGTCAGGACAAAGGCAACAGGCAGGACAA 1485

************************************************************

PS14-F

Bx14(+) TGGCAACGACCGGGACAAGGGCAACCAGGG**T**ACTACCCAACCTCTCCACAGCAGCCGGGA 1545

Bx14(-) TGGCAACGACCGGGACAAGGGCAACCAGGG**TACTACCCAACCTCTCCACA**GCAGCCAGGA 1545

******************************************************** ***

Bx14(+) CAAGAGCAACAGCCAGGACAAGCGCAACAATCAGGACAATGGCAACTAGTGTACTACCCA 1605

Bx14(-) ------------------CAAGCGCAACAATCAGGACAATGGCAACTAGTGTACTACCCA 1587

******************************************

Bx14(+) ACTTCTCTGCAACAGCCAGGCCAATTGCAACAACCAGCACAAGGGCAACAACCAGCACAA 1665

Bx14(-) ACTTCTCCGCAACAGCCAGGCCAATTGCAACAACCAGCACAAGGGCAACAACCAGCACAA 1647

******* ****************************************************

PS14-R

Bx14(+) GGGCAACAATCAGCACAAGAGCAACAACCAGGACAAGCGCAACAATCAGGACAATGGCAA 1725

Bx14(-) GGGCAA**CAATCAGCACAAGAGCAACAG**CCAGGACAAGCGCAACAATCAGGACAATGGCAA 1707

************************** *********************************

Bx14(+) CTAGTGTACTACCCAACTTCTCCGCAACAGCCAGGACAATTGCAACAACCAGCACAAGGG 1785

Bx14(-) CTAGTGTACTACCCAACTTCTCCGCAACAGCCAGGACAATTGCAACAACCAGCACAAGGG 1767

************************************************************

Bx14(+) CAACAAGGGTACTACCCAACTTCTCCACAACAGTCAGGACAAGGGCAACAAGGGTACTAC 1845

Bx14(-) CAACAAGGGTACTACCCAACTTCTCCACAACAGTCGGGACAAGGGCAACAAGGGTACTAC 1827

*********************************** ************************

Bx14(+) CCAACTTCTCCGCAACAGTCAGGACAAGGGCAACAAGGGTACTACCCAACTTCTCCGCAA 1905

Bx14(-) CCAACTTCTCCGCAACAGTCAGGACAAGGGCAACAAGGGTACTACCCAACTTCTCCGCAA 1887

************************************************************

Bx14(+) CAGTCAGGACAAGGGCAGCAGCCAGGACAAGGACAACAGCCAAGACAAGGGCAACAAGGG 1965

Bx14(-) CAGTCAGGACAAGGGCAGCAGCCAGGACAAGGACAACAGCCAAGACAAGGGCAACAAGGG 1947

************************************************************

Bx14(+) TACTACCCAATTTCTCCGCAGCAGTCAGGACAAGGGCAACAAACAGGACAAGGGCAACAA 2025

Bx14(-) TACTACCCAATTTCTCCGCAGCAGTCAGGACAAGGGCTGCAACCAGGACAAGGGCAACAA 2007

************************************* *** *****************

Bx14(+) GGATACTACCCAACTTCTCCGCAGCAGTCAGGACAAGGGCAACAACCAAGGCATGAGCAA 2085

Bx14(-) GGATACTACCCAACTTCTCCGCAGCAGTCAGGACAAGGGCAACAACCAGGACATGAGCAA 2067

************************************************ * *********

Bx14(+) CAGCCAGGACAATGGCTGCAACCAGGACAAGGGCAACAAGGGTACTATCCAACCTCTTCA 2145

Bx14(-) CAGCCAGGACAATGGCTGCAACCAGGACAAGGGCAACAAGGGTACTATCCAACTTCTTCA 2127

***************************************************** ******

Bx14(+) CAGCAGTCAGGACAAGGGCAGCAATCAGGACAAGGGCAACAAGGGTACTACCCAACTTCT 2205

Bx14(-) CAGCAGTCAGGACAAGGGCAGCAATCAGGACAAGGGCAACAAGGGTACTACCCAACTTCT 2187

************************************************************

Bx14(+) CTGTGGCAACCAGGACAAGGGCAACAACCAGGACAAAGGCAACAAGGCTACGACAGCCCA 2265

Bx14(-) CTGTGGCAACCAGGACAAGGGCAACAACCAGGACAAGGGCAACAAGGTTACGACAGTCCA 2247

************************************ ********** ******** ***

Bx14(+) TACCATGTTAGCGCGGAGTACCAGGCGGCCCGCCTAAAGGTGGCAAAGGCGCAGCAGCTC 2325

Bx14(-) TACCATGTTAGCGCGGAGTACCAGGCGGCCCGCCTAAAGGTGGCAAAGGCGCAGCAGCTC 2307

************************************************************

Bx14(+) GCGGCACAGCTGCCGGCAATGTGCCGGCTGGAGGGCAGCGACGCATTGTCGGCCAGGCAG 2385

Bx14(-) GCGGCACAGCTGCCGGCAATGTGCCGGCTGGTGGGCAGCGACGCATTGTCGGCCAGCCAG 2367

******************************* ************************ ***

Bx14(+) TGA 2388

Bx14(-) TGA 2370

***

**Figure S4.** Alignment of the sequences of *Glu-1By* alleles and primer positions.

By8 ATGGCTAAGCGGTTGGTCCTCTTTGCGACAGTAGTCATCACCCTCGTGGCTCTCACTGCT 60

By9 ATGGCTAAGCGGTTGGTCCTCTTTGCGACAGTAGTCATCACCCTCGTGGCTCTCACTGCT 60

By15 ATGGCTAAGCGGTTGGTCCTCTTTGCGACAGTAGTCATCACCCTCGTGGCTCTCACTGCT 60

By15* ATGGCTAAGCGGTTGGTCCTTTTTGCGACAGTAGTCATCACCCTCGTGGCTCTCACTGCT 60

By16 ATGGCTAAGCGGCTGGTCCTCTTTGCGACAGTAGTCATCACCCTCGTGGCTCTCACTGCT 60

By18 ATGGCTAAGCGGTTGGTCCTCTTTGCGACAGTAGTCATCACCCTCGTGGCTCTCACTGCT 60

By20 ATGGCTAAGCGGCTGGTCCTCTTTGCGACAGTAGTCATCACCCTCGTGGCTCTCACTGCT 60

************ ******* ***************************************

By8 GCTGAAGGTGAGGCCTCTAGGCAACTACAGTGTGAGCGCGAGCTCCAGGAGAGCTCGCTT 120

By9 GCTGAAGGTGAGGCCTCTAGGCAACTACAGTGTGAGCGCGAGCTCCAGGAGAGCTCGCTT 120

By15 GCTGAAGGTGAGGCCTCTAGGCAACTACAGTGTGAGCGCGAGCTCCAGGAGAGCTCGCTT 120

By15* GCTGAAGGTGAGGCCTCTAGGCAACTACAGTGTGAGCGCGAGCTCCAGGAAAGCTCGCTT 120

By16 GCTGAAGGTGAGGCCTCTAGGCAACTACAGTGTGAGCGCGAGCTCCAGGAGAGCTCGCTT 120

By18 GCTGAAGGTGAGGCCTCTAGGCAACTACAGTGTGAGCGCGAGCTCCAGGAGAGCTCGCTT 120

By20 GCTGAAGGTGAGGCCTCTAGGCAACTACAGTGTGAGCGCGAGCTCCAGGAGAGCTCGCTT 120

************************************************** *********

By8 GAGGCATGCCGACAGGTCGTGGACCAACAGTTGGCCGGTCGGCTGCCATGGAGCACGGGG 180

By9 GAGGCATGCCGACAGGTCGTGGACCAACAGTTGGCCGGTCGGCTGCCATGGAGCACGGGG 180

By15 GAGGCATGCCGACAGGTCGTGGACCAACAGTTGGCCGGTCGGCTGCCATGGAGCACGGGG 180

By15* GAGGCATGCCGACAGGTCGTGGACCAACAGTTGGCCGGTCGGCTGCCATGGAGCACGGGG 180

By16 GAGGCATGCCGACAGGTCGTGGACCAACAGTTGGCCGGTCGGCTGCCATGGAGCACGGGG 180

By18 GAGGCATGCCGACAGGTCGTGGACCAACAGTTGGCCGGTCGGCTGCCATGGAGCACGGGG 180

By20 GAGGCATGCCGACAGGTCGTGGACCAACAGTTGGCCGGTCGGCTGCCATGGAGCACGGGG 180

************************************************************

PS15-F/PS16-F

By8 CTCCAGATGCGATGCTGCCAGCAGCTCCGAGATG**TTAGCGCTAAGTGCCGTCT**CGTCGCC 240

By9 CTCCAGATGCGATGCTGCCAGCAGCTCCGAGATGTTAGCGCTAAGTGCCGTCCCGTCGCC 240

By15 CTCCAGATGCGATGCTGCCAGCAGCTCCGAGATGTTAGCGCTAAGTGCCGCCCCGTCGCC 240

By15* CTCCAGATGCGATGCTGCCAGCAGCTCCGAGATGTTAGCGCTAAGTGCCGCCCCGTCGCC 240

By16 CTCCAGATGCGATGCTGCCAGCAGCTCCGAGATGTTAGCGCTAAGTGCCGTCCCGTCGCC 240

By18 CTCCAGATGCGATGCTGCCAGCAGCTCCGAGATG**TTAGCGCTAAGTGCCGTCC**CGTCGCC 240

By20 CTCCAGATGCGATGCTGCCAGCAGCTCCGAGATGTTAGCGCTAAGTGCCGCCCCGTCGCC 240

************************************************** * *******

By8 GTCAGCCAAGTCGTAAGACAATATGAGCAAACCGTGGTGCCGCCCAAGGGCGGATCCTTC 300

By9 GTCAGCCAAGTCGTAAGACAATATGAGCAAACCGTGGTGCCGCCCAAGGGCGGATCCTTC 300

By15 GTCAGCCAAGTCGTAAGACAATATGAGCAAACCGTGGTGCCGCCCAAGGGCGGATCCTTC 300

By15* GTCAGCCAAGTCGTAAGACAATATGAGCAAACCGTGGTGCCGCCCAAGGGCGGATCCTTC 300

By16 GTCAGCCAAGTCGTAAGACAATATGAGCAAATCGTGGTGCCGCCCAAGGGCGGATCCTTC 300

By18 GTCAGCCAAGTCGTAAGACAATATGAGCAAACCGTGGTGCCGCCCAAGGGCGGATCCTTC 300

By20 GTCAGCCAAGTCGTAAGACAATATGAGCAAACCGTGGTGCCGCCCAAGGGCGGATCCTTC 300

******************************* ****************************

By8 TACCCTGGCGAGACCACACCACTGCAGCAACTCCAACAAGTAATATTTTGGGGAACATCT 360

By9 TACCCTGGCGAGACCACACCACTGCAGCAACTCCAACAAGTAATATTTTGGGGAACATCT 360

By15 TACCCTGGCGAGACCACACCACTGCAGCAACTCCAACAAGTAATATTTTGGGGAACATCT 360

By15* TACCAAGGCGAGACCACACCACTACAACAACTCCAACAAGTAATATTTTGGGGAACATCT 360

By16 TACCCTGGCGAGACCACACCACTGCAGCAGCTCCAACAAGTAATATTTTGGGGAACATCT 360

By18 TACCCTGGCGAGACCACACCACTGCAGCAACTCCAACAAGTAATATTTTGGGGAACATCT 360

By20 TACCCTGGCGAGACCACACCACTGCAGCAACTCCAACAAGTAATATTTTGGGGAACATCT 360

**** ***************** ** ** ******************************

PS18-F

By8 TCACAAACAGTACAAGGGTATTACCCAAGCGTAAGTTCTCCTCAGCAGGGGCCATATTAT 420

By9 TCACAAACAGTACAAGGGTATTACCCAAGCGTAAGTTCTCCTCAGCA**GGGGCCATATTAT** 420

By15 TCACAAACAGTACAAGGGTATTACCCAAGCGTAAGTTCTCCTCAGCAGGGGCCATATTAT 420

By15* TCACAAACAGGACAAGGGTATTACCCAAGCATAAGTTCTCCTCAGCAGGGGCCATATTAT 420

By16 TCACAAACAGTACAAGGGTATTACCCAAGCGTAAGTTCTCCTCAGCAGGGGCCATATTAT 420

By18 TCACAAACAGTACAAGGGTATTACCCAAGCGTAAGTTCTCCTCAGCAGGGGCCATATTAT 420

By20 TCACAAACAGTACAAGGGTATTACCCAAGCGTAAGTTCTCCTCAGCAGGGGCCATATTAT 420

********** ******************* *****************************

By8 CCAGGCCAAGCTTCTCCACAACAGCCAGGACAAGGGCAACAGCCAGGCAAATGGCAAGAA 480

By9 **CCAGGCC**AAGCTTCTCCACAACAGCCAGGACAAGGGCAACAGCCAGGCAAATGGCAAGAA 480

By15 CCAGGCCAAGCTTCTCCACAACAGCCAGGACAAAGGCAACAGCCAGGCAAATGGCAAGAA 480

By15* CCAGGCCAAGCTTCTCCACAACAGCCAGGACAAGGGCAACAGCCAGGCAAATGGCAAGAA 480

By16 CCAGGCCAAGCTTCTCCACAACAGCCAGGACAAGGGCAACAGCCAGGCAAATGGCAAGAA 480

By18 CCAGGCCAAGCTTCTCCACAACAGCCAGGACAAGGGCAACAGCCAGGCAAATGGCAAGAA 480

By20 CCAGGCCAAGCTTCTCCACAACAGCCAGGACAAAGGCAACAGCCAGGCAAATGGCAAGAA 480

********************************* **************************

By8 CTGGGACAAGGGCAACAAGGGTACTACCCAACTTCTCTGCATCAGTCAGGACAAGGACAA 540

By9 CTGGGACAAGGGCAACAAGGGTACTACCCAACTTCTCTGCATCAGTCAGGACAAGGACAA 540

By15 CTGGGACAAGGGCAACAAGGGTATTACCCAACTTCTCTGCATCAGTCAGGACAAGGACAA 540

By15* CTGGGACAAGGGCAACAAGAGTACTACCCAACTTCTCTGCATCAGTCAGGACAAGGACAA 540

By16 CTGGGACAAGGGCAACAAGGGTACTACCCAACTTCTCTGCATCAGTCAGGACAAGGACAA 540

By18 CTGGGACAAGGGCAACAAGGGTACTACCCAACTTCTCTGCATCAGTCAGGACAAGGACAA 540

By20 CTGGGACAAGGGCAACAAGGGTATTACCCAACTTCTCTGCATCAGTCAGGACAAGGACAA 540

******************* *** ************************************

By8 CAAGGGTACTACCCATCTTCTCTGCAGCAACCAGGACAAGGGCAACAGATAGGACAAGGG 600

By9 CAAGGGTACTACCCATCTTCTCTGCAGCAACCAGGACAAGGGCAACAGATAGGACAAGGG 600

By15 CAAGGGTACTACCCATCTTCTCTGCAGCAACCAGGACAAGGGCAACAGACAGGACAAGGG 600

By15* CAAGGGTACTACCCATCTTCTCTGCAGCAACCAGGACAAGGGCAACAGACAGGACAAGTG 600

By16 CAAGGGTACTACCCATCTTCTCTGCAGCAACCAGGACAAGGGCAACAGACAGGACAAGGG 600

By18 CAAGGGTACTACCCATCTTCTCTGCAGCAACCAGGACAAGGGCAACAGATAGGACAAGGG 600

By20 CAAGGGTACTACCCATCTTCTCTGCAGCAACCAGGACAAGGGCAACAGACAGGACAAGGG 600

************************************************* ******** *

By8 CAACAAGGATACTACCCAACTTCTCTGCAGCAGCCAGGACAAGGGCAACAGATAGGACAA 660

By9 CAACAAGGATACTACCCAACTTCTCTGCAGCAGCCAGGACAAGGGCAACAGATAGGACAA 660

By15 CAACAAGGATACTACCCAACTTCTCTGCAGCAGCCAGGACAAGGGCAACAGATAGGACAA 660

By15* CAACAAGAATACTACCCAACTTCTCTACAGCAATCAGGACAAGGGCAACAAATAGGACAA 660

By16 CAACAAGGATACTACCCAACTTCTCTGCAGCAGCCAGGACAAGGGCAACAGATAGGACAA 660

By18 CAACAAGGATACTACCCAACTTCTCTGCAGCAGCCAGGACAAGGGCAACAGATAGGACAA 660

By20 CAACAAGGATACTACCCAACTTCTCTGCAGCAGCCAGGACAAGGGCAACAGATAGGACAA 660

******* ****************** ***** **************** *********

By8 GGACAACAAGGGTACTACCCAACTTCTCCGCAACACCCAGGACAAAGGCAACAACCAGGA 720

By9 GGACAACAAGGGTACTACCCAACTTCTCCGCAACACCCAGGACAAAGGCAACAACCAGGA 720

By15 GGGCAACAAGGGTACTACCCAACTTCTCCGCAGCACCCAGGACAAAGGCAACAACCAGGA 720

By15* GGGCAACAAGGGTACTACCCAACTTCTCCGCAGCACCCAGGACAAAGGCAACAACCAGGA 720

By16 GGACAACAAGGGTACTACCCAACTTCTCCGCAGCACCCAGGACAAAGGCAACAACCAGGA 720

By18 GGACAACAAGGGTACTACCCAACTTCTCCGCAACACCCAGGACAAAGGCAACAACCAGGA 720

By20 GGGCAACAAGGGTACTACCCAACTTCTCCGCAGCACCCAGGACAAAGGCAACAACCAGGA 720

** ***************************** ***************************

PS15-R/PS16-R/PS20-F/PS23-F

By8 C**AAGGGCAGCAAATAGGACAAG**GGCAACAACTAGGACAAGGGCGGCAAATAGGACAAGGG 780

By9 CAAGGGCAGCAAATAGGACAAGGGCAACAACTAGGACAAGGGCGGCAAATAGGACAAGGG 780

By15 CAA**GGGCAGCAAATAGGACAAG**GGCAACAACCAGGACAAGGGCGGCAAATAGGACAAGGG 780

By15* CAAGGGCAGCAAATAGGACAAGGGCAACAACCAGGACAAGGGCGGCAAATAGGACAAGGG 780

By16 CAA**GGGCAGCAAATAGGACAAGA**GCAACAACTAGGACAAGGGCGGCAAATAGGACAAGGG 780

By18 C**AAGGGCAGCAAATAGGACAAG**GGCAACAACTAGGACAAGGGCGGCAAATAGGACAAGGG 780

By20 CAAGGGCAGCAAATAGGACAAGGGCAACAACCAGGACAAGGGCGGCAAATAGGACAAGGG 780

********************** ******** ****************************

PS21-F

By8 CAACAATCAGGACAAGGGCAACAAGGGTACTATCCAACTTCTCCACAGCAGCTAGGACAA 840

By9 CAACAATCAGGACAAGGGCAACAAGGGTACTATCCAACTTCTCCACAGCAGCTAGGACAA 840

By15 CAACAATCAGGACAAGAGCAACAAGGGTACTATGCAACTTCTCCACAGCAGCTAGGACAA 840

By15* CAACAATCAGGACAAGGGCAACAAGGGTACTATCCAACTTCTCCACAGCAGCTAGGACAA 840

By16 CAACAATCAGGACAAGGGCAACAAGGGTACTATCCAACTTCTCCACAGCAGCTAGGACAA 840

By18 CAACAATCAGGACAAGGGCAACAAGGGTACTATCCAACTTCTCCACAGCAGCTAGGACAA 840

By20 CAACAATCAGGACAAGAGCAACAAGGGTACTATGCAACT**TCTCCACAGCAGCTAGGACG**A 840

**************** **************** ************************ *

By8 GGGCAACAACCAGGACAATGGCAACAATCAGGACAAGGGCAACAAGGGTACTACCCAACT 900

By9 GGGCAACAACCAGGACAATGGCAACAATCAGGACAAGGGCAACAAGGGTACTACCCAACT 900

By15 GGGCAACAACCAGGACAATGGCAACAATCAGGACAAGGGCAACAAAGGTACTACCCAACT 900

By15* GGGCAACAACCAGGACAATGGCAACAATCAGGACAAGGGCAACAAGGGTACTACCCAACT 900

By16 GGGCAACAACCAGGACAATGGCAACAATCAGGACAAGGGCAACAAGGGTACTACCCAACT 900

By18 GGGCAACAACCAGGACAATGGCAACAATCAGGACAAGGGCAACAAGGGTACTACCCAACT 900

By20 GGGCAACAACCAGGACAATGGCAACAATCAGGACAAGGGCAACAAAGGTACTACCCAACT 900

********************************************* **************

By8 TCTCAGCAGCAGCCAGGACAAGGGCAACAAGGGCAGTACCCAGCTTCTCAGCAGCAGCCA 960

By9 TCTCAGCAGCAGCCAGGACAAGGGCAACAAGGGCAGTACCCAGCTTCTCAGCAGCAGCCA 960

By15 TCTCAGCAGCAGCCAGGACAAGGGCAACAAGGGCAGTACCCAGCTTCTCAGCAGCAGCCA 960

By15* TCTCAGCAGCAGTCAGGACAAGGGCAACAAGGGCAGTACCCAGCTTCTCAGCAGCAGCCA 960

By16 TCCCAGCAGCAGCCAGGACAAGGGCAACAAGGGCAGTACCCAGCTTCTCAGCAGCAGCCA 960

By18 TCTCAGCAGCAGCCAGGACAAGGGCAACAAGGGCAGTACCCAGCTTCTCAGCAGCAGCCA 960

By20 TCTCAGCAGCAGCCAGGACAAGGGCAACAAGGGCAGTACCCAGCTTCTCAGCAGCAGCCA 960

** ********* ***********************************************

By8 GGACAAGGGCAACAAGGGCAGTACCCAGCTTCTCAGCAGCAGCCAGGACAAGGGCAACAA 1020

By9 GGACAAGGGCAACAAGGGCAGTACCCAGCTTCTCAGCAGCAGCCAGGACAAGGGCAACAA 1020

By15 GCACAAGGGCAACAAGGGCAGTACCCAGCTTCTCAGCAGCAGCCAGCACAAGGGCAACAA 1020

By15* GGACAAGGGCAACAAGGGCAGTACCCAGCTTCTCAGCAGCAGGCAGCACAAGGGCAACAA 1020

By16 GGACAAGGGCAACAAGGGCAGTACCCAGCTTCTCAGCAGCAGCCAGGACAAGGGCAACAA 1020

By18 GGACAAGGGCAACAAGGGCAGTACCCAGCTTCTCAGCAGCAGCCAGGACAAGGGCAACAA 1020

By20 GCACAAGGGCAACAAGGGCAGTACCCAGCTTCTCAGCAGCAGCCAGCACAAGGGCAACAA 1020

* **************************************** *** *************

By8 GGGCAGTACCCAGCTTCTCAGCAGCAGCCAGCACAAGGGCAACAAGGGCAGTACCCAGCT 1080

By9 GGGCAGTACCCAGCTTCTCAA--------------------------------------- 1041

By15 GGGCAGTACCCAGCTTCTCAGCAGCAGCCAGGACAAGGGCAACAAGGGCAGTACCCAGCT 1080

By15* GGGCAGTACCCAGCTTCTCAG--------------------------------------- 1041

By16 GGGCAGTACCCAGCTTCTCAGCAGCAGCCAGCACAAGGGCAACAAGGGCAGTACCCAGCT 1080

By18 GGGCAGTACCCAGCTTCTCAGCAGCAGCCAGCACAAGGGCAACAAGGGCAGTACCCAGCT 1080

By20 GGGCAGTACCCAGCTTCTCAGCAGCAGCCAGGACAAGGGCAACAAGGGCAGTACCCAGCT 1080

********************

PS21-R

By8 TCTCAACAACAGCCAGGACAAGGGCAACAAGGGCACTACCTAGCTTCTCAGCAGCAGCCA 1140

By9 ------CAACAGCCAGGACAAGGGCAACAAGGGCACTACCTAGCTTCTCAGCAGCAGCCA 1095

By15 TCTCAGCAGCAGCCAGCACAAGGGCAACAAGGGCAGTACCCAGCTTCTCAACAGCAGCCA 1140

By15* ------CAGCAGCCAGGACAAGGGCAACAAGGGCAGTACCCAGCTTCTCAACAGCAGCCA 1095

By16 TCTCAACAACAGCCAGGACAAGGGCAACAAGGGCACTACCTAGCTTCTCAGCAGCAGCCA 1140

By18 TCTCAACAACAGCCAGGACAAGGGCAACAAGGGCACTACCTAGCTTCTCAGCAGCAGCCA 1140

By20 TCTCAGCAGCAGCCAGCACAAGGGCAACAAGGGCA**GTACCCAGCTTCTCAACAGC**AGCCA 1140

** ******* ****************** **** ********* *********

By8 GGACAAGGGCAACAACGGCACTACCCAGCTTCTCTGCAGCAACCAGGACAAGGGCAACAA 1200

By9 GGACAAGGGCAACAACGGCACTACCCAGCTTCTCTGCAGCAACCAGGACAAGGGCAACAA 1155

By15 GGACAAGGGCAACAAGGGCACTACCCAGCTTCTGAGCAGCAGCCAGGACAAGGGCAACAA 1200

By15* GGACAAGGGCAACAAGGGCACTACCCAGCTTCTCAGCAGCAGCCAGGACAAGGGCAACAA 1155

By16 GGACAAGGGCAACAACGGCACTACCCAGCTTCTCTGCAGCAACCAGGACAAGGGCAACAA 1200

By18 GGACAAGGGCAACAACGGCACTACCCAGCTTCTCTGCAGCAACCAGGACAAGGGCAACAA 1200

By20 GGACAAGGGCAACAAGGGCACTACCCAGCTTCTGAGCAGCAGCCAGGACAAGGGCAACAA 1200

*************** ***************** ****** ******************

PS19-R

By8 GGGCATTACACAGCTTCTCTGCAGCAACCAGGACAAGGGCAACAAGGGCATTACCCAGCT 1260

By9 G**GGCATTACACAGCTTCTCT**GCAGCAACCAGGACAAGGGCAACAAGGGCATTACCCAGCT 1215

By15 CGGCACTACCCAGCTTCTCTGCAGCAACCAGGACAAGGGCAACAAAGGCATTACGCAGCT 1260

By15* CGGCATTACCCAGCTTCTCTGCAGCAACCAGGACAAGGGCAACAAGGGCATTACACAGCT 1215

By16 GGGCATTACACAGCTTCTCTGCAGCAACCAGGACAAGGGCAACAAGGGCATTACCCAGCT 1260

By18 GGGCATTACACAGCTTCTCTGCAGCAACCAGGACAAGGGCAACAAGGGCATTACCCAGCT 1260

By20 CGGCACTACCCAGCTTCTCTGCAGCAACCAGGACAAGGGCAACAAAGGCATTACGCAGCT 1260

**** *** *********************************** ******** *****

PS23-R PS18-R/PS20-R

By8 TCTCTGCAGCAGGTAGGACAAGGACAACAAATAGGACAGCTAGGACAAAGGCAACAACCA 1320

By9 TCTCTGCAGCAGGTAGGACAAGGACA**ACAAATAGGACAGCTAGGACAAA**GGCAACAACCA 1275

By15 TCTCTGCAGCAACCAGGACAAGGGCAACAAGGGCATTACCCAGCTTCTCTGCAGCAGGTA 1320

By15* TCTCTGCAGCAACCAGGACAAGGGCAACAAGGGCATTACCCAGCTTCTCTGCAACAGGTA 1275

By16 TC**CCTGCAGCAGGTAGGACAA**GGACAACAAATAGGACAGCTAGGACAAAGGCAACAACCA 1320

By18 TCTCTGCAGCAGGTAGGACAAGGACAACAAATAGGACAGCTAGGACAAAGGCAACAACCA 1320

By20 TCTCTGCAGCAACCAGGACAAGGGCAACAAGGGCATTACCCAGCTTCTCTGCAGCAGGTA 1320

** ******** ********* ****** * * ** *** ** *

By8 GGACAAGGGCAACAAACAAGACAAGGGCAACAACTAGAACAAGGGCAACAACCAGGACAA 1380

By9 GGACAAGGGCAACAAACAAGACAAGGGCAACAACTAGAACAAGGGCAACAACCAGGACAA 1335

By15 GGACAAGGACAACAAA-------------------------------------------- 1336

By15* GGACAAGGACAACAAA-------------------------------------------- 1291

By16 GGACAAGGGCAACAAACAAGACAAGGGCAACAACTGGAACAAGGGCAACAACCAGGACAA 1380

By18 GGACAAGGGCAACAAACAAGACAAGGGCAACAACTAGAACAAGGGCAACAACCAGGACAA 1380

By20 GGACAAGGACAACAAA-------------------------------------------- 1336

******** *******

By8 ------------------------------------------------------GGGCAA 1386

By9 ------------------------------------------------------GGGCAA 1341

By15 -------------------TAGGACAGCCAGGACAAAGGCAACAACCAGGACAAGGGCAA 1377

By15* -------------------TAGGACAGCCAGGACAAAGGCAACAACCAGGACAAGGGCAA 1332

By16 GGGCAACAAACAAGACAAGGGCAACAACTAGAACAAGGGCAACAACCAGGACAAGGGCAA 1440

By18 ------------------------------------------------------GGGCAA 1386

By20 -------------------TAGGACAGCCAGGACAAAGGCAACAACCAGGACAAGGGCAA 1377

******

By8 CAAACAAGACAAGGGCAACAACTAGAACAAGGGCAACAACCAGGACAAGGGCAACAAGGG 1446

By9 CAAACAAGACAAGGGCAACAACTAGAACAAGGGCAACAACCAGGACAAGGGCAACAAGGG 1401

By15 CAAACAGAACAAGGGCAACAACTAGAACAAGGGCAACAACCAGGACAAGGGCAACAAGGG 1437

By15* CAAACAGGACAAGGGCAACAACTAGAACAAGGGCAACAACCAGGACAAGGGCAACAAGGG 1392

By16 CAAACAAGACAAGGGCAACAACTAGAACAAGGGCAACAACCAGGACAAGGGCAACAAGGG 1500

By18 CAAACAAGACAAGGGCAACAACTAGAACAAGGGCAACAACCAGGACAAGGGCAACAAGGG 1446

By20 CAAACAGAACAAGGGCAACAACTAGAACAAGGGCAACAACCAGGACAAGGGCAACAAGGG 1437

****** ****************************************************

PS17-F

By8 TACTATCCAACTTCTCCACAACAGTCGGGACAAGGGCAACAACCAGGACAATCACAACAA 1506

By9 TACTATCCAACTTCTCCACAACAGTCGGGACAAGGGCAACAACCAGGACAATCACAACAA 1461

By15 TACTATCCAACTTCTCCACAACAGTCAGGACAAGGGCAACAACCAGGACAATCGCAACAA 1497

By15* TACTATCCAACTTCTCCACAACAGTCAGGACAAGGGCAACAACCAGGACAATCGCAACAA 1452

By16 TACTATCCAACTTCTCCACAACAGTCAGGACAAGGGCAACAACCAGGACAATCACAACAA 1560

By18 TACTATC**CAACTTCTCCACAACAGTCG**GGACAAGGGCAACAACCAGGACAATCACAACAA 1506

By20 TACTATCCAACTTCTCCACAACAGTCAGGACAAGGGCAACAACCAGGACAATCGCAACAA 1497

************************** ************************** ******

By8 CCAGGACAAGGGCAACAAGGGTACTACTCAAGTTCTCTACAACAGCCAGGACAAGGGCTA 1566

By9 CCAGGACAAGGGCAACAAGGGTACTACTCAAGTTCTCTACAACAGCCAGGACAAGGGCTA 1521

By15 CCAGGACAAGGGCAACAAGGGTACTACTCAACTTCTCTACAACAGCCAGGACAAGGGCAA 1557

By15* CCAGGACAAGGGCAACAAGGGTACTACTCAACTTCTCTACAACAGCCAGGACAAGGGCAA 1512

By16 CCAGGACAAGGGCAACAAGGGTACTACTCAACTTCTCTACAACAGCCAGGACAAGGGCAA 1620

By18 CCAGGACAAGGGCAACAAGGGTACTACTCAAGTTCTCTACAACAGCCAGGACAAGGGCTA 1566

By20 CCAGGACAAGGGCAACAAGGGTACTACTCAACTTCTCTACAACAGCCAGGACAAGGGCAA 1557

******************************* ************************** *

By8 CAAGGGCACTACCCAGCTTCTCTGCAGCAGCCAGGACAAGGACATCCAGGACAAAGGCAA 1626

By9 CAAGGGCACTACCCAGCTTCTCTGCAGCAGCCAGGACAAGGACATCCAGGACAAAGGCAA 1581

By15 CAAGGGCACTACCCAACTTCTCTGCAGCAGCCAGGACAAGGACATCCAGGACAAAGGCAA 1617

By15* CAAGGGCACTACCCAGCTTCTCTGCAGCAGTCAGGACAAGGACATCCAGGACAAAGGCAA 1572

By16 CAAGGGCACTACCCAGCTTCTCTGCAGCAGCCAGGACAAGGACATCCAGGACAAAGGCAA 1680

By18 CAAGGGCACTACCCAGCTTCTCTGCAGCAGCCAGGACAAGGACATCCAGGACAAAGGCAA 1626

By20 CAAGGGCACTACCCAACTTCTCTGCAGCAGCCAGGACAAGGACATCCAGGACAAAGGCAA 1617

*************** ************** *****************************

By8 CAACCAGGACAAGGGCAACAACCAGAACAAGGGCAACAACCAGGACAGGGGCAACAAGGG 1686

By9 CAACCAGGACAAGGGCAACAACCAGAACAAGGGCAACAACCAGGACAGGGGCAACAAGGG 1641

By15 CAACCAGGACAAGGGCAACAACCAGAACAAGGGCAACAACCAGGACAGGGGCAACAAGGG 1677

By15* CAACCAGGACAAGGGCAACAACCAGAACAAGGGCAACAACCAGGACAGAGGCAACAAGGG 1632

By16 CAACCAGGACAAGGGCAACAACCAAAACAAGGGCAACAACCAGGACAGGGGCAACAAGGG 1740

By18 CAACCAGGACAAGGGCAACAACCAGAACAAGGGCAACAACCAGGACAGGGGCAACAAGGG 1686

By20 CAACCAGGACAAGGGCAACAACCAGAACAAGGGCAACAACCAGGGCAGGGGCAACAAGGG 1677

************************ ******************* *** ***********

By8 TATTATCCAACTTCTCCGCAGCAGCCAGGACAAGGGAAACAACTAGGACAAGGGCAACAA 1746

By9 TATTATCCAACTTCTCCGCAGCAGCCAGGACAAGGGAAACAACTAGGACAAGGGCAACAA 1701

By15 TATTATCCAACTTCTCCGCAGCAGCCAGGACAAGGGAAACAACTAAGACAAGGGCAACAA 1737

By15* TATTATCCAACTTCTCCGCAGCAGCCAGGACAAGGGAAACAACTAGGACAAGGGCAACAA 1692

By16 TATTATCCAACTTCTTCGCAGCAGCCAGGACAAGGGAAACAACTAGGACAAGGGCAACAA 1800

By18 TATTATCCAACTTCTCCGCAGCAGCCAGGACAAGGGAAACAACTAGGACAAGGGCAACAA 1746

By20 TATTATCCAACTTCTCCGCAGCAGCCAGGACAAGGGAAACAACTAAGACAAGGGCAACAA 1737

*************** ***************************** **************

By8 GGGTACTACCCAACTTCTCCGCAACAGCCAGGACAAGGGCAACAACCAGGACAAGGGCAA 1806

By9 GGGTACTACCCAACTTCTCCGCAACAGCCAGGACAAGGGCAACAACCAGGACAAGGGCAA 1761

By15 GGGTACTACCCAACTTCTCTGCAACAGCCAGGACAAGGGCAACAACCAGGACAAGGGCAA 1797

By15* GGGTACTACCCAACTTCTACGCAACAGCCAGGACAAGGACAACAACCAGGACAAGGGCAA 1752

By16 GGGTACTACCCAACTTCTCCGCAACAGCCAGGACAAGGGCAACAACCAGGACAAGGGCAA 1860

By18 GGGTACTACCCAACTTCTCCGCAACAGCCAGGACAAGGGCAACAACCAGGACAAGGGCAA 1806

By20 GGGTACTACCCAACTTCTCTGCAACAGCCAGGACAAGGGCAACAACCAGGACAAGGGCAA 1797

****************** ****************** *********************

By8 CAAGGGCACTGCCCAACTTCTCCGCAGCAGACAGGACAAGCGCAACAACCAGGACAAGGC 1866

By9 CAAGGGCACTGCCCAACTTCTCCGCAGCAGACAGGACAAGCGCAACAACCAGGACAAGGC 1821

By15 CAAGGGCACTGCCCAACTTCTCCGCAACAGACAGGACAAGCGCAACAACCAGGACAAGGC 1857

By15* CAAGGGCACTGCCCAACTTCTCCGCAGCAGACAGGACAAGCGCAACAACCAGGACAAGGC 1812

By16 CAAGGGCACTGCCCAACTTCTCCGCAGCAGACAGGACAAGCGCAACAACCAGGACAAGGC 1920

By18 CAAGGGCACTGCCCAACTTCTCCGCAGCAGACAGGACAAGCGCAACAACCAGGACAAGGC 1866

By20 CAAGGGCACTGCCCAACTTCTCCGCAACAGACAGGACAAGCGCAACAACCAGGACAAGGC 1857

************************** *********************************

By8 CAACAAATAGGACAAGTGCAACAACCAGGACAAGGGCAACAAGGGTACTACCCAATTTCT 1926

By9 CAACAAATAGGACAAGTGCAACAACCAGGACAAGGGCAACAAGGGTACTACCCAATTTCT 1881

By15 CAACAAATAGGACAAGTGCAACAACCAGGACAAGGGCAACAAGGGTACTACCCAATTTCT 1917

By15* CAACAAATAGGACAAGTGCAACAATCAGGACAAGGGCAACAAGGGTACTACCCAATTTCT 1872

By16 CAACAAATAGGACAAGTGCAAGAACCAGGACAAGGGCAACAAGGGTACTACCCAATTTCT 1980

By18 CAACAAATAGGACAAGTGCAACAACCAGGACAAGGGCAACAAGGGTACTACCCAATTTCT 1926

By20 CAACAAATAGGACAAGTGCAACAACCAGGACAAGGGCAACAAGGGTACTACCCAATTTCT 1917

********************* ** ***********************************

PS17-R

By8 CTGCAACAGTCAGGACAAGGGCAACAGTCAGGACAAGGGCAACAATCAGGACAAGGACAC 1986

By9 CTGCAACAGTCAGGACAAGGGCAACAGTCAGGACAAGGGCAACAATCAGGACAAGGACAC 1941

By15 CTGCAGCAGTCAGGACAAGGGCAACAGTCAGGACAAGGGCAACAATCAGGACAAGGACAC 1977

By15* CTGCAGCAGTCAGGACAAGGGCAACAGTCAGGACAAGGGCAACAATCAGGACAAGGACAC 1932

By16 CTGCAACAGTCAGGACAAGGGCAACAGTCAGGACAAGGGCAACAATCAGGACAAGGACAC 2040

By18 CTGCAACAGTCAGGACAAGGGCAACAGTCAGGACAAGGGCAACAATCAG**AACAAGGACAC** 1986

By20 CTGCAGCAGTCAGGACAAGGGCAACAGTCAGGACAAGGGCAACAATCAGGACAAGGACAC 1977

***** ******************************************* **********

By8 CAACTAGGACAAGGGCAGCAATCAGGACAAGAGCAACAAGGCTACGACAACCCATACCAT 2046

By9 CAACTAGGACAAGGGCAGCAATCAGGACAAGAGCAACAAGGCTACGACAACCCATACCAT 2001

By15 CAACTAGGACAAGGGCAGCAATCAGGACAAGAGCAACAAGGCTACGACAACCCATACCAT 2037

By15* CAACTAGGACAAGGGCAGCAATCAGGACAAGAGCAACAAGGCTACGATAACCCATACCAT 1992

By16 CAACTAGGACAAGGGCAGCAATCAGGACAAGAGCAACAAGGCTACGACAACCCATACCAT 2100

By18 **CAACTAGGAC**AAGGGCAGCAATCAGGACAAGAGCAACAAGGCTACGACAACCCATACCAT 2046

By20 CAACTAGGACAAGGGCAGCAATCAGGACAAGAGCAACAAGGCTACGACAACCCATACCAT 2037

*********************************************** ************

By8 GTTAACACAGAGCAGCAAACGGCCAGCCCAAAGGTGGCAAAGGTGCAGCAACCCGCGACA 2106

By9 GTTAACACAGAGCAGCAAACGGCCAGCCCAAAGGTGGCAAAGGTGCAGCAACCCGCGACA 2061

By15 GTTAACACAGAGCAGCAAACGGCCAGCCCAAAGGTGGCAAAGGTGCAGCAACCCGCGACA 2097

By15* GTTAACACAGAGCAGCAAACGGCCAGCCCAAAGGTGGCAAAGGTGCAGCAACCCGCGACA 2052

By16 GTTAACACAGAGCAGCAAACGGCCAGCCCAAAGGTGGCAAAGGTGCAGCAACCCGCGACA 2160

By18 GTTAACACAGAGCAGCAAACGGCCAGCCCAAAGGTGGCAAAGGTGCAGCAACCCGCGACA 2106

By20 GTTAACACAGAGCAGCAAACGGCCAGCCCAAAGGTGGCAAAGGTGCAGCAACCCGCGACA 2097

************************************************************

By8 CAGCTGCCGATAATGTGTCGGATGGAGGGGGGCGACGCATTGTCGGCTAGCCAGTGA 2163

By9 CAGCTGCCGATAATGTGTCGGATGGAGGGGGGCGACGCATTGTCGGCTAGCCAGTGA 2118

By15 CAGCTGCCGATAATGTGTCGGATGGAGGGGGGCGACGCATTATCGGCTAGCCAGTGA 2154

By15* CAGCTGCCGATAGTGTGTCGGATGGAGGGGGGCGACGCATTGTCGGCTAGCCAGTGA 2109

By16 CAGCTGCCGATAATGTGTCGGATGGAGGGGGGCGACGCATTGTCGACCAGGCAGTGA 2217

By18 CAGCTGCCGATAATGTGTCGGATGGAGGGGGGCGACGCATTGTCGGCTAGCCAGTGA 2163

By20 CAGCTGCCGATAATGTGTCGGATGGAGGGGGGCGACGCATTGTCGGCCAGCCAGTGA 2154

************ **************************** *** * ** ******

**Fig. S5.** The original gel images shown in Figure 2.

**
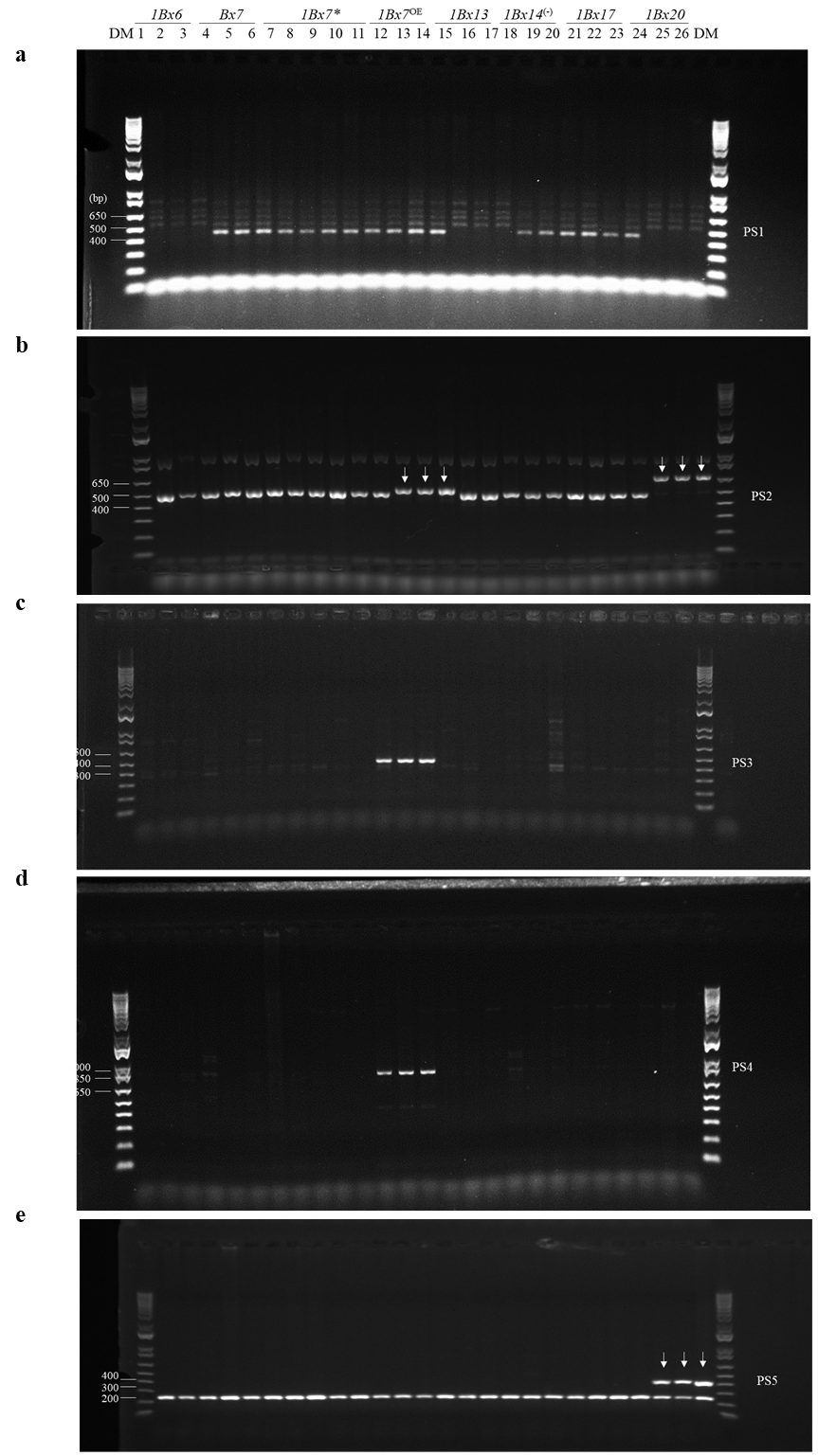
**


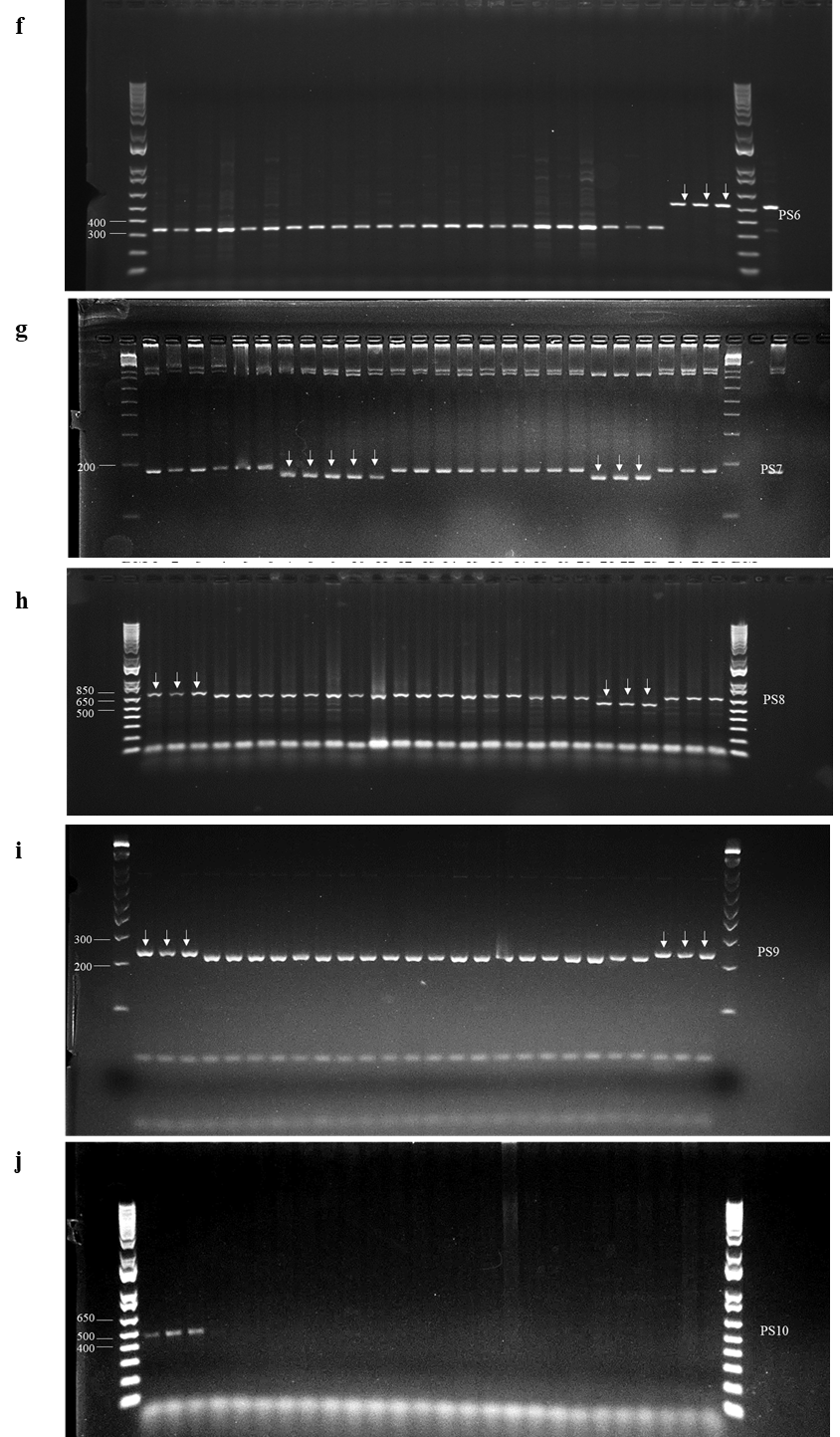


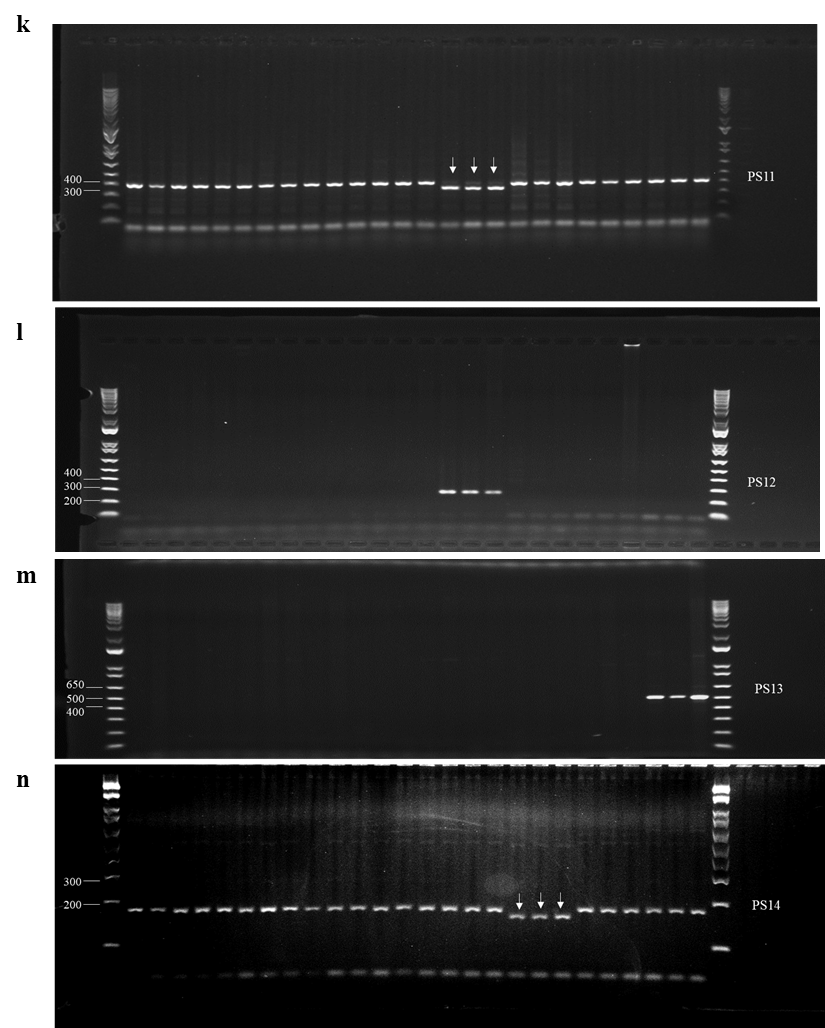


**Fig. S6.** The original gel images shown in Figure 3.


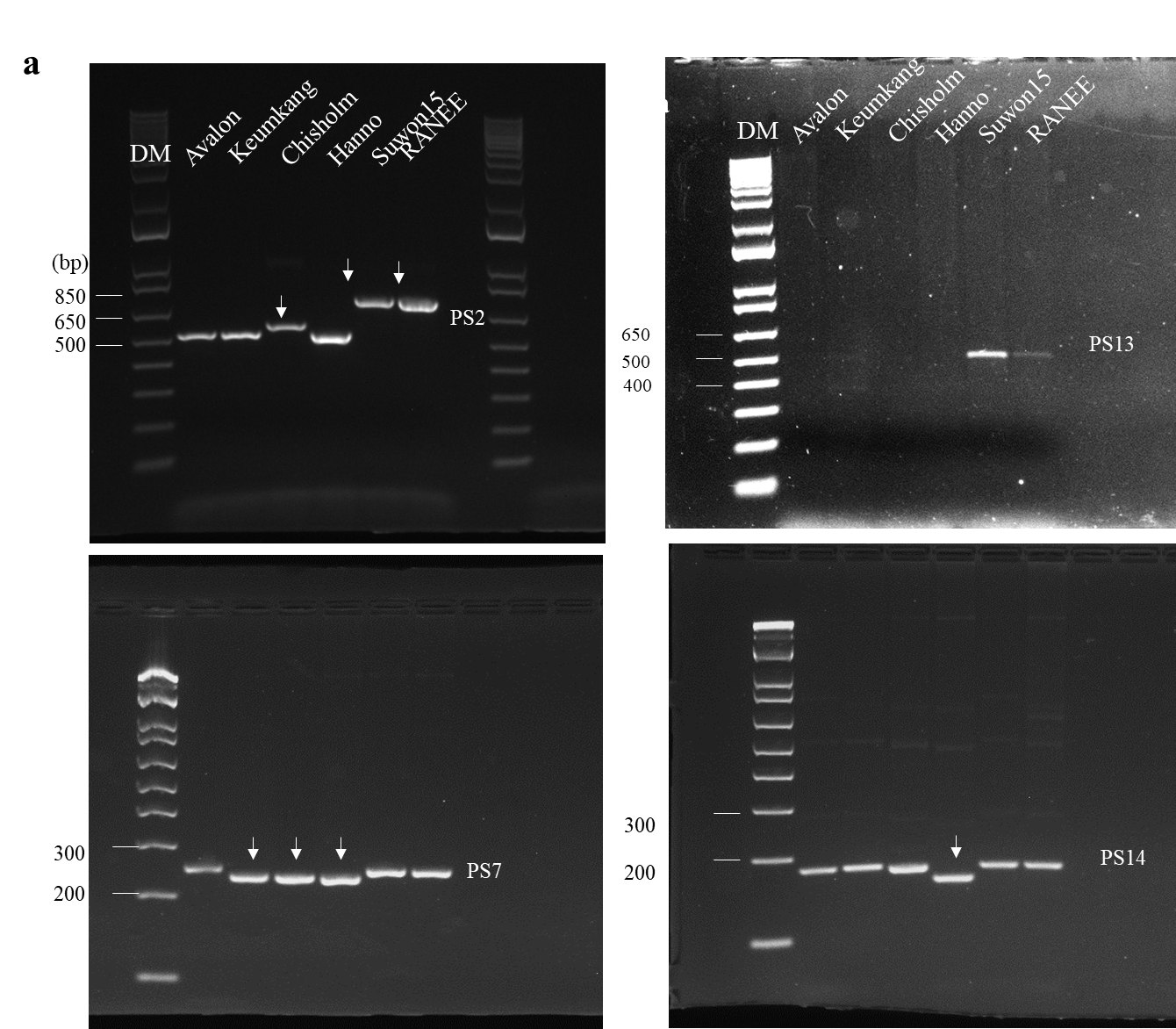


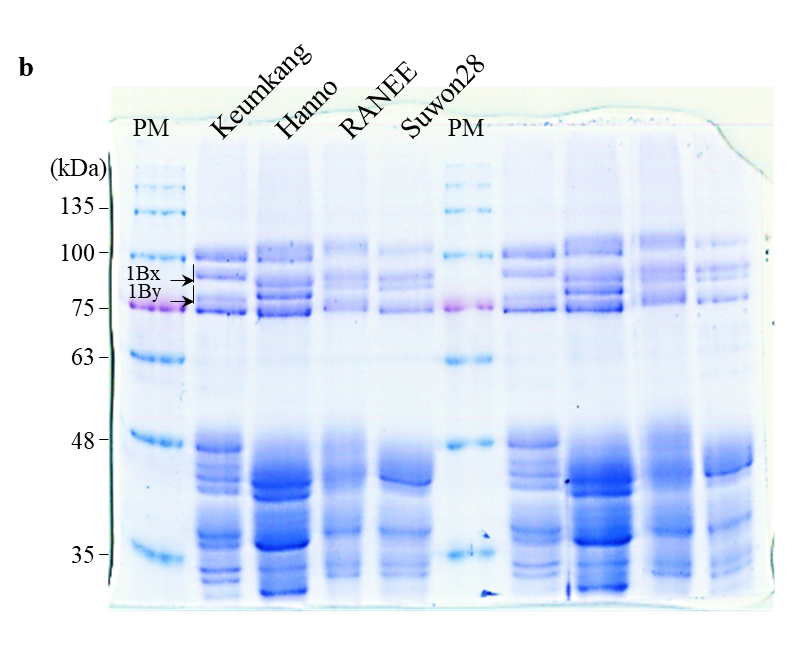


**Fig. S7.** The original gel images shown in Figure 5.


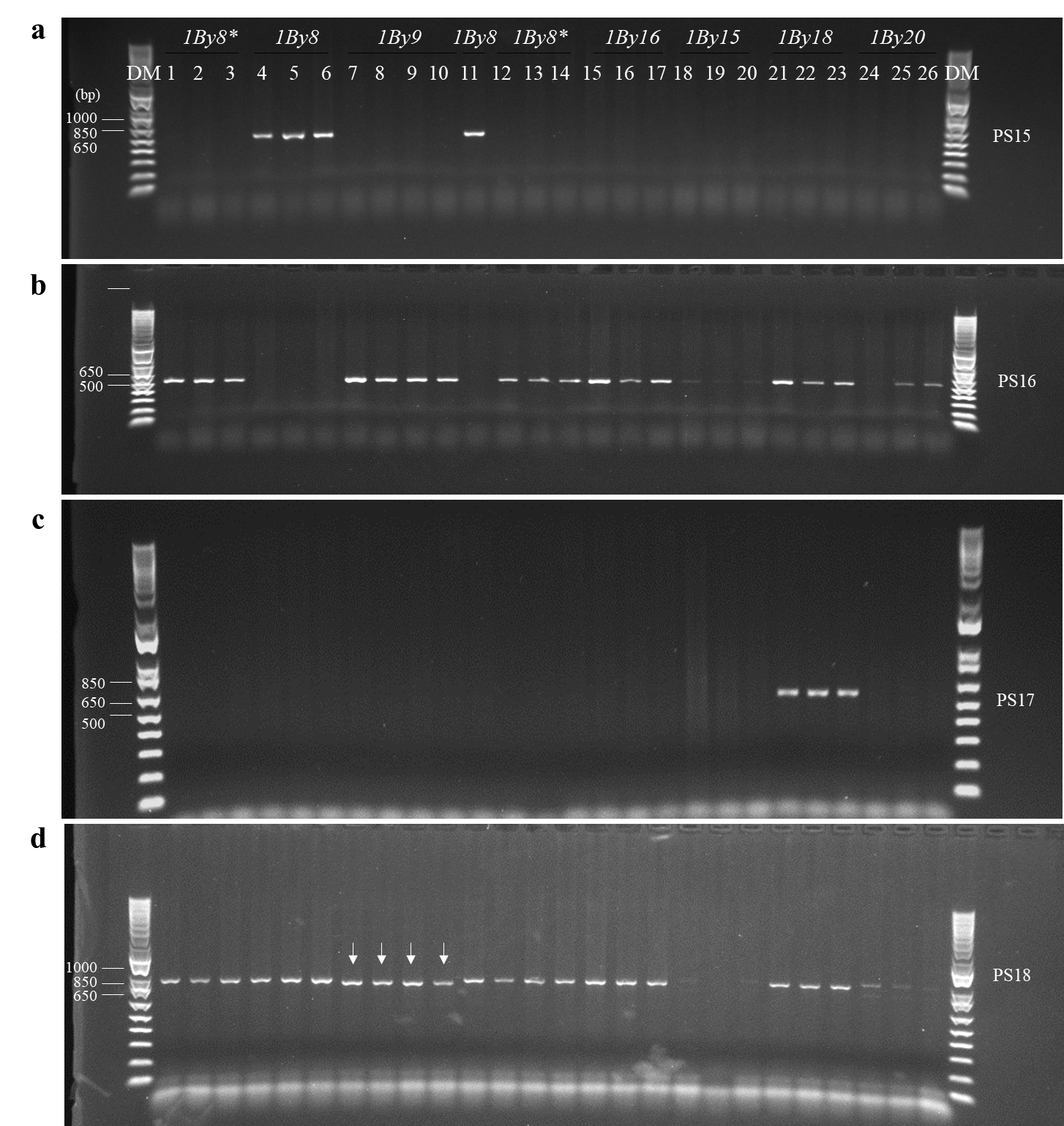


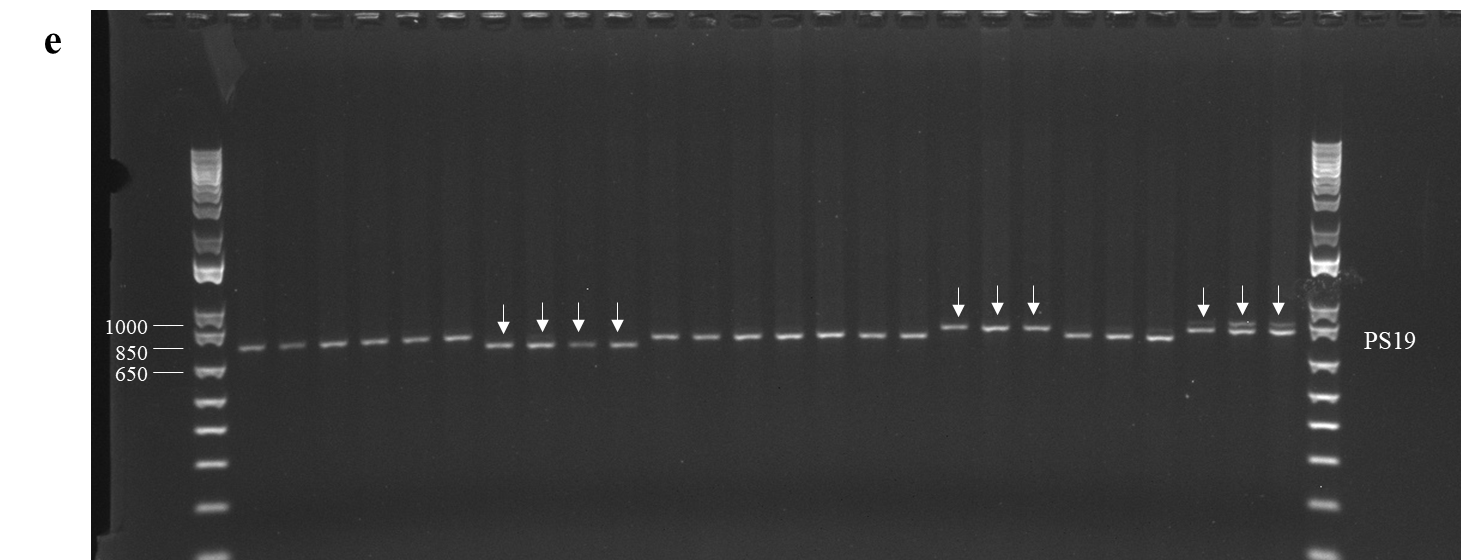


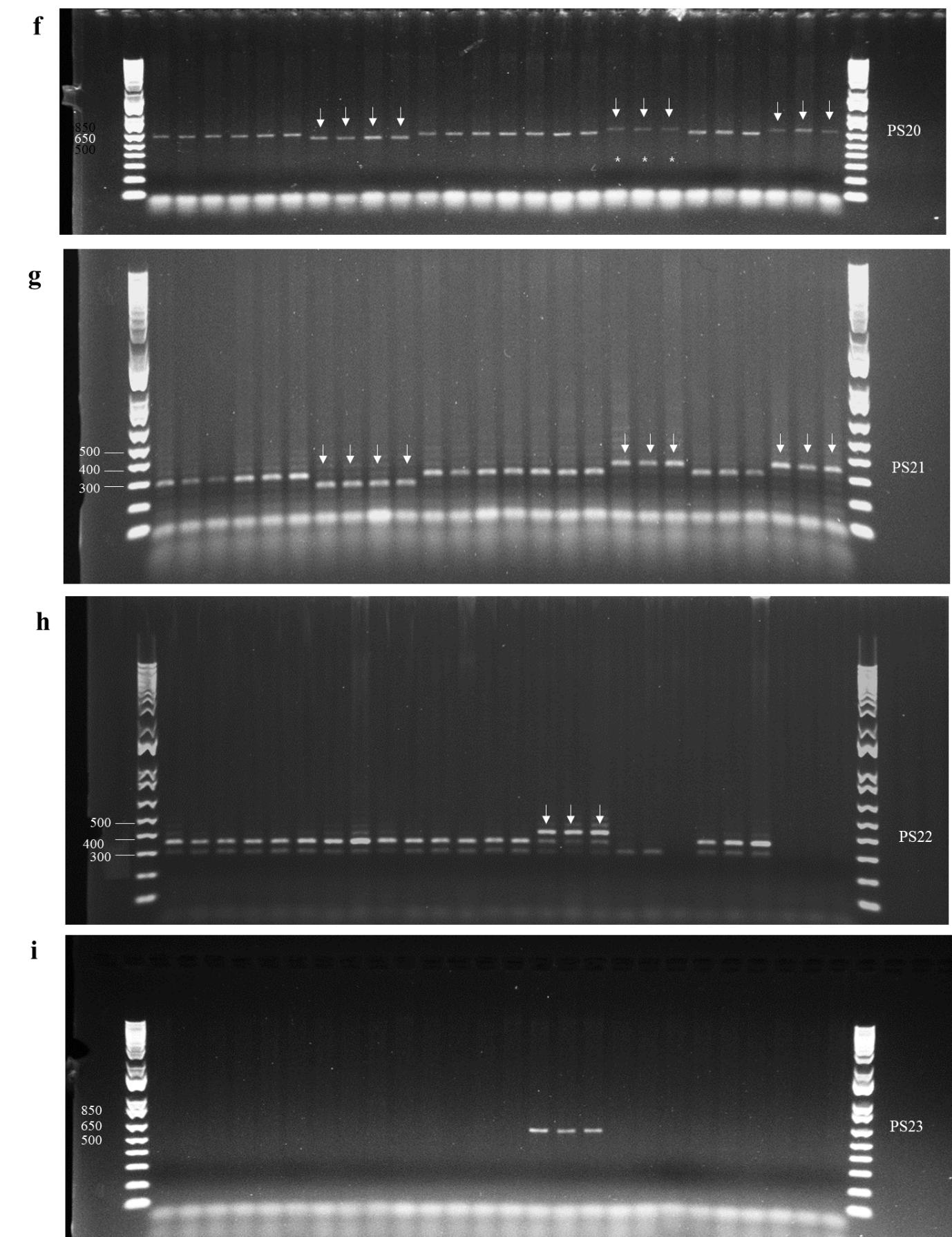


**Fig. S8.** The original gel images shown in Figure 7.

**
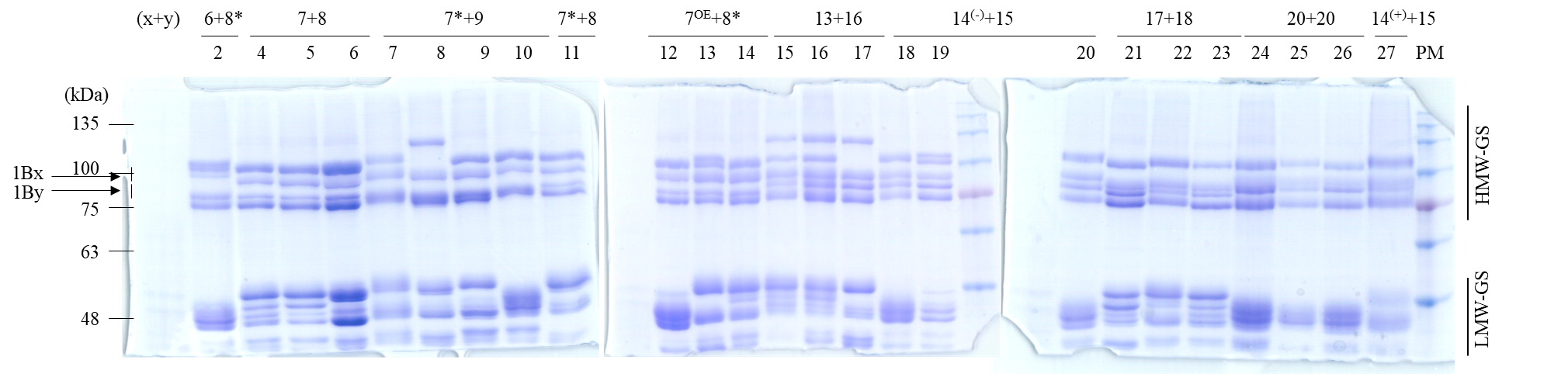
**
